# Loss-of-function variations in solute carrier family 38 member 6 are associated with essential tremor

**DOI:** 10.1038/s41392-025-02380-y

**Published:** 2025-09-11

**Authors:** Zhangqi Yuan, Qiying Sun, Junyu Luo, Lu Zhang, Yichi Zhang, Jifeng Guo, Cheng Wang, Kangjuan Yang, Shumin Yang, Yanjie Cao, Yinhua Shen, Jiaming Cui, Hengxiang Cui, Hao Sun, Tingbin Ma, Xuan Xu, Chun-Jie Liu, Tao Wang, An-Yuan Guo, Aifang Cheng, Luoying Zhang, Jun Liu, Man Jiang, Beisha Tang, Jing Yu Liu

**Affiliations:** 1 College of Life Science and Technology, Huazhong University of Science and Technology (HUST), Wuhan, Hubei China; 2 Department of Neurology, Xiangya Hospital, Central South University, Changsha, Hunan China; 3 Department of Neurology & Institute of Neurology, Ruijin Hospital affiliated to Medical College of the Shanghai Jiao Tong University, Shanghai, China; 4 Institute of Neuroscience, Center for Excellence in Brain Science and Intelligence Technology, Chinese Academy of Sciences, Shanghai, China; 5 Department of Cell Biology and Medical Genetics, Medical College of Yanbian University, Yanji, Jilin China; 6 Department of Continuing Medical Education, Tianjin Dongli District School of Advanced Health Education, Tianjin, China; 7 Shanghai Jiao Tong University School of Medicine, Shanghai, China; 8 Department of Neurology, Union Hospital of HUST, Wuhan, Hubei China; 9 Department of Biomedical Sciences, Faculty of Health Sciences, University of Macau, Taipa, Macao SAR China; 10 Department of Physiology, School of Basic Medicine and Tongji Medical College, HUST, Wuhan, Hubei China

**Keywords:** Neurological disorders, Medical genetics, Diseases of the nervous system

## Abstract

Essential tremor (ET) is a common neurological disease that is characterized by 4–12 Hz kinetic tremors of the upper limbs and high genetic heterogeneity. Although numerous candidate genes and loci have been reported, the etiology of ET remains unclear. A novel ET-related gene was initially identified in a five-generation family via whole-exome sequencing, and other variants were identified in 772 familial ET probands and 640 sporadic individuals via whole-genome sequencing. Among 71 (9.18%) Chinese families and 47 (7.34%) sporadic individuals with ET, we identified 15 types of protein-altering variants in solute carrier family 38 member 6 (*SLC38A6)*, which encodes sodium-coupled neutral amino acid transporter 6 (SNAT6) and is inherited in an autosomal dominant pattern. Over-expression of mutant SNAT6 for the three most common human mutations (p.Y108F, p.M281T and p.G318S) significantly impaired L-arginine (L-Arg) uptake in HeLa cells. The homozygous *Slc38a6* deletion mice *(Slc38a6*^-/-^) exhibited reduced L-Arg uptake in their cerebellar neurons, tremor, and cerebellar pathology. Slice electrophysiology revealed reduced neuronal Purkinje cell (PC) excitability and elevated inhibitory synaptic transmission in *Slc38a6*^-/-^ mice, in line with elevated “hairy” basket coverage around the PC soma. Furthermore, heterozygous *Slc38a6* deletion *(Slc38a6*^+/-^) and PC-specific *Slc38a6* deletion (*Slc38a6*^PC-/-^) mice also displayed tremor and PC abnormalities similar to those found in *Slc38a6*^-/-^ mice. These PCs displayed mitochondrial abnormalities and elevated ferroptosis markers (ACSL4, TFRC and Fe ions). In conclusion, we identified variants in *SLC38A6* that contribute ~8.35% to ET, generated mouse models displaying tremor, and delineated cerebellar cellular abnormalities and potential mechanisms underlying ET etiology.

## Introduction

Essential tremor (ET) is one of the most common neurological disorders. It is characterized by 4–12 Hz kinetic or postural tremors of the upper limbs, whereas other body parts, such as the head, vocal cords, and jaw, can also be affected.^1^ Although ET is considered a ‘benign’ disorder, its symptoms are progressive and potentially disabling.^2^ ET incidence is 1% among the general population, 5% for individuals older than 65 years, and over 20% for those older than 95 years.^3^ Moreover, ET is an important risk factor for Parkinson’s disease (PD), with affected individuals having nearly a fourfold increased risk of developing PD compared with the general population.^4^

In recent years, growing neuroimaging evidence has revealed that positive functional connectivity is significantly reduced in the cerebello–thalamo–cortical circuit of ET patients.^5^ Postmortem studies have shown that the cerebellum of ET patients exhibits multiple abnormalities, including Purkinje cell (PC) loss and abnormal axon/dendrite morphology, increased “hairy” basket coverage of the PC soma, reduced climbing fiber (CF)–PC synaptic inputs, and reduced GABA receptor levels in the dentate nucleus.^6–8^ Postmortem and neuroimaging studies have made important contributions to advancing our understanding of ET pathology, but research advancements are constrained by the limited availability of patient samples.^6^ The etiology of ET remains largely unclear. Both environmental and genetic factors have been shown to contribute to ET. Long-term exposure to environmental toxins, such as β-carboline alkaloids, lead, and pesticides, is associated with an increased risk of ET.^9^ The onset age of ET varies widely, with a bimodal distribution that peaks in the second and sixth decade and more than half of the affected individuals had a positive family history.^10^ Studies of large ET families and twins also indicate that genetic factors play important roles in the etiology of ET.^1^ Despite the high incidence of ET, the discovery of ET genes has been hampered by clinical heterogeneity, absence of reliable diagnostic markers, and the presence of nonpenetrance and phenocopies within familial ET patients.^11^ Nevertheless, studies using linkage analysis, genome-wide association studies (GWASs), case‒control associations, whole-exome sequencing (WES) and whole-genome sequencing (WGS) have provided strong evidence for the genetic basis of ET in many cases.^12,13^

Linkage analyses of ET families have identified three genetic loci, ETM1, ETM2, and ETM3, which are located at 3q13, 2p22-p25, and 6p23, respectively.^14–16^ Subsequent studies of ET families further identified the p.Ser9Gly variant in *DRD3* (ETM1)^14^ and the p.Ala265Gly variant in *HS1BP3* (ETM2)^15^ as heritable susceptible variants. WES analysis led to the identification of rare ET-associated variants in ten genes, including *FUS* (ETM4), *TENM4* (ETM5), *HTRA2*, *SCN4A*, *SORT1*, *KCNS2*, *NOS3*, *USP46*, *HAPLN4* and *TUB*.^17–20^ In addition, two genetic risk variants, rs9652490 and rs11856808 in *LINGO1*, are associated with ET on the basis of GWAS.^21^ Some common variants in *SLC1A2*, *STK32B*, *PPARGC1A*, and *CTNNA3* were also found to be associated with susceptibility to ET via GWAS.^22,23^ Recently, five independent genome-wide significant loci (rs1127215, rs17590036, rs28562175, rs1945016 and rs9980363) were reported to be associated with approximately 18% ET heritability via GWASs and meta-analyses.^24^ The GGC repeat expansion in *NOTCH2NLC* (ETM6) was found to segregate with approximately 5% Chinese ET families.^25^ Recently, a rare heterozygous variant in *TCP10L* (p.Arg90Glu) was identified in a multigeneration ET family.^26^ By using parametric linkage and nonparametric linkage (NPL) analysis of WGS data from 104 multigeneration European families with ET, 22 potential ET-related genes (*BTC, N6AMT1, PCDH9, EYA1, RBFOX1, MAPT, SCARB2, TUBB2A, VPS33B, STEAP1B, SPINK5, ZRANB1, TBC1D3C, PDPR, NPY4R, ETS2, ZNF736, SPATA21, ARL17A, PZP, BLK* and *CCDC94*) were identified with evidence of significant linkage.^27^ Although many candidate genes have been identified, no gene has yet been shown to play a primary role in ET. For most of these genes, the association could be detected in only one or a few families, with limited functional confirmation on the basis of animal studies. Notably, some existing genetic models fail to exhibit tremors or cerebellar pathology, likely due to species-specific limitations.^19,28^ Other transgenic models present motor phenotypes but also exhibit ataxia or dystonia, complicating the interpretation of ET-specific mechanisms.^29^ Pharmacological models, such as harmaline-induced tremors, replicate certain motor symptoms but do not mirror the structural or functional abnormalities of the inferior olivary nucleus (ION) observed in ET patients.^30^ Cerebellar degeneration models lack identification of genetic risk mutants in ET patients.^31–33^ Hence, the identification of genes and generation of animal models that are responsible for ET pathogenesis are important for understanding the major pathogenic mechanism of ET.

Herein, we identified *SLC38A6* variants in familial ET patients and sporadically affected individuals and revealed the relationships between cerebellar abnormalities and SNAT6 dysfunction. We generated *Slc38a6* deletion mouse models to mimic the tremors and pathological changes of ET, thereby revealing the underlying molecular mechanisms involved. This study provides novel molecular insights into the mechanisms underlying ET and promotes the development of new therapeutic drugs and treatments.

## Results

### Clinical features

**Fig. 1:**
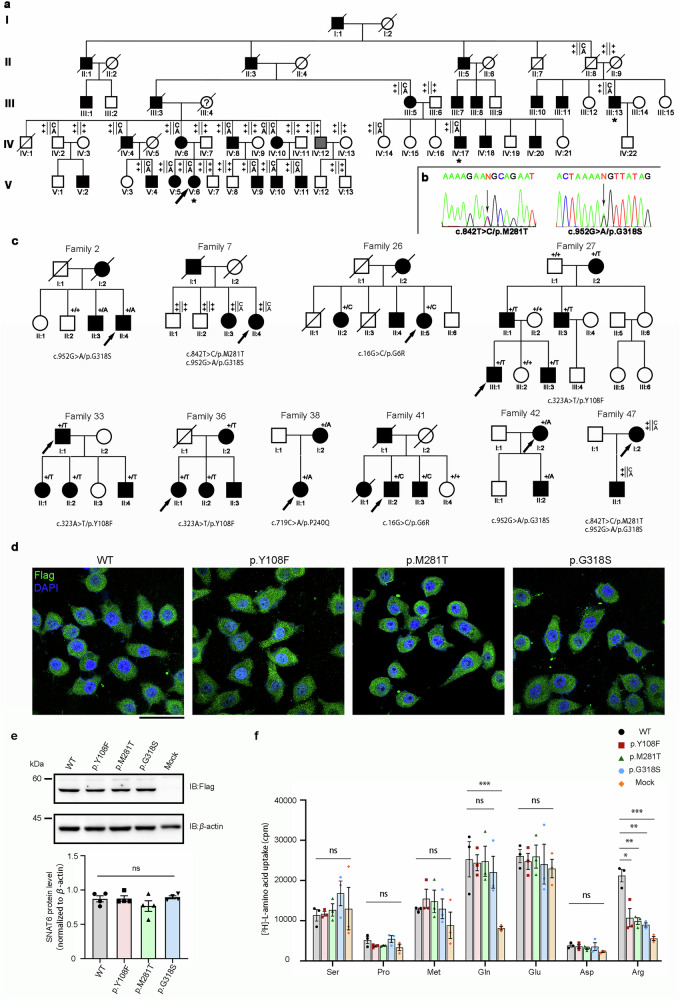
Identification of the *SLC38A6* gene in ET family 1 and the localization, expression level and amino acid transport function of SNAT6. **a** Pedigree of family 1 and the genotypes of the family members. Squares and circles represent males and females, respectively. The filled and blank symbols indicate affected subjects with ET and asymptomatic subjects, respectively. The arrow denotes the proband of family 1. The symbol with a question mark (?) represents an undefined individual, and the arrow indicates the proband. Asterisks (*) indicate the affected individuals chosen for whole-exome sequencing. The genotyping results of the two variants in the *SLC38A6* gene are shown for all the available family members. The variants c.842 T > C/p. M281T, and c.952 G > A/p. G318S are denoted with ‘C’ and ‘A’, respectively. ‘+’ indicates the reference allele. IV:2 and IV:14 carry heterozygous variants (c.842 T > C/p.M281T; c.952 G > A/p.G318S) in the *SLC38A6* gene but exhibit no clinical manifestations, suggesting the possibility of incomplete penetrance. IV:12 does not carry the heterozygous variants in the *SLC38A6* gene and presents only with postural tremors in both upper limbs. The TETRAS I score is 0, and the TETRAS II score is 4, suggesting a possible phenocopy. **b** Sanger sequencing results of the two variants of *SLC38A6* identified in Family 1. Variant loci are indicated with arrows. **c** Cosegregation of the *SLC38A6* genotype with the clinical phenotype in some families. **d** Representative immunofluorescence images of HeLa cells transfected with Flag-tagged SNAT6-WT and three variants (p.Y108F, p.M281T and p.G318S) and immunostained with anti-Flag (green: anti-Flag; blue: DAPI). **e** Quantification of the results of the West**e**rn blot analysis of Flag-tagged SNAT6-WT and three variants in HeLa cells. **f** Uptake of radiolabeled amino acids in HeLa cells overexpressing SNAT6-WT or three variants was measured in counts per minute (cpm) via scintillation counting. The data are presented as the means ± SEMs. Statistical tests: two-way ANOVA followed by Sidak’s multiple comparisons test (**e**), (**f**). Scale bar: 50 μm (**d**)

We collected and characterized data from 773 Chinese families and 640 sporadic cases of ET from mainland China. Among these, there was a five-generation large Chinese family with 61 members (Family 1) from Tianjin, including 27 patients (22 males and 5 females), who exhibited an autosomal dominant inheritance pattern (Fig. 1a). The proband V:6 developed posture and kinetic tremors affecting both upper limbs at the age of 20 and was assessed as 11 and 15.5, respectively, on the basis of the Essential Tremor Rating Assessment Scale (TETRAS)-I and TETRAS-II (Supplementary Table 1). The tremors of proband V:6 could be exacerbated by emotional stress, fatigue, or performing fine movements and relieved by drinking alcohol. The affected individuals in Family 1 exhibited action tremors in both upper limbs, such as the mother of the proband (IV:6) (Supplementary Video 1), whereas III:5, III:13 and IV:4 developed head and voice tremors. Individuals with cognitive impairment, dystonia, cerebellar ataxia, pyramidal signs and parkinsonism were excluded from Family 1 on the basis of neurological examinations. In addition, the brain MRI/CT scans of these patients were normal (Supplementary Table 1).

### Identification of *SLC38A6* variants in families with ET

To identify genetic variants associated with ET, WES ( >100-fold average coverage) was performed on three patients from Family 1, including the proband (V:6) and two other affected members (III:13, IV:17) (Fig. 1a). First, we screened 21 known ET candidate genes,^13–22,26,27,34,35^ including *DRD3, HS1BP3, LINGO1, FUS, SLC1A2, TENM4, HTRA2, SCN4A, SORT1, NOS3, KCNS2, HAPLN4, USP46, CACNA1C, STK32B, PPARGC1A, CTNNA3, LRRK2, SNCA, TUB* and *TCP10L*, but we did not find any mutations in these genes that segregated with the phenotype (excluding SNPs with a high population frequency >5% based on gnomAD), nor did we identify repeated expansions of *NOTCH2NLC* GGC via tandem repeat amplification analysis.^25^ We thus hypothesized the presence of novel pathogenic genes in Family 1. All variants were screened via the following criteria: (1) variants with a frequency less than 5% according to gnomAD; (2) missense variants that were predicted to be deleterious by at least 2 out of the 3 programs (Polyphen 2, SIFT, and CADD) or that are nonsense, splicing or frame-shift variants; and (3) considering the family history (suggestive of autosomal dominant inheritance), heterozygous variants need to be shared by three affected individuals. Six candidate rare variants in four genes (*DPP6*, *KMT5A*, *SLC38A6* and *GGT1*) met these criteria (Supplementary Table 2). Sanger sequencing and familial segregation testing of the available family members revealed that only the heterozygous variant c.842 T > C/p.M281T (rs117560154) and c.952 G > A/p.G318S (rs61050538) of *SLC38A6* (NM_001172702) (Fig. 1b) cosegregated with family 1, with frequencies of 0.0015 and 0.0021, respectively, in gnomAD (Genome Aggregation Database) for the total population and 0.0227 and 0.0278, respectively, in gnomAD for East Asians (Table 1). In addition, IV:10 is homozygous for the variant c.842 T > C/p.M281T and c.952 G > A/p.G318S of *SLC38A6* and suffered more severe tremor, whereas her mother (III:4) might also carry these two variants. II:8, IV:2, and IV:14 carried both of the variants but were asymptomatic, suggesting incomplete penetrance or a variable age of onset. IV:12 did not carry any of the variants of *SLC38A6* but resulted in mild tremor. He was diagnosed with a phenocopy because his symptoms did not worsen with age, and his offspring were normal.

**Table 1:** Mutation rates of *SLC38A6* (NM_153811) variants in this study

cDNA Alteration	Amino Acid Alteration	Chromosome Position^a^	rs ID^b^	Mutation rate in ET families	Mutation rate in Sporadic patient	Mutation rate in total patients	gnomAD_Total^c^	gnomAD_East Asian^d^	Polyphen	SIFT	CADD
c.16 G > C	p.G6R	chr14:60981293	rs184560042	0.0065	0.0063	0.0064	0.0005	0.0098	benign	deleterious	15.1
c.65 A > G	p.E22G	chr14:60981342	rs1305067041	-	0.0016	0.0007	-	0	benign	deleterious	23
c.323 A > T	p.Y108F	chr14:61015916	rs181093232	0.0168	0.0203	0.0184	0.0005	0.0088	probably_damaging	deleterious	26.4
c.350 G > A	p.G117E	chr14:61015943	rs749369988	-	0.0016	0.0007	0.0000637	0	probably_damaging	deleterious	29.1
c.428 T > C	p.I143T	chr14:61030469	rs781249288	-	0.0016	0.0007	-	-	probably_damaging	deleterious	25.4
c.619 C > T	p.L207F	chr14:61037678	-	0.0013	-	0.0007	-	-	possibly_damaging	tolerated	22.5
c.719 C > A	p.P240Q	chr14:61043478	rs145440362	0.0129	0.0047	0.0092	0.0005	0.0077	probably_damaging	deleterious	24.9
c.842 T > C	p.M281T	chr14:61046084	rs117560154	0.0440	0.0328	0.0389	0.0015	0.0227	probably_damaging	tolerated	25.9
c.952 G > A	p.G318S	chr14:61050538	rs74825213	0.0479	0.0359	0.0425	0.0021	0.0278	benign	tolerated	20.5
c.952_953GG > AA	p.G318N	chr14:61517257	-	0.0039	-	0.0021	-	-	probably_damaging	tolerated	17.22
c.1009 C > G	p.L337V	chr14:61050595	rs187227863	0.0013	0.0016	0.0014	-	0.0002	probably_damaging	deleterious	22.6
c.1105 C > T	p.R369C	chr14:61051841	rs201457210	0.0026	0.0031	0.0028	0.0001	0.0012	benign	deleterious	17.85
c.1357 T > C	p.W453R	chr14:61052415	rs1447682241	0.0026	0.0000	0.0014	-	0.0004	probably_damaging	deterious	21.1
c.565+1 G > A	-	chr14:61037142	rs759968619	-	0.0016	0.0007	0.00006	0.0002	NA	NA	33
c.744+1 G > A	-	chr14:61043504	rs200309894	-	0.0016	0.0007	0.00001	0.0004	NA	NA	34

^a^Position on Genome Reference Consortium human genome build 38 (GRCh38)

^b^ID in dbSNP

^c^The frequency of the variant in the entire population of the Genome Aggregation Database

^d^The frequency of the variant in the East Asian population of the Genome Aggregation Database

Polyphen 2 (version 2.2.3r408): Polymorphism phenotyping 2; SIFT (SIFT 4 G): Sorting Intolerant From Tolerant; CADD (GRCh38-v1.6): combined annotation-dependent depletion score. NA: not available

To identify variants in *SLC38A6*, WGS was conducted on the remaining 1 412 ET patients in this study, including 772 familial ET probands (family history: similar manifestations in immediate family members or siblings) and 640 sporadic cases (family history: no similar manifestations in immediate family members or siblings). Variants were selected on the basis of the following criteria: (1) a frequency less than 5% on the basis of gnomAD and (2) missense variants predicted to be deleterious by at least one of three tools (Polyphen-2, SIFT, and CADD) or nonsense, splicing, or frameshift variants. In total, 118 (8.35%) patients were found to carry an *SLC38A6* gene variant, including 71 (9.18%) familial ET probands and 47 (7.34%) sporadic cases (Supplementary Notes, Supplementary Tables 3 and 4). The clinical characteristics of these ET patients are detailed in Supplementary Table 5. As a result, 15 different *SLC38A6* variants were identified (Supplementary Fig. 1a), all of which were confirmed through Sanger sequencing (Supplementary Fig. 1b; primers for *SLC38A6* are shown in Supplementary Table 6). Subsequent follow-up and Sanger sequencing further revealed cosegregation of the *SLC38A6* genotype with clinical phenotypes in multiple families (Fig. 1c and Supplementary Table 7).

### *SLC38A6* variants impair arginine transport activity

To investigate the effects of *SLC38A6* variants, we selected three variants (c.323 A > T/p.Y108F, 23.7%; c.842 T > C/p.M281T, 33.1%; c.952 G > A/p.G318S, 37.3%), which were the most common of the 15 different types (Table 1) and were highly conserved (Supplementary Fig. 2). Flag-tagged wild-type (WT) human *SLC38A6* and its variants (at the putative extracellular C-terminus) were expressed in HeLa cells (Fig. 1d), and the unpermeabilized cells were immunostained with anti-Flag antibodies to examine the localization and expression of *SLC38A6*. We found no significant difference in either the localization or expression level between WT SNAT6 and SNAT6 variants (Fig. 1d, e).

SNAT6 is a member of the SLC38 family of sodium-coupled neutral amino acid transporters.^36^ To determine the substrate of SNAT6 and whether the identified *SLC38A6* variants impair the transport function of SNAT6, we individually expressed WT *SLC38A6* and three variants (encoding Y108F, M281T and G318S of SNAT6) in HeLa cells, which presented low constitutive SNAT6 expression and low baseline SNAT6 activity (see https://www.proteinatlas.org/). The transfected HeLa cells were incubated with seven types of ^3^H-labeled amino acids (L-Ser, L-Pro, L-Met, L-Gln, L-Glu, L-Asp or L-Arg). Compared with the blank control, WT-SNAT6 transfection resulted in specific uptake of L-Gln and L-Arg (Fig. 1f). Notably, transfection with SNAT6 variants resulted in significantly reduced uptake of L-Arg but similar uptake of L-Gln to that of WT-SNAT6 (mean uptake of L-Arg: Mock, 5573.3 ± 512.6; WT, 21213.7 ± 1543.3; Y108F, 10735.0 ± 2281.3, *p* = 0.015; M281T, 9899.0 ± 833.9, *p* = 0.007; G318S, 8937.3 ± 513.3, *p* = 0.003) (Fig. 1f). These results suggest that impaired transport of L-Arg by SNAT6 variants may be the cause of ET.

### Behavioral and morphological abnormalities in *Slc38a6* deletion mice

The above results suggest that loss-of-function mutations in *SLC38A6* might be a cause of ET. The SNAT6 protein is highly conserved between humans (NP_722518.2) and mice (NP_001032806.2), with 84% (384/457) amino acid identity (Supplementary Fig. 3). We thus generated *Slc38a6*^*-/-*^ mice via a “knockout-first” strategy (Supplementary Fig. 4a). The primary cerebellar neurons of *Slc38a6*^+/+^ and *Slc38a6*^-/-^ mice were cultured, and their uptake of L-Arg and L-Gln was measured. We found that the deletion of SNAT6 indeed affected the uptake of L-Arg but not L-Gln (Supplementary Fig. 4b), which is consistent with the in vitro results.

Compared with *Slc38a6*^+/+^ mice, *Slc38a6*^*-/-*^ mice developed tremors at 2 months of age (Supplementary Video 2). The tremors of *Slc38a6*^-/-^ mice worsened with age (Supplementary Videos 3–5), and the tremors of *Slc38a6*^-/-^ mice at 12.5 months of age worsened in both frequency and amplitude (Supplementary Video 6). We performed multiple behavioral assays on *Slc38a6*^+/+^ and *Slc38a6*^-/-^ mice at 2 or 8 months of age. In the open field test, *Slc38a6*^-/-^ mice at both ages spent a similar amount of time in the center as *Slc38a6*^+/+^ mice did (Supplementary Fig. 4c). The coordination and balance of *Slc38a6*^-/-^ mice did not differ from those of *Slc38a6*^+/+^ mice at either age in the balance beam or coat hanger tests (Supplementary Fig. 4d, e). *Slc38a6*^+/+^ and *Slc38a6*^-/-^ mice presented similar weights and gaits at both ages (Supplementary Fig. 4f‒k). Our results suggested that *Slc38a6* deletion in mice significantly increased tremor but had no effect on cognitive or other motor functions, revealing a causal link between *Slc38a6* and tremor, which may be attributed to L-Arg deficiency.

Since *Slc38a6*^-/-^ mice developed tremors, we investigated whether *Slc38a6*^-/-^ mice exhibited cerebellar morphological changes similar to those found in ET patients.

### Loss of PCs

**Fig. 2:**
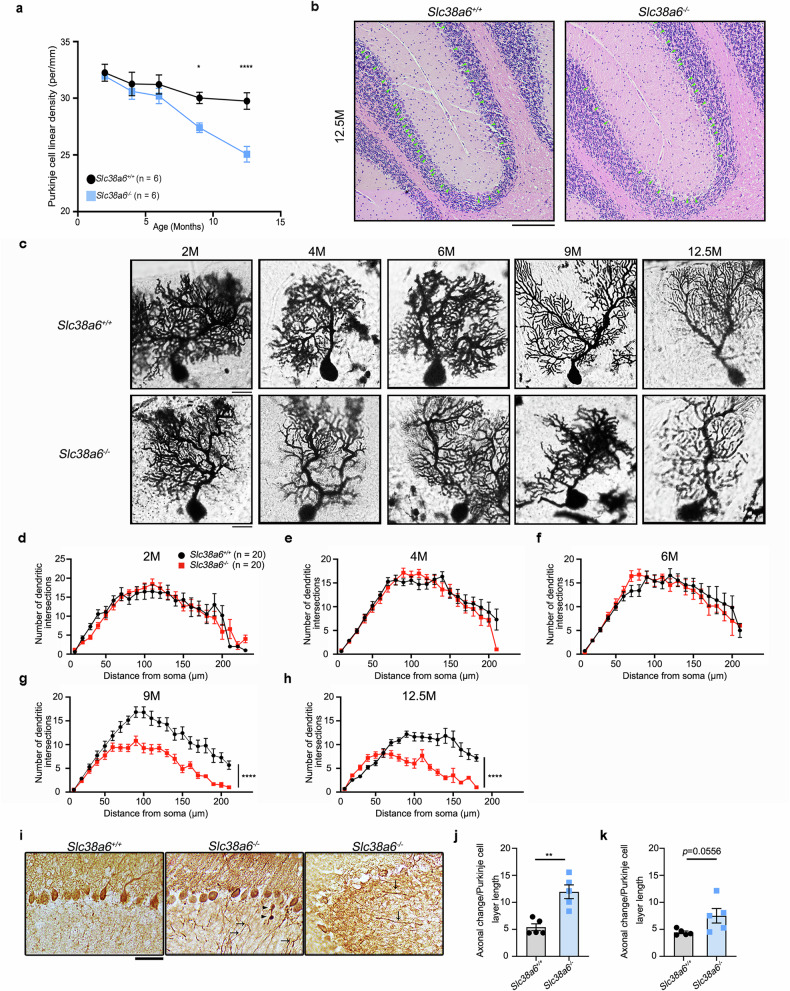
Soma, axonal and dendritic alterations of PCs in *Slc38a6*^-/-^ mice. **a** Quantification of the PC linear density of *Slc38a6*^+/+^ and *Slc38a6*^-/-^ mice at 2, 4, 6, 9, and 12.5 months (n = 6 mice per group). **b** Representative H&E-stained sagittal cerebellar sections of *Slc38a6*^+/+^ and *Slc38a6*^-/-^ mice at 12.5 months. **c** Golgi-stained PC dendrites in 2-, 4-, 6-, 9-, and 12.5-month-old *Slc38a6*^+/+^ and *Slc38a6*^-/-^ mice. **d**‒**h** Age-matched comparisons of dendrites of *Slc38a6*^+/+^ and *Slc38a6*^-/-^ mice at 2 (d), 4 (e), 6 (f), 9 (g), and 12.5 (h) months were performed via Sholl analysis (n = 20 stained PCs from 4 mice for each group). **i** Representative PC axons stained with calbindin-D28K. Abnormal alterations of PC axons in *Slc38a6*^-/-^ mice at 12.5 months. Left, normal PC axons in *Slc38a6*^+/+^ mice at 12.5 months; middle, two torpedoes (arrowheads) and branched axons (arrows); right, thickened axonal profiles (arrows). **j** Comparison of torpedoes between *Slc38a6*^+/+^ and *Slc38a6*^-/-^ mice at 12.5 months (n = 5 mice per group). **k** Comparison of thickened axonal profiles between *Slc38a6*^+/+^ and *Slc38a6*^-/-^ mice at 12.5 months (n = 5 mice per group). The data are presented as the means ± SEMs. Statistical tests: two-way ANOVA followed by Sidak’s multiple comparison tests (**a**), (**d‒h**) and the Mann‒Whitney test (**j**), (**k**). Scale bars: 50 μm (**b**); 20 μm (**c**); 50 μm (**i**)

To study PC degeneration with age, we first obtained 7 μm-thick paraffin sections of the cerebellums of *Slc38a6*^*+/*+^ and *Slc38a6*^*-/-*^ mice from 2 to 12.5 months of age via H&E staining. In 2-, 4-, and 6-month-old *Slc38a6*^*-/-*^ mice, the density of PCs was similar to that in *Slc38a6*^*+/*+^ mice (Fig. 2a; Supplementary Fig. 6). However, the PC density of 9-month-old *Slc38a6*^-/-^ mice was significantly lower than that of *Slc38a6*^*+/+*^ mice (*Slc38a6*^*+/+*^: 30.0 ± 0.5 per∙mm^-1^; *Slc38a6*^-/-^: 27.4 ± 0.4 per∙mm^-1^; *p* = 0.048) (Fig. 2a; Supplementary Fig. 5). This difference in PC density was significantly greater at 12.5 months of age (*Slc38a6*^*+/+*^: 29.8 ± 0.7 per∙mm^-1^; *Slc38a6*^-/-^: 25.1 ± 0.7 per∙ mm^-1^; *p* < 0.0001) (Fig. 2a, b). To evaluate the loss of PC, we performed dual IHC staining for calbindin-D28K and GAD. The presence or absence of PC soma in the GAD-labeled “basket” was defined as “full basket” or “empty basket”, respectively. We quantified the percentage of “empty baskets” to indirectly assess the loss of PCs and found that the percentage of empty baskets in *Slc38a6*^-/-^ mice was significantly greater than that in *Slc38a6*^+/+^ mice from 9‒12.5 months of age (Supplementary Fig. 6a‒c).

### Reduced complexity of PC dendrites

To evaluate the dendritic structures, we performed Golgi-Kopsch staining on 2- to 12.5-month-old *Slc38a6*^+/+^ and *Slc38a6*^-/-^ mice followed by Sholl analysis (Fig. 2c).^37^ We found that the dendritic complexity was not significantly different between *Slc38a6*^-/-^ and *Slc38a6*^+/+^ mice at 2, 4, and 6 months of age (Fig. 2d‒f). However, the maximum number of dendritic intersections was significantly reduced in 9-month-old *Slc38a6*^-/-^ (*Slc38a6*^*+/+*^: maximum value of 16.9 ± 1.1, peak at 90 μm; *Slc38a6*^-/-^: maximum value of 10.8 ± 1.0, peak at 90 μm, *p* < 0.0001) (Fig. 2g). At 12.5 months of age, the dendritic intersection curve of *Slc38a6*^-/-^ mice not only showed a reduced maximum but also shifted to the left (*Slc38a6*^*+/+*^: maximum value of 12.2 ± 0.6, peak at 90 μm; *Slc38a6*^-/-^: maximum value of 8.1 ± 0.8, peak at 60 μm, *p* < 0.0001) (Fig. 2h).

### Abnormal PC axon morphology

To assess morphological changes in PC axons, we performed IHC staining against calbindin-D28K in 12.5-month-old *Slc38a6*^*+/+*^ and *Slc38a6*^-/-^ mice, which exclusively stains the PC axons but omits the axons of interneurons, and observed alterations in the shape of PC axons (Fig. 2i), such as torpedoes (ovoid axonal swellings) and thickened axonal profiles. Notably, these abnormalities were greater in *Slc38a6*^-/-^ mice than in controls, but the torpedoes were significantly different (torpedoes: *Slc38a6*^*+/+*^, 5.4 ± 1.4; *Slc38a6*^-/-^, 12.0 ± 2.9; *p* = 0.0079) (Fig. 2j), and thickened axonal profiles were slightly different (thickened axonal profiles: *Slc38a6*^*+/+*^, 4.4 ± 0.6; *Slc38a6*^-/-^, 7.5 ± 3.0) (Fig. 2k).

### Increased “hairy” basket coverage of the PC soma

**Fig. 3:**
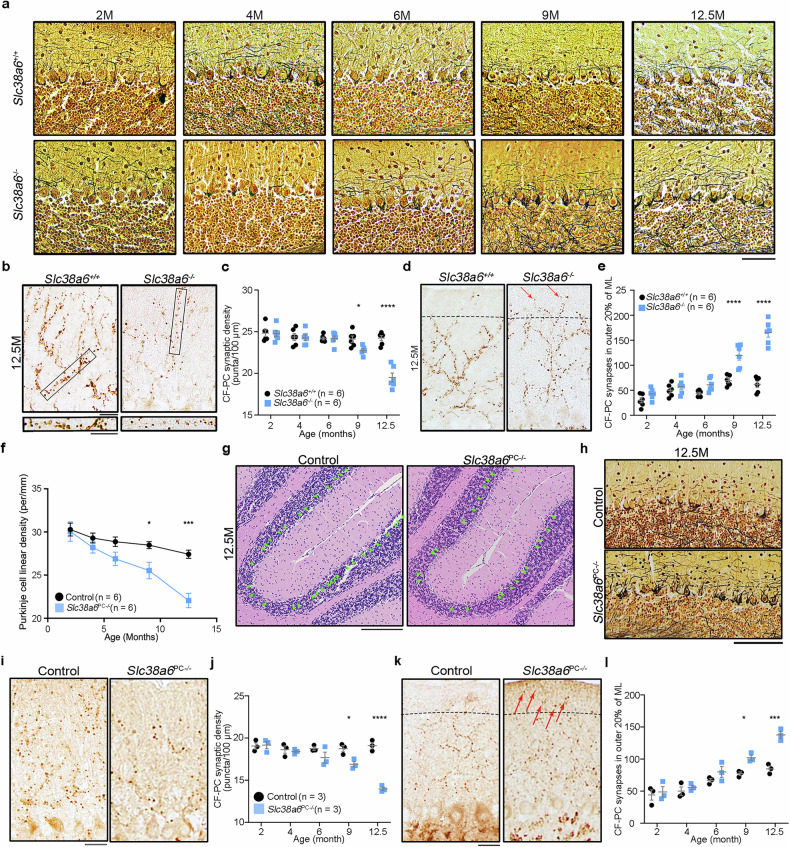
Cerebellar neuronal alterations in *Slc38a6*^*-/-*^ mice and *Slc38a6*^PC-/-^ mice. **a** Representative ‘hairy baskets’ of Bielschowsky silver-stained sections from *Slc38a6*^+/+^ and *Slc38a6*^-/-^ mice at 2, 4, 6, 9, and 12.5 months. **b** Representative CF‒PC synapses in the molecular layer of 12.5-month-old *Slc38a6*^+/+^ and *Slc38a6*^-/-^ mice labeled with vGluT2. The rectangular areas are shown below at a higher magnification. **c** Quantitative statistics of the CF synaptic density in *Slc38a6*^+/+^ and *Slc38a6*^-/-^ mice at 2, 4, 6, 9, and 12.5 months (n = 6 mice per group). **d** Representative CF‒PC synapses in the outer 20% of the molecular layer labeled with vGluT2 in 12.5-month-old *Slc38a6*^+/+^ and *Slc38a6*^-/-^ mice. The dotted line indicates the boundary of the outer 20% and inner 80% of the molecular layer. Red arrows indicate the CF-PC synapses extending into the PF territory. **e** Quantitative statistics of CF‒PC synapses in the outer 20% of the molecular layer in *Slc38a6*^+/+^ and *Slc38a6*^-/-^ mice at 2, 4, 6, 9, and 12.5 months (n = 6 mice per group). **f** Quantification of the PC linear density of control and *Slc38a6*^PC*-/-*^ mice at 2, 4, 6, 9 and 12.5 months (n = 4 mice per group). **g** Representative sagittal sections of control and *Slc38a6*^PC*-/-*^ mice at 12.5 months were stained with H&E. **h** Representative ‘hairy baskets’ of Bielschowsky silver-stained sections of control and *Slc38a6*^PC*-/-*^ mice at 12.5 months. **i** Representative image of abnormal CF‒PC synapses in the molecular layer from 12.5-month-old control and *Slc38a6*^PC-/-^ mice labeled with vGluT2. **j** Quantification of CF synaptic density in control and *Slc38a6*^PC*-/-*^ mice at 2, 4, 6, 9 and 12.5 months (n = 3 mice per group). **k** Quantitative statistics of CF‒PC synapses in the outer 20% of the molecular layer in control and *Slc38a6*^PC*-/-*^ mice at 2, 4, 6, 9, and 12.5 months (n = 3 mice per group). The dotted line indicates the boundary of the outer 20% and inner 80% of the molecular layer. Red arrows indicate the CF-PC synapses extending into the PF territory. **l** Quantification of CF‒PC synapses in the outer 20% of the molecular layer in control and *Slc38a6*^PC*-/-*^ mice at 2, 4, 6, 9 and 12.5 months (n = 3 mice per group). The data are presented as the means ± SEMs. Statistical tests: two-way ANOVA followed by Sidak’s multiple comparison tests (**c**), (**e**), (**f**), (**j**), (**l**). Scale bars: 20 μm (**a**), (**b**), (**d**), (**i**), (**k**); 200 μm (**g**); 100 μm (**h**)

To assess the morphology of basket cell axons surrounding PCs in *Slc38a6*^*+/+*^ and *Slc38a6*^-/-^ mice, Bielschowsky silver staining was performed (Fig. 3a).^38^ We classified hairy baskets into the following categories: low (1, minimum number of axonal collaterals), intermediate (2, moderate number of axonal collaterals) and high (3, numerous axonal collaterals). By semiquantitative rating of the basket cell plexus, we found that the average rating of *Slc38a6*^*+/+*^ and *Slc38a6*^-/-^ mice was not significantly different from that of 2- to 9-month-old mice and was significantly different at 12.5 months of age (*Slc38a6*^*+/+*^: 1.33 ± 0.21; *Slc38a6*^-/-^: 2.17 ± 0.31, *p* = 0.029) (Supplementary Table 8). To further investigate alterations in the processes of the basket cell plexus, we used another quantitative analysis. The axonal collaterals surrounding visible PCs in *Slc38a6*^-/-^ and *Slc38a6*^*+/+*^ mice were classified into four degrees: 0 (no detectable processes), 1 (thin processes), 2 (moderate processes) and 3 (dense twining processes) (Supplementary Fig. 7a‒d). We detected an increased number of basket cell plexuses at degrees 1 to 3 in *Slc38a6*^-/-^ mice compared with *Slc38a6*^+/+^ mice, and this difference became more significant with age (Supplementary Fig. 7e‒g).

### Abnormal CF‒PC synapses

In the cerebellum, CFs are critical for the regulation of PC physiology and motor behavior.^39^ To label the CF synapses, we performed IHC staining for vesicular glutamate transporter type 2 (vGluT2), a specific marker for CF synapses,^40^ on the cerebellar sections. We found that there were no significant differences between *Slc38a6*^*+/+*^ mice and *Slc38a6*^-/-^ mice at younger ages (from 2***‒***6 months). Compared with *Slc38a6*^*+/+*^, the CF***‒***PC synaptic density was significantly lower in *Slc38a6*^-/-^ mice at 9 and 12.5 months (Fig. 3b, c) (at 9 months, *Slc38a6*^*+/+*^: 24.1 ± 0.4, *Slc38a6*^-/-^: 22.8 ± 0.2, *p* = 0.042; at 12.5 months, *Slc38a6*^*+/+*^: 24.2 ± 0.3, *Slc38a6*^-/-^: 19.5 ± 0.5, *p* < 0.0001). We then examined the distribution of CF***‒***PC synapses by quantifying the number of vGluT2 puncta in the outer 20% of the molecular layer, which is the parallel fiber (PF) region, and found more extended CF synapses at all ages, with a significantly greater number at 9 and 12.5 months of age in *Slc38a6*^-/-^ mice than in *Slc38a6*^*+/+*^ mice (at 9 months, *Slc38a6*^*+/+*^: 69.2 ± 4.2, *Slc38a6*^-/-^: 119.5 ± 9.2, *p* < 0.0001; at 12.5 months, *Slc38a6*^*+/+*^: 62.0 ± 6.1, *Slc38a6*^-/-^: 165.5 ± 9.4, *p* < 0.0001) (Fig. 3d, e).

All of the morphological changes described above in the mutant mice were similar to those reported in cerebellar samples from ET patients, suggesting that SNAT6 dysfunction causes pathological changes in ET.

### Behavioral and morphological abnormalities in PC-specific *Slc38a6* deletion mice

To explore the role of SNAT6 in the cerebellum, we performed IHC against SNAT6 and found that it was widely expressed throughout the mouse brain but enriched in the PC layer of the cerebellum (Supplementary Fig. 8a), which was consistent with the expression pattern revealed by in situ hybridization.^41^ To examine the potential role of PCs in mediating tremor, we specifically deleted *Slc38a6* in the PCs of mice (*Slc38a6*^PC-/-^) (Supplementary Fig. 8b), which also exhibited tremors that worsened with age (Supplementary Video 7), similar to those in *Slc38a6*^-/-^ mice.

Moreover, we found that, compared with L7-Cre control mice, *Slc38a6*^PC-/-^ mice presented significant PC loss at 9 months of age, and the difference became more significant at 12.5 months of age (Fig. 3f, g). Abnormal PC axon morphology and reduced PC dendrites were observed in 12.5-month-old *Slc38a6*^PC-/-^ mice (Supplementary Fig. 8c, d). *Slc38a6*^PC-/-^ mice presented significantly increased “hairy” basket coverage of the PC soma at 12.5 months of age (Fig. 3h; Supplementary Table 9). Compared with control mice, *Slc38a6*^PC-/-^ mice at 9 and 12.5 months of age presented significantly lower CF‒PC synaptic density and more CF synapses extending into the PF territory (Fig. 3i‒l). Therefore, dysfunction of SNAT6 on PCs might play a role in the pathogenesis of ET.

### Reduced excitability and increased inhibitory inputs in PCs of *Slc38a6*^-/-^ mice and *Slc38a6*^PC-/-^ mice

**Fig. 4:**
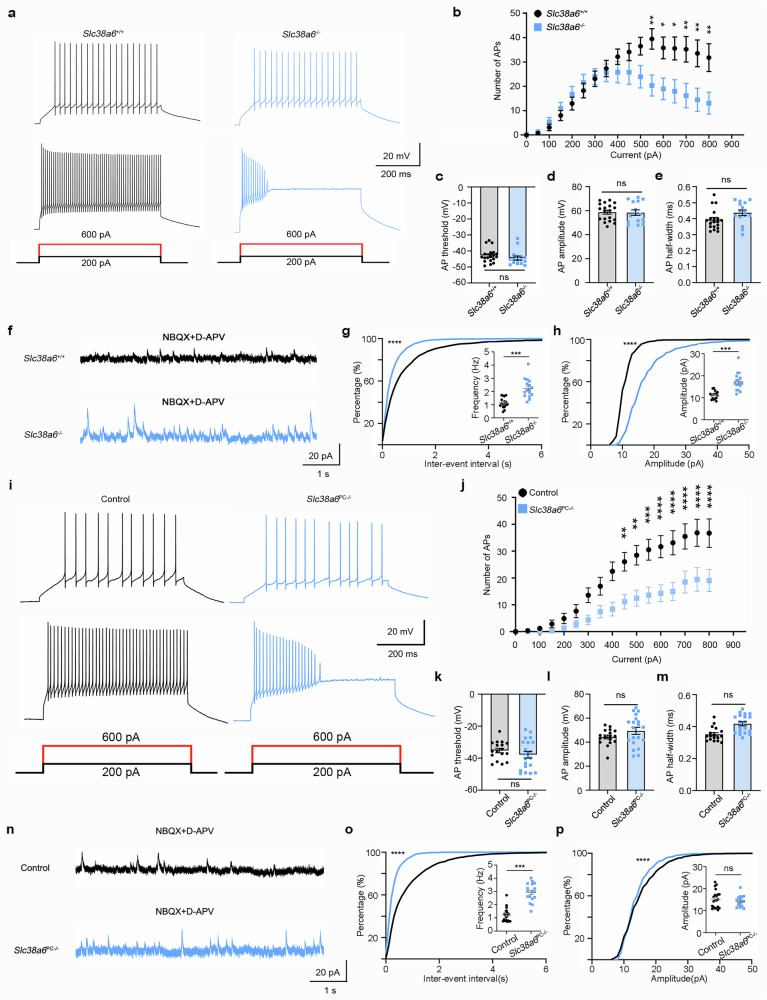
Abnormal functions of PCs in *Slc38a6*^-/-^ mice and *Slc38a6*^PC-/-^ mice. **a** Representative APs of *Slc38a6*^*+/+*^ and *Slc38a6*^*-/-*^ PCs evoked by current steps of 200 pA and 600 pA. **b** Number of APs induced by the current steps in *Slc38a6*^*+/+*^ (n = 19, black) and *Slc38a6*^*-/-*^ (n = 15, blue) PCs. **c–e** Summary of the threshold (**c**), amplitude (**d**) and half-width (**e**) of the APs in *Slc38a6*^*+/+*^ and *Slc38a6*^*-/-*^ PCs. **f** Representative sIPSC traces in *Slc38a6*^*+/+*^ and *Slc38a6*^*-/-*^ mice. NBQX is an AMPA receptor antagonist. D-APV is an NMDA receptor antagonist. **g** Cumulative distribution of interevent intervals and summary graphs of sIPSC frequency (inset) in *Slc38a6*^*+/+*^ (n = 17, black) and *Slc38a6*^*-/-*^ (n = 16, blue) PCs. **h** Same as panel (**g**) but for sIPSC amplitude. **i** Representative APs of control and *Slc38a6*^PC*-/-*^ PCs evoked by current steps of 200 pA and 600 pA. **j** Number of APs induced by the current steps in control (n = 17, black) and *Slc38a6*^PC*-/-*^ (n = 20, blue) PCs. **k‒m** Summary of the threshold (**k**), amplitude (**l**) and half-width (**m**) of the APs in control and *Slc38a6*^PC*-/-*^ PCs. **n** Representative sIPSC traces in control and *Slc38a6*^PC*-/-*^ mice. NBQX is an AMPA receptor antagonist. D-APV is an NMDA receptor antagonist. **o** Cumulative distribution of interevent intervals and summary graphs of sIPSC frequency (inset) in control (n = 21, black) and *Slc38a6*^PC*-/-*^ (n = 17, blue) PCs. **p** Same as panel (**o**) but for sIPSC amplitude. Note that sIPSC frequency was increased in *Slc38a6*^PC*-/-*^ PCs and that there was no change in sIPSC amplitude. The data are presented as the means ± SEMs. Statistical tests: two-way ANOVA followed by Sidak’s multiple comparison tests (**b**), (**j**); two-tailed unpaired Student’s *t* test (**c**‒**e**), (**k**‒**m**); Kolmogorov‒Smirnov test; Inner of (**g**), (**h**), (**o**), (**p**)

*Slc38a6*^*-/-*^ and *Slc38a6*^PC-/-^ mice developed tremors at an early age without any obvious morphological alterations in the tissues or cells of the cerebellum. We therefore speculated that dysfunction of SNAT6 might cause significant functional changes in PCs that precede morphological changes. We first analyzed the neuronal excitability of cerebellar PCs in 2-month-old *Slc38a6*^*+/+*^ and *Slc38a6*^*-/-*^ mice. Compared with that in *Slc38a6*^+/+^ mice, the number of action potentials (APs) evoked by depolarizing currents in PCs from *Slc38a6*^-/-^ mice was significantly lower, especially for larger currents (Fig. 4a, b). We also measured the threshold, amplitude and half-width of the first AP in response to depolarizing currents but did not find significant differences (Fig. 4c‒e), suggesting normal expression levels of voltage-gated sodium and potassium channels. To examine whether there was any alteration in the synaptic input from basket cells, spontaneous inhibitory postsynaptic currents (sIPSCs) were recorded (Fig. 4f). We found that both the frequency and amplitude of sIPSCs were significantly greater in *Slc38a6*^*-/-*^ mice than in WT mice (Fig. 4g, h). This could be attributed to the increased “hairy basket” around the PC soma in *Slc38a6*^*-/-*^ mice (Fig. 3a).

Similar electrophysiological changes, including impaired neuronal excitability (Fig. 4i‒m) and an increased mean frequency of sIPSCs (Fig. 4n, o), were also observed in *Slc38a6*^PC-/-^ mice, confirming the important role of SNAT6 in postnatal cerebellar PCs. However, we found that the average amplitude of sIPSCs in *Slc38a6*^PC-/-^ mice was not significantly different from that in WT mice (Fig. 4p), whereas a markedly increased amplitude was found in *Slc38a6*^-/-^ mice.

### Ferroptosis of PCs in *Slc38a6* deletion mice

**Fig. 5:**
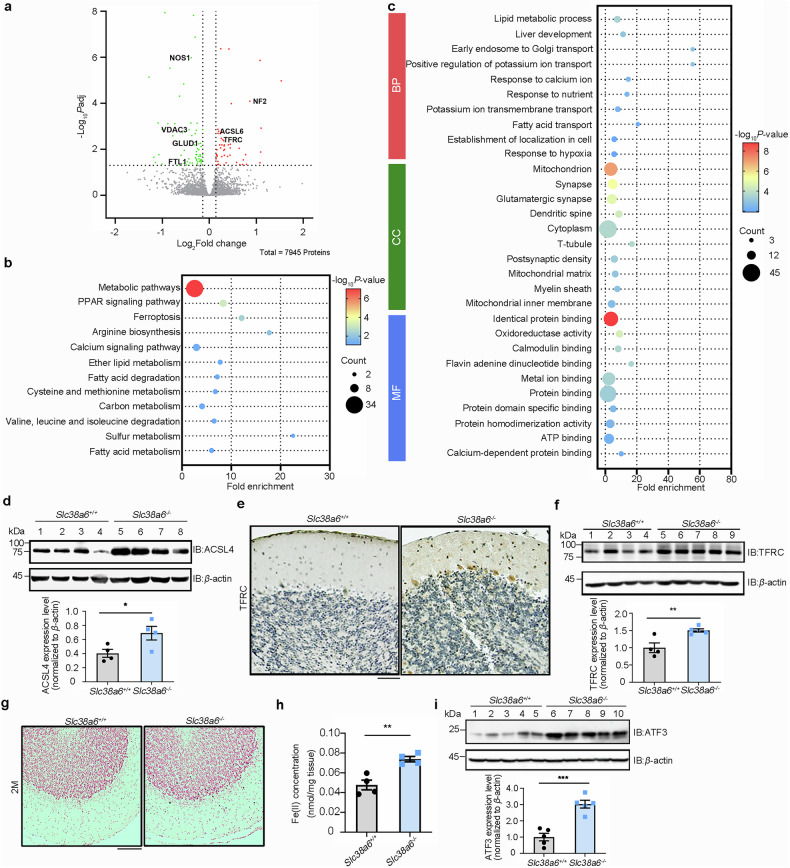
Proteomics and cerebellar ferroptosis analysis in 2-month-old *Slc38a6*^+/+^ and *Slc38a6*^*-/-*^ mice. **a** Volcano plot showing up- and downregulated proteins in 2-month-old *Slc38a6*^+/+^ and *Slc38a6*^*-/-*^ mice. Red indicates upregulated proteins, and green indicates downregulated proteins. **b‒c** KEGG enrichment (b) and GO enrichment (c) analyses of DEPs in *Slc38a6*^+/+^ and *Slc38a6*^*-/-*^ mice. The size of the dot indicates the number of proteins for each term, and the color indicates -log10 Padj for the KEGG and GO analyses. MF, Molecular Function; CC, Cellular Component; BP, Biological Process. **d** Expression levels of ACSL4 were measured in *Slc38a6*^+/+^ and *Slc38a6*^*-/-*^ cerebellums. **e** Representative images of TFRC immunostaining in *Slc38a6*^*+/+*^ and *Slc38a6*^*-/-*^ mouse cerebellums. **f** Same as panel (d) but for TFRC. **g** Representative Perls’ Prussian blue staining of *Slc38a6*^+/+^ and *Slc38a6*^*-/-*^ cerebellums. **h** Cellular iron levels in the *Slc38a6*^+/+^ and *Slc38a6*^*-/-*^ cerebellums. **i** Expression levels of ATF3 were measured in *Slc38a6*^+/+^ and *Slc38a6*^*-/-*^ cerebellums. The data are presented as the means ± SEMs. Statistical tests: two-tailed unpaired Student’s *t* test (**d**), (**f**), (**h**), (**i**). Scale bars: 50 μm (**e**), (**g**)

To explore the mechanism of PC alterations caused by SNAT6 deficiency at the young stage, we performed proteomic analysis on cerebellums from *Slc38a6*^+/+^ and *Slc38a6*^-/-^ mice via 4D-data-independent acquisition (DIA) mass spectrometry. A total of 7,945 proteins were identified, and we identified 128 differentially expressed proteins (DEPs) using an FDR-adjusted *P* value < 0.05 cutoff and a fold-change threshold of 1.1 (Fig. 5a, Supplementary Table 10). KEGG and GO enrichment analyses revealed that DEPs in the *Slc38a6*^+/+^ and *Slc38a6*^-/-^ cerebellums were involved mainly in ferroptosis and the mitochondrial, neuronal and synaptic pathways (Fig. 5b, c). To assess ferroptosis in the *Slc38a6*^-/-^ cerebellum, we measured the expression of the ferroptosis markers acyl-CoA synthetase long-chain family member 4 (ACSL4) and transferrin receptor (TFRC) and detected elevated levels of ACSL4 and TFRC in the *Slc38a6*^-/-^ cerebellum compared with those in the *Slc38a6*^+/+^ cerebellum (Fig. 5d‒f). The binding of extracellular Fe^3+^ to transferrin is known to be transported into the cell by TFRC.^42^ Prussian blue staining for total iron content (Fig. 5g) and an iron assay kit for Fe^2+^, which is known to be involved in ferroptosis, revealed that the iron content of the cerebellum was significantly greater in *Slc38a6*^-/-^ mice than in *Slc38a6*^+/+^ mice (Fig. 5h). Further Western blotting revealed that the level of ATF3 increased significantly in *Slc38a6*^-/-^ mice (Fig. 5i).

Increasing evidence suggests that mitochondria play a significant role in the execution of ferroptosis, and our proteomic analysis revealed that numerous DEPs were enriched in mitochondrial and metabolic pathways. We performed TEM analyses on cerebellar sections from 2-month-old *Slc38a6*^+/+^ and *Slc38a6*^-/-^ mice to examine whether there were ultrastructural alterations (Supplementary Fig. 9a). We detected several highly elongated mitochondria in *Slc38a6*^-/-^ PCs, which exhibited end‒to‒end connections, resulting in a doughnut-like morphology (Supplementary Fig. 9b), and the number of mitochondria and volume density, as well as the number of mitochondrial cristae, were significantly reduced in *Slc38a6*^-/-^ PCs (Supplementary Fig. 9c‒e). However, there was no change in the aspect ratio of the mitochondria or the length and width of the mitochondrial cristae (Supplementary Fig. 9f‒h). The changes in the morphology and number of mitochondria may be related to the impaired energy supply, leading to reduced neuronal firing in *Slc38a6*^-/-^ mice (Fig. 4b).

In summary, SNAT6 deficiency in the cerebellum promoted ferroptosis via ATF3 activation, contributing to abnormal metabolic and synaptic pathways in *Slc38a6*^-/-^ mice.

In addition, we also found that *Slc38a6*^+/-^ mice displayed ET-like tremors (Supplementary Videos 8 and 9) and exhibited significant morphological, electrophysiological or molecular alterations similar to those of *Slc38a6*^-/-^ mice (Supplementary Notes and Supplementary Fig. 10a‒k), phenocopying the behavioral and neurological symptoms observed in ET. Notably, we found that the cerebellums of *Slc38a6*^+/-^ mice expressed higher levels of ATF3 (Supplementary Fig. 10k), which is predicted to bind to the promoter of *Slc38a6* according to the JASPAR database. We further confirmed that ATF3 bound strongly to the *SLC38A6* promoter at two sites via ChIP assays (Supplementary Fig. 10l) and that the upregulation of ATF3 inhibited *SLC38A6* expression (Supplementary Fig. 10m). Therefore, we propose a dominant negative effect in *Slc38a6*^+/-^ mice, possibly due to the upregulation of ATF3.

## Discussion

In this study, we identified a novel candidate gene for ET, *SLC38A6*, via WES, WGS and Sanger sequencing of 71 families and 47 sporadic cases among 773 families and 640 sporadic cases diagnosed with ET, with a prevalence of approximately 8.35%. We found that L-Arg transport activity was dramatically impaired by the identified SNAT6 variants. *Slc38a6*^*-/-*^ and *Slc38a6*^PC*-/-*^ mice presented tremor and pathological alterations in the cerebellum, similar to what has been reported in the postmortem brains of ET patients. In addition, *Slc38a6* knockout resulted in severe electrophysiological and mitochondrial alterations in the PCs of 2-month-old mice. Finally, we found that ferroptosis developed in *Slc38a6* knockout PCs possibly via the upregulation of ATF3.

As a common neurological disease with high prevalence and heritability, the elucidation of candidate genes represents an important step in the diagnosis and treatment of ET.^43^ Many candidate genes and loci have been previously reported for ET families via linkage, WES, WGS or GWAS analyses.^13–27,34^ We performed WES, WGS and Sanger sequencing analyses on our ET patients and did not find any mutations in previously reported ET-associated genes. The incomplete penetrance (II:8, IV:2, and IV:14) and phenocopy (IV:1) of ET in family 1 could not be neglected, which initially puzzled us (Fig. 1a). We only found that mutations in *SLC38A6* cosegregated with those in conventional ET families (Fig. 1a). The mutations of *SLC38A6* appeared continuously with patients in each generation and in both sexes, and the affected or unaffected individuals arose equally in the offspring of each patient. Therefore, we concluded that the *SLC38A6* mutation was inherited in an autosomal dominant pattern, which is consistent with the observed phenotypes. We identified *SLC38A6* mutations in 118/1413 ( ~ 8.35%) of our ET patients of Han Chinese origin, and *SLC38A6* may be an important virulence gene of ET in the East Asian population.

One limitation of our study is that we observed that some individuals in our cohort were asymptomatic carriers of *SLC38A6* variants (Fig. 1a, c). Considering the onset age and incomplete penetrance of ET, we cannot exclude the possibility of developing ET in these carriers, and long-term follow-up of these carriers is needed to explain the role of *SLC38A6* in ET. Another limitation is that we attempted to identify *SLC38A6* for ET in other populations. However, the rarity of *SLC38A6* variants in the European population likely reflects ethnic-specific genetic architecture (Supplementary Table 4). This ethnic heterogeneity mirrors known disparities in ET-associated genes such as *FUS*, *LINGO1* and *NOTCH2NLC*, which might be due to the phenotypic and genetic heterogeneity of ET.^44–46^ In the future, larger multiethnic cohorts will be needed to fully characterize the role of *SLC38A6* in ET.

Although postmortem studies and animal models of tremor (mouse, rat, zebrafish, fruit fly, pig, and monkey) have advanced the understanding of ET,^29^ the etiology of ET is still unclear. The traditional drugs (harmaline, harmine and harmane) induce acute tremors and rhythmic bursting in ION of animal models, but structural or functional changes in the ION have not been observed in human postmortem studies.^30^ The currently available transgenic models, such as *Car8*^wdl^ mice and *ATXN2* deletion mice, may display a few core motor features, but they also exhibit prominent ataxia, dystonia or other neurological symptoms.^29^ Cerebellar degeneration models for ET, such as GABA_A_ receptor α1 deletion mice,^31^ GluRD2 dysfunction mice and synaptotagmin 2 deletion mice,^32,33^ are still limited in that they lack the genetic risk mutants found in ET patients. In addition, some ET models based on genetic studies, such as *hFUS-Q290X* in *Drosophila* and missense mutants of *TENM4* in zebrafish,^19,28^ do not display tremor or cerebellar pathological changes. In this study, we constructed *Slc38a6*^*-/-*^ and *Slc38a6*^PC*-/-*^ mouse models, which support the notion that *Slc38a6* mutation-induced cellular abnormalities worsen with age (Figs. 2, 3; Supplementary Fig. 5‒8) and mimic chronic pathophysiological changes in ET.^3,7,38,40,47^

Historically, tremor genesis in ET has been attributed to signal oscillations in the cerebellar‒thalamus‒motor cortex circuit, but the key pacemaker of tremor, whether in the ION, PC or deep cerebellar nucleus, remains controversial.^48^ In this study, we found that SNAT6 was enriched in the cerebellar PC layer. In addition, both *Slc38a6*^*-/-*^ and *Slc38a6*^PC-/-^ mice developed tremors and pathological changes, confirming the critical role of *Slc38a6* deficiency in the PC in mediating tremor. The orphan transporter SNAT6 is a member of the SLC38 family, which consists of 11 members, most of which are expressed in the brain and are involved in the transport of amino acids.^41^ SNAT6 was reported to be widely expressed in the brain via in situ hybridization, and mouse brain RNA-Seq confirmed this finding according to the Human Protein Atlas. SNAT6 was shown to transport L-Gln and L-Arg in a previous study,^36^ and we confirmed the function of SNAT6 in HeLa cells. Further analysis revealed that ET-associated SNAT6 variants impaired the transport of L-Arg but not L-Gln in both HeLa cells and primary cerebellar cells (Fig. 1e; Supplementary Fig. 4b). Furthermore, tremors developed in *Slc38a6*^*-/-*^ mice before any detectable abnormalities in cellular morphology were detected. We detected significant electrophysiological alterations in the PCs of 2-month-old *Slc38a6*^*-/-*^ and *Slc38a6*^PC-/-^ mice (Fig. 4). Changes in mitochondrial morphology, number and protein expression were also observed in 2-month-old *Slc38a6*^*-/-*^ mice (Fig. 5c; Supplementary Fig. 9), suggesting that PC dysfunction may be caused by an impaired energy supply. We therefore speculate that L-Arg is the primary substrate of SNAT6 and that there may be a link between L-Arg deficiency and PC dysfunction. PC dysfunction appears to precede cellular morphological changes and may induce signal oscillations transmitted in the cerebellum–thalamus–motor cortex circuit.

How does L-Arg deficiency result in PC dysfunction and even PC degeneration? We found that ATF3 was upregulated in the cerebellum of *Slc38a6*^-/-^ mice by Western blotting (Fig. 5i). As a stress-induced transcription factor, ATF3 promotes ferroptosis, which is driven by lipid peroxidation, iron handling, mitochondrial activity, and amino acid metabolism.^49,50^ We found that the hallmarks of ferroptosis—ACSL4, TFRC, and iron levels—were elevated in the *Slc38a6*^-/-^ cerebellum (Fig. 5d–h). Therefore, *Slc38a6* deletion may lead to L-Arg deficiency in cerebellar cells and activate ATF3. In addition, we found that upregulated ATF3 promotes ferroptosis and other PC dysfunctions by increasing lipid peroxidation and iron levels. Ferroptosis appears to be a major mechanism underlying PC loss, although other cell death pathways cannot be excluded. Notably, *Slc38a6*^+/-^ mice displayed ET-like tremors (Supplementary Videos 8 and 9) and exhibited significant morphological, electrophysiological and molecular alterations similar to those of *Slc38a6*^-/-^ mice (Supplementary Notes and Supplementary Fig. 10a–k), possibly due to the transcriptional repression of *Slc38a6* via the upregulation of ATF3. Therefore, we propose a dominant negative effect in *Slc38a6*^+/-^ mice due to the upregulated expression of ATF3.

**Fig. 6:**
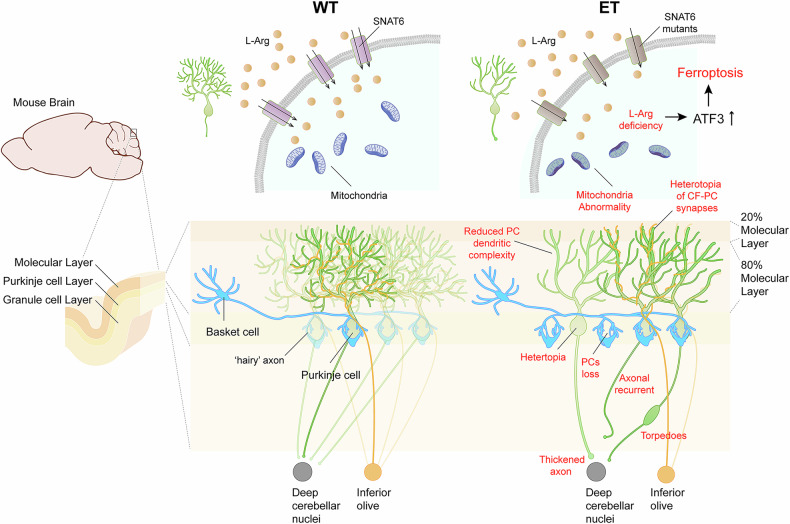
A working model for variants of *SLC38A6* causing ET. Loss-of-function variants of *SLC38A6* (encoding SNAT6) lead to arginine homeostasis alterations that result in ferroptosis, which in turn induces mitochondrial abnormalities and electrophysiological dysfunction in PCs. It affects the morphology of PCs, such as soma loss and heterotopia, thickened axons, recurrent axonal collaterals and torpedoes, reduced dendritic complexity, reduced CF**‒**PC synaptic density, and increased basket cell “hairy” axons as well as the function of PCs, leading to ET

In conclusion, we identified a novel ET-associated gene in ET patients and generated three *Slc38a6* deletion mouse models (*Slc38a6*^-/-^, *Slc38a6*^+/-^ and *Slc38a6*^PC-/-^) that displayed tremor and pathological changes in the cerebellum similar to those in the postmortem brains of ET patients, suggesting that *SLC38A6* loss-of-function variants may lead to ET symptoms. Further characterization revealed that the *SLC38A6* variants significantly altered cellular arginine homeostasis, resulting in functional and morphological changes in cerebellar cells, including PC degeneration and loss, as well as tremor phenotypes (Fig. 6).

## Materials and Methods

### Clinical subjects

This study included a total of 1413 patients with essential tremor (ET), all of whom were enrolled from the Chinese ET Registry (CETR) cohort. CETR is a multicenter registry study based on the Chinese population (ClinicalTrials.gov ID NCT04198246), comprising inpatients and outpatients from multiple neurology departments.^51^ Each patient underwent comprehensive history-taking, neurological physical examination, and tremor assessment scales performed by at least two experienced neurologists. The diagnostic criteria for ET were based on the guidelines of the Tremor Task Force of the International Parkinson and Movement Disorder Society as well as criteria from the Washington Heights-Inwood Genetic Study of Essential Tremor (WHIGET) to ensure diagnostic accuracy.^52,53^

Tremor evaluations included postural and kinetic tremor tasks, such as drinking water, pouring water, maintaining an extended arm posture, performing wing-beating postures, finger-nose-finger testing, writing, and spiral drawing. Each task was assessed separately for both upper limbs. The severity of tremor was evaluated via the Tremor Research Group Tremor Rating Assessment Scale (TETRAS).^54^ The TETRAS-I (items 1–12) assess the impact of tremors on daily living, with scores ranging from 0–4. The TETRAS-II (items 1–9) evaluate tremor type (including postural and kinetic tremors), tremor distribution (such as head, face, voice, limb, and trunk tremors), and tremor severity. Scores were assigned by physicians on the basis of tremor amplitude, with higher scores indicating greater tremor severity.

Patients with a history of caffeine or alcohol abuse, sympathomimetic medication use, or other factors potentially inducing tremor were excluded. Blood biochemical tests, including liver and kidney function, thyroid function, ceruloplasmin levels, and head MRI or CT scans, were performed to rule out other conditions causing tremor, psychogenic tremor, or enhanced physiological tremor.

All studies were conducted with the approval of the Ethics Committees of Huazhong University of Science and Technology (Wuhan, China), Xiangya Hospital of Central South University (Changsha, China), Ruijin Hospital of Shanghai Jiao Tong University School of Medicine (Shanghai, China) and the Center for Excellence in Brain Science and Intelligence Technology of the Chinese Academy of Sciences (Institute of Neuroscience) (Shanghai, China).

### DNA extraction for WES and WGS

Genomic DNA was extracted from peripheral blood via the Wizard Genomic DNA Extraction Kit (Promega). For WES, 3 μg of DNA was fragmented, and a library was prepared. Exons and flanking regions were captured via Agilent SureSelect Human All Exon V6 probes, and sequencing was performed on the Illumina HiSeq X platform with >100× coverage. The reads were aligned to the GRCh37 reference genome via BWA and SAMtools, and the variants were called via GATK and annotated via ANNOVAR. Variants were filtered for minor allele frequency (MAF) < 5% in public databases (gnomAD, 1000 Genomes, ExAC) and shared among affected individuals. Sanger sequencing was used for cosegregation analysis in family 1. For WGS, DNA was processed via the MGISEQ-2000 platform with a PCR-free protocol. Variants were generated via BWA, Picard, and GATK and annotated with ANNOVAR. *NOTCH2NLC* GGC repeat expansion was excluded by repeat-primed PCR.^25^

### Mutation analysis

Candidate genes were screened by WGS and Sanger sequencing in ET families and sporadic patients. The primers were designed via Primer3Plus and Primer-BLAST. PCR was performed on the exon sequences and flanking regions, and the products were sequenced via an ABI 3730XL sequencer. Variants were aligned manually via SeqMan (DNAStar).

### Cell culture and transfection

HeLa cells (ATCC, Manassas, USA) were grown in Dulbecco’s modified Eagle’s medium (DMEM; Gibco, New York, USA) supplemented with 10% fetal bovine serum (Gibco) at 37 °C under 5% CO_2_.

The *SLC38A6* sequence was cloned and inserted into a pcDNA3.1 vector with a Flag-tag at the C-terminus. Mutant plasmids (c.323 A > T/p.Tyr108Phe, c.842 T > C/p.Met281Thr and c.952 G > A/p.Gly318Ser) were generated via site-directed mutagenesis (primers are listed in Supplementary Table 11). When the cells were 80 ~ 90% confluent, they were transfected with Lipofectamine 2000 (Invitrogen, 116680) according to the manufacturer’s protocol.

### Immunofluorescence and immunoblotting

HeLa cells transfected with pcDNA3.1-SLC38A6-Flag or mutant plasmids were fixed in 4% PFA and stained with a rabbit anti-Flag antibody, followed by an Alexa Fluor 488-conjugated secondary antibody. Images were captured via a confocal microscope (Olympus FV3000). For immunoblotting, cells or mouse cerebella were lysed in RIPA buffer, and proteins were separated via gel electrophoresis and blotted with the following primary antibodies: Flag-tag (1:2000, rabbit polyclonal antibody; ABclonal, AE004), ATF3 (1:500, rabbit monoclonal antibody; Abcam, ab207434), ACSL4 (1:1000, rabbit monoclonal antibody; Abcam, ab155282), and TFRC (1:1000, mouse monoclonal antibody; Invitrogen, 136800). The membranes were captured with an Amersham Imager 600 and analyzed via ImageJ.

### Mice

*Slc38a6* deletion (*Slc38a6*^*-/-*^) mice were generated by the CAM-SU Genome Resource Center of Soochow University via the knockout-first strategy. The targeting vector containing a lacZ and Neo cassette flanked by Frt sites was introduced into embryonic stem (ES) cells via electroporation. *Slc38a6*-targeted ES clones (EPD0984_3_A01) on a C57BL/6 N background were aggregated with blastocysts from C57BL/6J-Tyrc-2J mice and implanted into pseudopregnant mice to generate F0 chimeras. The male chimeras were selected for coat color and backcrossed to purebred C57BL/6 N female mice to generate heterozygous mice (*Slc38a6*^Flox/WT^). Heterozygous male and female mice were mated to produce homozygous KO (*Slc38a6*^*-/-*^) and wild-type (WT/*Slc38a6*^*+/+*^) littermate control mice. PC-specific *Slc38a6* deletion mice, namely, *Slc38a6*^PC*-/-*^ mice, were obtained by crossing C57BL/6 N flp mice with *Slc38a6*^*-/-*^ mice followed by crossing with C57BL/6N-L7-Cre mice. All experiments followed protocols approved by the Ethics Committees of the Center for Excellence in Brain Science and Intelligence Technology of the Chinese Academy of Sciences (Institute of Neuroscience). All animals were bred and maintained in an SPF facility under a 12 h light‒dark cycle at 23 ± 0.5 °C, with *ad libitum* access to food and water. All behavioral assays were performed in accordance with previously reported methods; details can be found in the Supplementary Materials.

### ^3^H-labeled amino acid uptake assays

Wild-type and mutant *SLC38A6* plasmids were transfected into HeLa cells for 36 h. The cells were incubated in ^3^H-labeled amino acid mixture (1.6 μM unlabeled amino acids and 0.4 μM ^3^H-labeled amino acids diluted in Hank’s solution) for 30 min and then were lysed in 10% SDS. The lysates were mixed with scintillation liquid and measured as counts per min (cpm) in a Tri-Carb 4810TR liquid scintillation analyzer. Primary cerebellar neurons were dissociated from P2 *Slc38a6*^+/+^ and *Slc38a6*^-/-^ mice with papain solution and cultured in DMEM/F12. ^3^H-labeled glutamine and arginine uptake in primary cerebellar neurons was performed as described above. Details can be found in the Supplementary Materials.

### Tissue processing and morphological studies

*Slc38a6*^+/+^, *Slc38a6*^-/-^, *Slc38a6*^PC-/-^ mice and L7-Cre; *Slc38a6*^+/+^ mice (as a control for *Slc38a6*^PC-/-^ mice) were anesthetized via intraperitoneal injection of phenobarbital sodium (40 mg/kg) and then fixed in 4% paraformaldehyde solution at 4 °C overnight. After dehydration, the fixed cerebellum was embedded in paraffin for paraffin sectioning or in optimal cutting temperature (OCT) for frozen sectioning. Hematoxylin and eosin (H&E) staining for counting PC linear density, calbindin-D28k and glutamic acid decarboxylase (GAD) dual staining for analyzing the percentage of empty baskets, and vGluT2 immunohistochemistry (IHC) for determining the number and distribution of CF‒PC synapses were conducted on paraffin sections. Golgi staining and Bieschowsky staining were performed to assess the morphology of PC dendrites and basket cells, respectively. The frozen sections were stained with anti-SNAT6 to examine the localization of SNAT6 in the brain and with anti-calbindin-D28K to detect the axons of PCs. For ferroptosis analysis, TFRC IHC and Perls’ Prussian blue staining were performed on paraffin sections. All pathological staining and morphological studies were performed in accordance with previously reported methods, and details can be found in the Supplementary Materials.

### Slice electrophysiology

Sagittal cerebellar slices (250 μm) were prepared from 2-month-old mice. To record action potentials, whole-cell recordings from PCs in cerebellar lobules IV-VI were performed with patch pipettes (4–6 MΩ) and K-gluconate-based internal solution. To record sIPSCs, recordings from PCs were performed using CsMeSO3-based internal solutions with NBQX and D-APV in the bath solution. The signals were digitized and analyzed via Clampex and Mini-analysis. Details can be found in the Supplementary Materials.

### LC‒MS/MS analysis

Total protein was extracted from the cerebellum of *Slc38a6*^+/+^ or *Slc38a6*^-/-^ mice (three mice for each sample; each group contained three samples). For LC‒MS/MS analysis, the samples were analyzed via a TIMSTOF mass spectrometer (Bruker Daltonics, Bremen, Germany) combined with a high-performance nanoElute® chromatographic system (Bruker). Peptide separation was achieved via a custom-made reversed-phase analytical column (75 μm × 250 mm; 1.9 μm ReproSil-Pur C18 beads) and separated with a 60 min gradient of 2–80% mobile phase B at a flow rate of 300nL/min; mobile phases A and B were 0.1% (v/v) formate/ddH_2_O and 0.1% (v/v) formate/acetonitrile, respectively. Data-independent acquisition (DIA) parallel accumulation-serial fragmentation (PASEF) mode was used for the LC‒MS/MS analysis (Applied Protein Technology, Shanghai, China).

For bioinformatic analyses, protein expression counts obtained from mouse proteomics were first normalized. Hypothesis testing was performed via the R package DESeq2, and *P* values were adjusted for multiple testing by Benjamini and Hochberg. Proteins with FDR-adjusted *P* values < 0.05 and a fold change threshold of 1.1 were considered significantly altered.

### Assessment of Fe^2+^ levels

An iron assay kit (ab83366; Abcam) was used to assess the levels of Fe^2+^ in the cerebellar lysates of *Slc38a6*^+/+^ and *Slc38a6*^-/-^ mice according to the manufacturer’s protocol. A microplate reader (Multiskan SkyHigh, Thermo Scientific) was used to assess the absorbance of each well.

### Transmission electron microscopy (TEM)

Two-month-old mice were used for TEM analyses. The mice were anesthetized with phenobarbital sodium as described above and perfused with saline (pH 7.2), followed by 4% PFA and 0.5% glutaraldehyde in 0.1 M PB (pH 7.2). Cerebella were obtained and sliced into 100 μm sections with a vibratome. A 1 mm^3^ cube was dissected from the cerebellar sections, postfixed with 1% osmium tetroxide in 0.1 M cacodylate buffer (pH 7.4) for 1 h and dehydrated through a graded series of ethanol. The samples were embedded in Epon 812 after being rinsed in propylene oxide. Ultrathin sections (70 nm) were cut and stained with uranyl acetate and lead citrate. The PCs were selected randomly and photographed with a digital camera attached to a Hitachi 7500 electron microscope operated at 80 kV at a final magnification of 30,000×.

### Statistical analysis

Statistical analysis was performed with GraphPad Prism software. The statistical tests used are indicated in the figure legends.

## Data avilability

The raw sequence data reported in this paper have been deposited in the Genome Sequence Archive in National Genomics Data Center, China National Center for Bioinformation / Beijing Institute of Genomics, Chinese Academy of Sciences (GSA: HRA012503) that are publicly accessible at https://ngdc.cncb.ac.cn/gsa-human. The original DEPs and enrichment analysis results are included in Supplementary Data S1.

## Supplementary information

Supplementary Information

Supplementary Data S1

Supplementary Video 1

Supplementary Video 2

Supplementary Video 3

Supplementary Video 4

Supplementary Video 5

Supplementary Video 6

Supplementary Video 7

Supplementary Video 8

Supplementary Video 9

Dataset for fig 1 to 5

Dataset for supplementary
