# Supplementary material for: Loss-of-function variations in solute carrier family 38 member 6 are associated with essential tremor
Source: Signal Transduct Target Ther. 2025 Sep 11;10:296. doi: 10.1038/s41392-025-02380-y (PMC12423332; doi:10.1038/s41392-025-02380-y)
Supplement: Supplementary file 1 — Supplementary Information [file 41392_2025_2380_MOESM1_ESM.docx]

Supplementary Materials for

**Loss-of-function variations in solute carrier family 38 member 6 are associated with essential tremor**

Zhangqi Yuan, Qiying Sun, Junyu Luo, Lu Zhang, Yichi Zhang, Jifeng Guo, Cheng Wang, Kangjuan Yang, Shumin Yang, Yanjie Cao, Yinhua Shen, Jiaming Cui, Hengxiang Cui, Hao Sun, Tingbin Ma, Xuan Xu, Chunjie Liu, Tao Wang, Anyuan Guo, Aifang Cheng, Luoying Zhang, Jun Liu^*^, Man Jiang^*^, Beisha Tang^*^, Jing Yu Liu^*^

^*^Correspondence to: [liujy@ion.ac.cn](mailto:liujy@mail.hust.edu.cn); [bstang7398@163.com](mailto:bstang7398@163.com); [manjiang@hust.edu.cn](mailto:manjiang@hust.edu.cn) or jly0520@hotmail.com

**This file includes:**

Materials and Methods

Supplementary Notes

Supplementary Figures 1-10

Supplementary Tables 1-11

Supplementary Video 1. Recording for IV:6 of family 1

Supplementary Video 2. Recording for 2 month-old *Slc38a6*^-/-^ mouse

Supplementary Video 3. Recording for 4 month-old *Slc38a6*^-/-^ mouse

Supplementary Video 4. Recording for 6 month-old *Slc38a6*^-/-^ mouse

Supplementary Video 5. Recording for 9 month-old *Slc38a6*^-/-^ mouse

Supplementary Video 6. Recording for 12.5 month-old *Slc38a6*^-/-^ mouse

Supplementary Video 7. Recording for 12.5 month-old *Slc38a6*^PC-/-^ mouse

Supplementary Video 8. Recording for 9 month-old *Slc38a6*^+/-^ mouse

Supplementary Video 9. Recording for 12.5 month-old *Slc38a6*^+/-^ mouse

**Materials and Methods**

**DNA extraction for WES and WGS**

Genomic DNA was extracted from the peripheral blood of participants using the Wizard Genomic DNA extraction kit (Promega, USA). WES was carried out in proband and two other affected members of family 1. Firstly, genomic DNA (3 μg) was fragmented and a DNA library was prepared. Exons and flanking splicing regions were enriched and captured by Agilent SureSelect Human All Exon V6 probes (Agilent, USA). Finally, DNA was sequenced on the Illumina HiSeq X platform (Illumina, San Diego, CA), and the average sequencing depth was over 100×.

Reads that did not meet quality control requirements in the original raw data were eliminated, and sequences were then aligned to the human reference genome (GRCh37, UCSC version) and sorted by the software BWA (Burrows Wheeler Aligner) and Samtools. After removing duplicates from the sorted alignment using Picard, variants including single-nucleotide variants (SNVs) and small indels were called and annotated by the GATK (Genome Analysis Toolkit) pipeline and Annovar. First of all, a series of known ET causing genes and susceptible genes were analyzed. Subsequently, all variants were filtered with the criteria of minor allele frequency (MAF) < 5% in the public general population (gnomAD database, 1000 Genomes, ExAC database), and the variants should be shared by the three subjects undergoing exome sequencing. Further screening primarily targeted heterozygous exonic missense variants that were predicted to be damaging by PolyPhen-2, SIFT, and CADD. Specifically, variants with a CADD PHRED score exceeding 20 were classified as deleterious. Additionally, nonsense variants, splicing variants, and coding indels were also included in the analysis. For co-segregation analysis, Sanger sequencing of the candidate variants was conducted in all the available individuals in family 1.

WGS was performed using the MGISEQ-2000 platform following these major steps: (1) DNA fragmentation, (2) end repair and A-tailing, (3) indexed adapter ligation, (4) PCR amplification, (5) single-stranded circle (ssCir) formation, and (6) DNB preparation, which used PCR-free protocol (MGIEasy FS PCR-Free DNA Library Prep Set, omitting the PCR steps), as described in a previous study.^1^ Similarly, BWA, Picard, and GATK were used to generate high-quality variants. Then, ANNOVAR was used to annotate the variants. Specifically, *NOTCH2NCL GGC* repeat expansion was excluded by repeat-primed PCR in probands of these families and sporadic individuals with ET.^1^

**^3^H labeled amino acid uptake assays**

Cerebellar neurons for primary culture were prepared from post-natal day 2 (P2) *Slc38a6*^+/+^ and *Slc38a6*^-/-^ mouse as described previously.^2^ The cerebellum was dissociated and incubated in papain solution [calcium and magnesium free Hank’s balanced salt solution (CMF-HBSS)) containing 3 mg/mL papain (Sigma, P4762) and 40 μg/mL DNAse I (Sigma, DN25) at 37℃ for 30 min. The digestion solution was centrifuged at 300× g for 5 min at 4℃ and cerebellar tissues were washed once with CMF-HBSS to end digestion. The cerebellar tissues were gently triturated in CMF-HBSS with a Pasteur pipette to dissociate aggregates and filter cell suspension through 70 μm filter. The primary cerebellar neurons were plated on a dish coated with poly-D-lysine in DMEM/F12. Six hours after plating, media was replaced by DMEM/F12 containing 10% FBS supplemented with N3 supplement solution (200× stock): Progesterone 4 μM (Sigema, P8783), bovine insulin 2 mg/mL (Sigema, I6634), and transferrin 20 mg/mL (Sigema, C7786).

The wild-type *SLC38A6* and mutant plasmids were transfected into Hela cells for uptake assays with ^3^H labeled amino acid.^3^ The cells were seeded in 24-well plates and transfected with plasmids for 36 h. Media was then removed and incubated in Hank’s solution (in mM, 136.6 NaCl, 5.4 KCl, 2.7 Na_2_HPO_4_, 1.3 CaCl_2_, 0.5 MgCl_2_, 0.44 KH_2_PO_4_, 0.41 MgSO_4_, 5 HEPES, 5 Glucose) for 10 min prior to the uptake assays. Then, the cells were incubated in ^3^H labeled amino acid solution (1.6 μM unlabeled amino acid and 0.4 μM ^3^H labeled amino acid diluted in Hank’s solution) for 30 min. The cells were lysed in 200 μL 10% SDS after three washes with Hank’s solution to stop the uptake and transferred into scintillation tubes with 1 mL scintillation liquid. The mixture was measured as counts per min (cpm) with Quanta Smart Software in the Tri-Carb 4810TR liquid scintillation analyzer. The ^3^H labeled glutamine and arginine uptake in primary cerebellar neurons was performed as described above.

**Coat hanger task**

*Slc38a6^+/+^* and *Slc38a6^-/-^* mice were tested for the prehensile strength and coordination in the coat hanger test as previously reported.^4^ The mouse was suspended on the center of the horizontal portion of a coat hanger by its forepaws. The latency to fall was recorded over a maximum period of 60 s. The prehensile strength was evaluated by scoring the behavior on the coat hanger as it follows: 0 (mouse fell off the wire); 1 (mouse was able to hang or lift one of hind limbs); 2 (mouse lifted both of its hind limbs); 3 (mouse was able to move to the end of coat hanger).

**Balance beam task**

To evaluated the ability of *Slc38a6^+/+^* and *Slc38a6^-/-^* mice to maintain balance, the mice were placed on 2.5 cm-width balance beam and trained to pass from the starting point to the terminal box.^5^ On the test day, mice were placed on the beam 100 cm from the terminal box and allowed to cross 1.5 cm-width and 1 cm-width beam, respectively. Maximum trial length was 60 s and the latency to pass through the beam was recorded.

**Open field test**

*Slc38a6^+/+^* and *Slc38a6^-/-^* mice were tested for locomotor activity and anxiety in the open field test.^5^ Mice were allowed to move freely in an open field box (40×40×40 cm) for 10 min to analyze behavioral patterns, including time spent in the central zone and total distance traveled.

**Footprint test**

To analyze the gait of *Slc38a6^+/+^* and *Slc38a6^-/-^* mice, we dipped the paws of mice into non-toxic pigments (red and yellow were used for fore paws, blue and green were used for hind paws) and allowed the mice to run along a straight narrow tunnel (5×10×100 cm) with white paper to record the prints. Measurements for five steps were averaged and footprints at the start and the end of tunnel were excluded. The parameters registered were the front base width, hind base width, the forelimb stride length, hind-limb stride length, and the overlap between hind and forelimbs.^5^

**H&E staining and immunohistochemistry of paraffin cerebellar sections**

The 7 μm thick paraffin cerebellar sections were deparaffinized in xylene, and rehydrated sequentially in a series of ethanol solutions from 100% to 75%, followed by incubation with water for 5 min. The processed sections were stained with hematoxylin and eosin (H&E) and imaged using a microscope (Olympus BX53). The number and length of PC layer were counted across 10 selected fields at 200× magnification using the ImageJ software, as previously described. The PC linear density was calculated by dividing the number of PCs by the length of the PC layer.^6^

The paraffin cerebellar sections were deparaffinized and rehydrated, followed by antigen retrieval with citrate buffer (Beyotime, P0081, pH 6.0) for 20 min. The sections were blocked with 3% BSA and 1% Triton X-100 in PBS, and then incubated with rabbit anti-calbindin-D28K (1:1000, Swant, CB38) at 4℃ overnight. After several washes in 0.1% Tween20-PBS, the sections were incubated with goat anti-rabbit alkaline phosphatase secondary antibody (1:1000, Thermo, G21079) for 2 h at RT and developed by staining with Immpact Vector Red according to manufacturer’s instructions (Vector Laboratories, SK5105). Next, the sections were blocked with 3% H_2_O_2_ to eliminate endogenous peroxidase activity, and incubated with anti-GAD primary anti-body (MBL International Corp, M018-3, 1:300) overnight at 4℃. The sections were treated with Vectastain ABC kit containing biotinylated secondary antibody and biotinylated HRP. Finally, GAD staining was developed by 3,3’-diaminobenzidine chromagen solution (DAB) (Servicebio, G1212). The images were acquired using Olympus VS120 at 200× magnification. To quantify empty basket pathology, the plexus of basket cell axons around PC with and without detectable PC soma represented “full basket” and “empty basket”, respectively.^7^ The number of full and empty baskets were counted across the all cerebellar lobules. For each cerebellar sections, the number of empty baskets divided by the total number of full and empty baskets represented the percentage of empty baskets, indirectly expressed as the PC soma loss.

Paraffin cerebellar sections were deparaffinized, rehydrated, antigen retrieved and blocked as above. The sections were incubated with TFRC (1:1000, Invitrogen, 136800) overnight at 4℃ and treated with secondary antibody and biotinylated HRP. Finally, the sections were incubated with DAB and hematoxylin for 3 min.

**VGluT2 immunohistochemistry of paraffin cerebellar sections**

To visualize the CF‒PC synapses,^8^ 7 μm thick paraffin cerebellar sections were stained for vGluT2, a specific marker of CF terminals. The sections were processed as above and incubated with rabbit anti-vGluT2 antibody (Synaptic Systems, 135403, 1:250) at 4°C for 48 h. Then, the sections were incubated with biotin-SP goat-anti-rabbit antibody (Thermo Fisher Scientific, 31820, 1:200) at RT for 2 h prior to streptavidin-horseradish peroxidase (Bio-Rad, STAR5B, 1:200) for 1 h to amplify the signals. Finally, sections were developed with 3,3’-diaminobenzidine chromagen solution (Servicebio, G1212). The images were acquired with bright field microscopy (Olympus BX53).

The method to assess CF synaptic density was performed as previously described.^8^ The total number of vGluT2^+^ puncta on PC dendrites and total length of CF were measured using Image J software across 10 randomly selected regions on all cerebellar lobules at 400× magnification. The total number of vGluT2^+^ puncta divided by total length of CF was defined as the CF synaptic density. The molecular layer was measured by trained raters and dotted-line was drawn with Image J software. The total number of vGluT2 puncta in outer 20% of the molecular layer was counted across 5 randomly selected fields at 400× magnification.

**Bieschowsky staining of paraffin cerebellar sections**

A semiquantitative scoring of the morphology of the basket cell plexus surrounding PC soma throughout Bielschowsky stained sections was conducted by a trained rater, who was blind to mouse genotypes. The semiquantitative method was performed as previously described, with some modification.^9^ To facilitate the assessment of the degree of alteration, we classified hairy basket scoring into three categories as follows: low (1, minimum number of axonal collaterals,); intermediate (2, moderate number of axonal collaterals); high (3, numerous axonal collaterals). The trained rater assessed ten randomly-selected fields for each mouse cerebellar section, and then scored the dense extent of basket cell plexus throughout the entire cerebellum in the selected field. This was done in *Slc38a6^+^*^/+^ and *Slc38a6*^-/-^ mice at 2, 4, 6, 9 and 12.5 months (*Slc38a6^+^*^/+^ = 6; *Slc38a6*^-/-^ = 6, per age group). For each genotype and age, significant differences were determined by two-way ANOVA.

The quantitative analysis of basket cell plexus was applied following the protocol of the quantitative analysis of postmortem brains of ET patients with some modifications. The rating criteria of basket cell plexus was used as follows: 0 (no detectable processes); 1 (thin processes); 2 (moderate processes); 3 (dense twining processes). For further study, the number of basket cell plexus at disparate grade (from 1 to 3) was counted and averaged across the same ten randomly selected fields. This was done in *Slc38a6^+^*^/+^ and *Slc38a6*^-/-^ mice at 2, 4, 6, 9 and 12.5 months (*Slc38a6^+^*^/+^ = 6; *Slc38a6*^-/-^ = 6, per age group). The mean number of the basket cell plexus was not normally distributed. Hence, these were compared across groups using nonparametric statistics: Mann-Whitney U tests.

**Immunofluorescence and immunohistochemistry of frozen cerebellar Sections**

The 8 μm thick frozen sections were heated with citrate buffer (Beyotime, P0081, pH 6.0) in steamer for 10 min to retrieve the antigen and blocked with 0.1% Triton X-100 and 3% BSA in PBS for 30 min. The sections were incubated overnight at 4℃ with rabbit anti-SNAT6 (1:200, Abcam, ab121572). After three washes in PBS, the sections were incubated with secondary antibody Alexa Fluor 594 (1:500, Invitrogen, A-11012) for 1 h at RT. Images were captured using a fluorescence microscope (FV1000, Olympus, Japan).

Free-floating 100 μm thick cerebellar frozen sections were incubated with 20 µg/µl Proteinase K (Sigma Aldrich, 03115828001) at 37°C for 15 min and treated with 3% hydrogen peroxide for 30 min to eliminate endogenous peroxidase. After blocking the sections with 10% BSA and 1% TritonX-100 in PBS, rabbit anti-calbindin-D28K (Swant, CB38, 1:1000) was applied and incubated at 4℃ overnight followed by incubation with Biotin-SP goat-anti-rabbit antibody (Thermo Fisher Scientific, 31820, 1:200) for 2 h at RT. Then, the sections were incubated with streptavidin-horseradish peroxidase (Bio-Rad, STAR5B, 1:200) for 1 h at RT, and developed with 3,3’-diaminobenzidine chromagen solution (Servicebio, G1212). The sections were dehydrated in ethyl alcohol and cleared in xylene, and finally images were obtained using bright field microscopy (Olympus BX53).

The PC axonal features, including thickened axonal profiles (axons display a width that is at least twice the width of other apparently normal axons), torpedoes (ovoid axonal swellings) and recurrent axonal collaterals (with at least a 90° turn back towards the PC layer from their initial trajectory), were counted across 10 randomly selected regions for each cerebellar section. The length of PC layer with a visible recurrent collateral plexus was calculated by the raw counts of PC axonal features normalized to the total length of PC layer length and displayed as a percentage of total PC layer length.

**Golgi staining of cerebellar tissue**

The cerebellar tissue block was stained with Golgi-cox as previously described.^10^ Sagittal sections were acquired with a cryotome at 100 μm thickness to increase the number of PCs with complete branching of their dendrites. 20 isolated PCs per block were chosen for analysis based on published criteria: (i) soma located at a medium depth of the section to minimize cutting-off of distal dendrite branches; (ii) dark homogeneous impregnation throughout the extent of neuronal dendrites, indicating adequacy of Golgi staining; (iii) neurons were only partially covered by adjacent neuronal structures; (iv) distal branches exhibited natural terminations. The Sholl’ s method of concentric circles was adopted for PC dendrites analysis with a plugin of Image J.

**Slice electrophysiology**

In brief, 2 month-old mice were anesthetized with isoflurane and decapitated. Their brains were then rapidly removed and cooled in ice-cold low-Ca^2+^ artificial cerebrospinal fluid (ACSF) containing (in mM): 125 NaCl, 2.5 KCl, 3 MgCl_2_, 0.1 CaCl_2_, 25 glucose, 1.25 NaH_2_PO_4_, 0.4 ascorbic acid, 3 myo-inositol, 2 Na-pyruvate, and 25 NaHCO_3_, oxygenated with a mixture of 95% O_2_ and 5% CO_2_. Sagittal slices (250 μm) of the cerebellum were prepared using a VT1200 vibratome (Leica) and transferred to ACSF containing (in mM) 1 MgCl_2_ and 2 CaCl_2_ instead of 3 MgCl_2_ and 0.1 CaCl_2_. Slices were allowed to recover at 32℃ for 30 min, and equilibrated at RT for another hour before being transferred to the recording chamber. Slices were immobilized in a submerged chamber and perfused continuously with oxygenated ACSF at 35℃. Cerebellar PCs were visualized using an infrared differential interference contrast (IR-DIC) microscope (BX51WI, Olympus, Japan) equipped with a water immersion objective (40×, NA 0.80).

Whole-cell recordings from PCs in cerebellar lobules IV-VI were performed with patch pipettes (4-6 MΩ). To compare the neuronal excitability of PCs, cerebellar PCs were recorded using a K-gluconate based internal solution containing (in mM): 140 K-gluconate, 5 KCl, 2 MgCl_2_, 10 HEPES, 0.5 EGTA, 2 Na-ATP, and 0.5 Na-GTP (pH 7.3, adjusted with KOH). Briefly, cerebellar PCs were held at ~ −70 mV in current clamp mode, and 600 ms of step depolarization (from 0 to 800 pA in 50 pA increments) were injected into the recorded PCs through the recording pipettes. To record the spontaneous inhibitory postsynaptic currents (sIPSCs), cerebellar PCs were recorded with a holding potential of 0 mV using a CsMeSO_3_-based internal solution containing (in mM): 130 CsMeSO_3_, 1 MgCl_2_, 1 CaCl_2,_ 10 HEPES, 2 QX-314, 11 EGTA, 2 Mg-ATP and 0.3 Na-GTP. For sIPSC recordings, the aCSF bath solution contained 10 µM (NBQX), 10 µM (D-APV) to block excitatory input. Signals were digitized at 20 kHz and filtered at 4 kHz using Clampex v10.4 (Molecular Devices). The frequency and amplitude of sIPSCs were further analyzed by the Mini-anlysis as described previously.^11^ Neurons were discarded if the access resistance was greater than 20 MΩ, or if the access resistance increased by 20% during recording.

**Luciferase reporter and Chromatin immunoprecipitation (ChIP) assays**

For reporter assays, M17 cells were co-transfected with pGL4.10-hpSLC38A6 and pcDNA3.1-hATF3 or pcDNA3.1, followed by Renilla plasmid. The reporter activities were measured following the manufacturer’s protocol.

ChIPs were performed by SimpleChIP Plus Enzymatic Chromatin IP Kit (Magnetic Beads) (CST) following the manufacturer’s protocol.

**Supplementary Notes**

**Identification of *SLC38A6* variants in families with ET**

We analyzed the distribution of *SLC38A6* variants in our case-control samples and we found that the frequency of ET cases carrying C.842T >C (p.M281T) and C.952G>A (p.G318S) variants was higher than that of normal controls (Supplementary Table 4). What draws our attention is that there were significant differences in the frequency of *SLC38A6* variants identified in this cohort between East Asian and European populations. The fifteen *SLC38A6* variants identified in our ET cohort are rarely observed in the European population (Supplementary Table 4). This suggests that *SLC38A6* may predominantly play a role in the East Asian population.

**Behavioral and morphological abnormalities in *Slc38a6^+/-^* mice**

Since almost all patients in our cohort carry a heterozygous mutation of *SLC38A6*, we performed behavioral and pathological analyses of heterozygous *Slc38a6* KO mice (*Slc38a6*^+/-^). We observed that the *Slc38a6^+/-^* mice showed ET-like tremor and the tremor as severe as *Slc38a6^-/-^* mice at later stage (Supplementary video 8 and 9). The *Slc38a6^+/-^* mice showed significant PC loss (9 months: *Slc38a6*^+/+^:30.4 ± 0.6, *Slc38a6*^+/-^:27.5 ± 0.8, *p* = 0.0343; 12.5 months: *Slc38a6*^+/+^:31.1 ± 0.8, *Slc38a6*^+/-^:25.4 ± 1.0, *p* = 0.0014) (Supplementary Fig. 10a and b), abnormal PC axon (Supplementary Fig. 10c) and “hairy” basket cell axon (Supplementary Fig. 10d). We also found that there are reduced CF‒PC synaptic density (9 months: *Slc38a6*^+/+^:24.1 ± 0.4, *Slc38a6*^+/-^:22.6 ± 0.4, *p* = 0.0423; 12.5 months: *Slc38a6*^+/+^:24.2 ± 0.3, *Slc38a6*^+/-^:20.0 ± 0.6, *p* < 0.0001) (Supplementary Fig. 10f) and increased CF‒PC synapses in the outer 20% of molecular layer (9 months: *Slc38a6*^+/+^:69.2 ± 4.2, *Slc38a6*^+/-^:116.3 ± 9.5, *p* = 0.0008; 12.5 months: *Slc38a6*^+/+^:62.0 ± 6.1, *Slc38a6*^+/-^:149.3 ± 11.1, *p* < 0.0001) in the *Slc38a6^+/-^* mice. Besides, we found that the *Slc38a6*^+/-^ mice at 6 months of age showing reduced excitability in PC (Supplementary Fig. 10h and i). Elevated levels of TFRC and ATF3 in *Slc38a6*^+/-^ cerebellum were also found (Supplementary Fig. 10j and k). Overall, *Slc38a6*^+/-^ mice showed significant morphological, electrophysiological or molecular alterations similar to *Slc38a6*^-/-^ mice. Overall, *Slc38a6*^+/-^ mice showed significant morphological, electrophysiological or molecular alterations similar to *Slc38a6*^-/-^ mice.

ATF3 as a transcription factor was predicted to bind to the promoter of *SLC38A6* based on JASPAR databas, and we also verified that ATF3 bound strongly to the *SLC38A6* promoter at two sites by using ChIP assays (Supplementary Fig. 10l). Upregulated ATF3 in *Slc38a6*^+/-^ mice further inhibited *SLC38A6* expression (Revised Supplementary Fig. 10m). Therefore, we suppose a dominant negative effect in the *Slc38a6*^+/-^ mice, possibly due to up-regulated expression of ATF3.

**REFERENCES**

1 Sun, Q. Y. *et al.* Expansion of GGC repeat in the human-specific NOTCH2NLC gene is associated with essential tremor. *Brain* **143**, 222-233 (2020).

2 Giordano, G. & Costa, L. G. Primary neurons in culture and neuronal cell lines for in vitro neurotoxicological studies. *Methods. Mole. Biol. (Clifton, N.J.)* **758**, 13-27, (2011).

3 Sundberg, B. E. *et al.* The evolutionary history and tissue mapping of amino acid transporters belonging to solute carrier families SLC32, SLC36, and SLC38. *J. Mole. Neurosci.* **35**, 179-193, (2008).

4 Bárez-López, S. *et al.* Abnormal motor phenotype at adult stages in mice lacking type 2 deiodinase. *PLoS One* **9**, e103857, (2014).

5 Jang, H. *et al.* Gait Ignition Failure in JNPL3 Human Tau-mutant Mice. *Experimental neurobiology* **28**, 404-413, (2019).

6 Louis, E. D. *et al.* Neuropathological changes in essential tremor: 33 cases compared with 21 controls. *Brain* **130**, 3297-3307, (2007).

7 Lee, P. J. *et al.* A quantitative study of empty baskets in essential tremor and other motor neurodegenerative diseases. *J. Neuropathol. Exp. Neurol.* **78**, 113-122, (2019).

8 Kuo, S. H. *et al.* Climbing fiber-Purkinje cell synaptic pathology in tremor and cerebellar degenerative diseases. *Acta. Neuropathol.* **133**, 121-138, (2017).

9 Erickson-Davis, C. R. *et al.* "Hairy baskets" associated with degenerative Purkinje cell changes in essential tremor. *J. Neuropathol. Exp. Neuro.l* **69**, 262-271, (2010).

10 Koyama, Y. & Tohyama, M. A modified and highly sensitive Golgi-Cox method to enable complete and stable impregnation of embryonic neurons. *J. Neurosci. Methods.* **209**, 58-61, (2012).

11 Zhang, B. *et al.* Neuroligins Sculpt Cerebellar Purkinje-Cell Circuits by Differential Control of Distinct Classes of Synapses. *Neuron* **87**, 781-796, (2015).


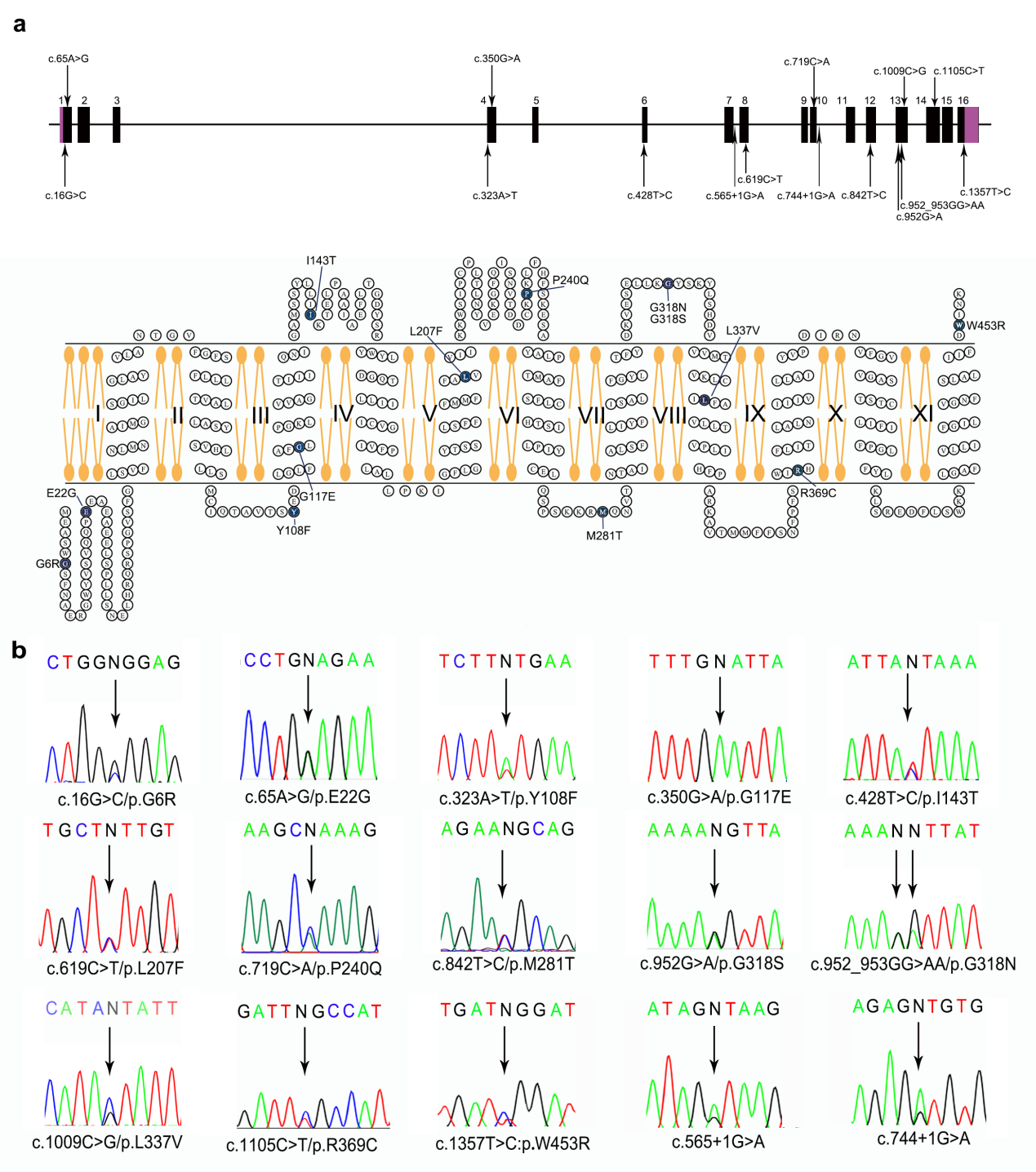
**Supplementary Fig. 1. 15 types of *SLC38A6* variants identified in the proband of families and sporadic patients with ET.** (**a**) Schematic display of *SLC38A6* with ET-associated variants indicated. (Upper) Schematic structure diagrams of SNAT6 with ET-associated mutations (blue circles)(down). The transmembrane domains are numbered from I to XI. The structure model was drawn using the TOPO2 software (http://www.sacs.ucsf.edu/TOPO/top.html). (**b**) Representative images of Sanger sequencing on *SLC38A6* variants and the variants are marked by arrows.


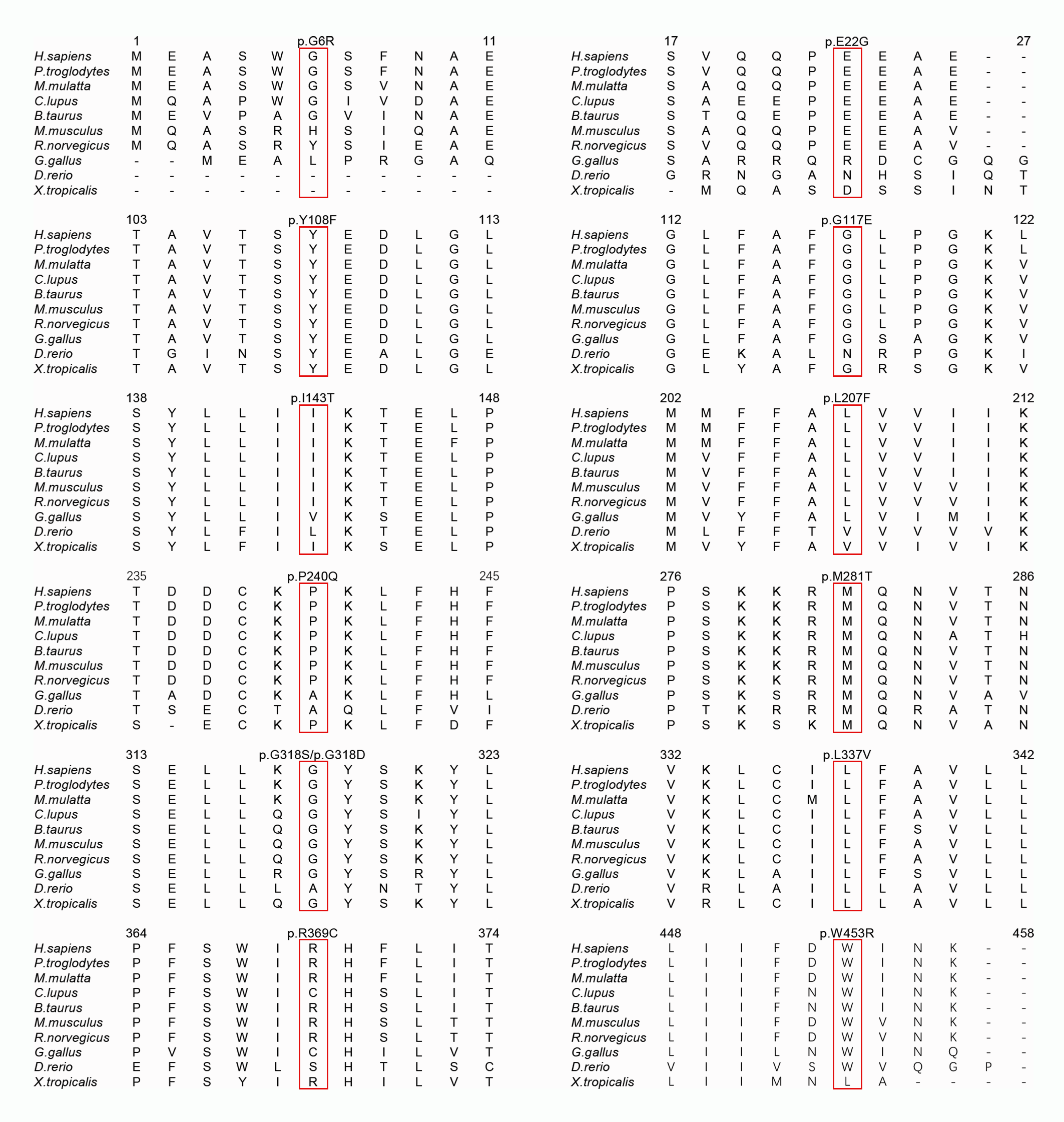


**Supplementary Fig. 2. Evolutionary conservation analysis for amino acids of SNAT6 variants in different species.** The HomoloGene (http://www.ncbi.nlm.nih.gov/homologene) was used to evaluate the evolutionary conservation of amino acids affected by the 13 missense variants. The locations of the altered amino acids are marked by red boxes.


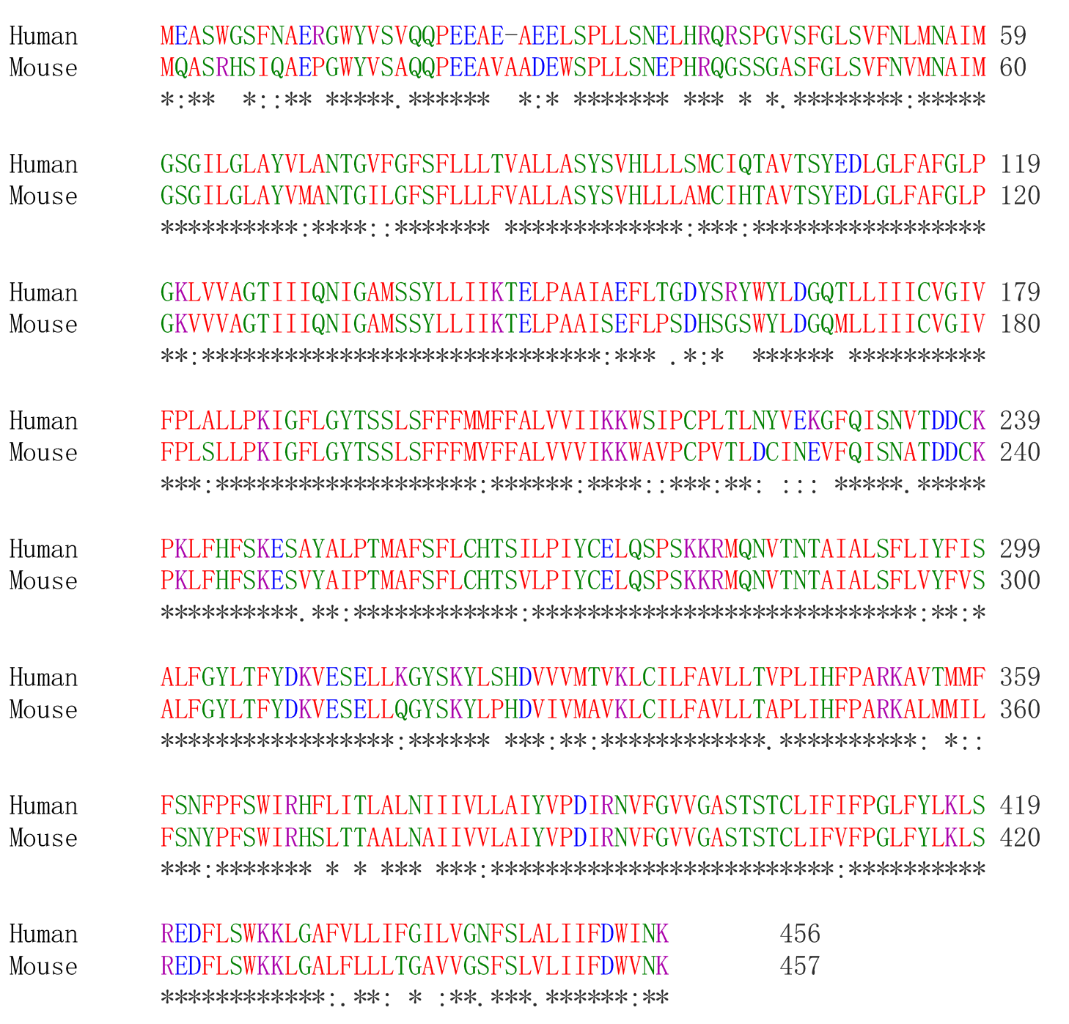


**Supplementary Fig. 3. The alignment of amino acids for human and mouse SNAT6.** The Clustal Omega (http://lilab2.sysu.edu.cn/Tools/msa/clustalo/) was used to evaluate the amino acid identity for human and mouse SNAT6. ‘*’ represents identical amino acid residues. ‘.’ and ‘:’ represent conserved amino acid sequences with different degrees of conservation. ‘-’ represents deletion of amino acid at this position. No identifier indicates that the sequence does not show conservation.


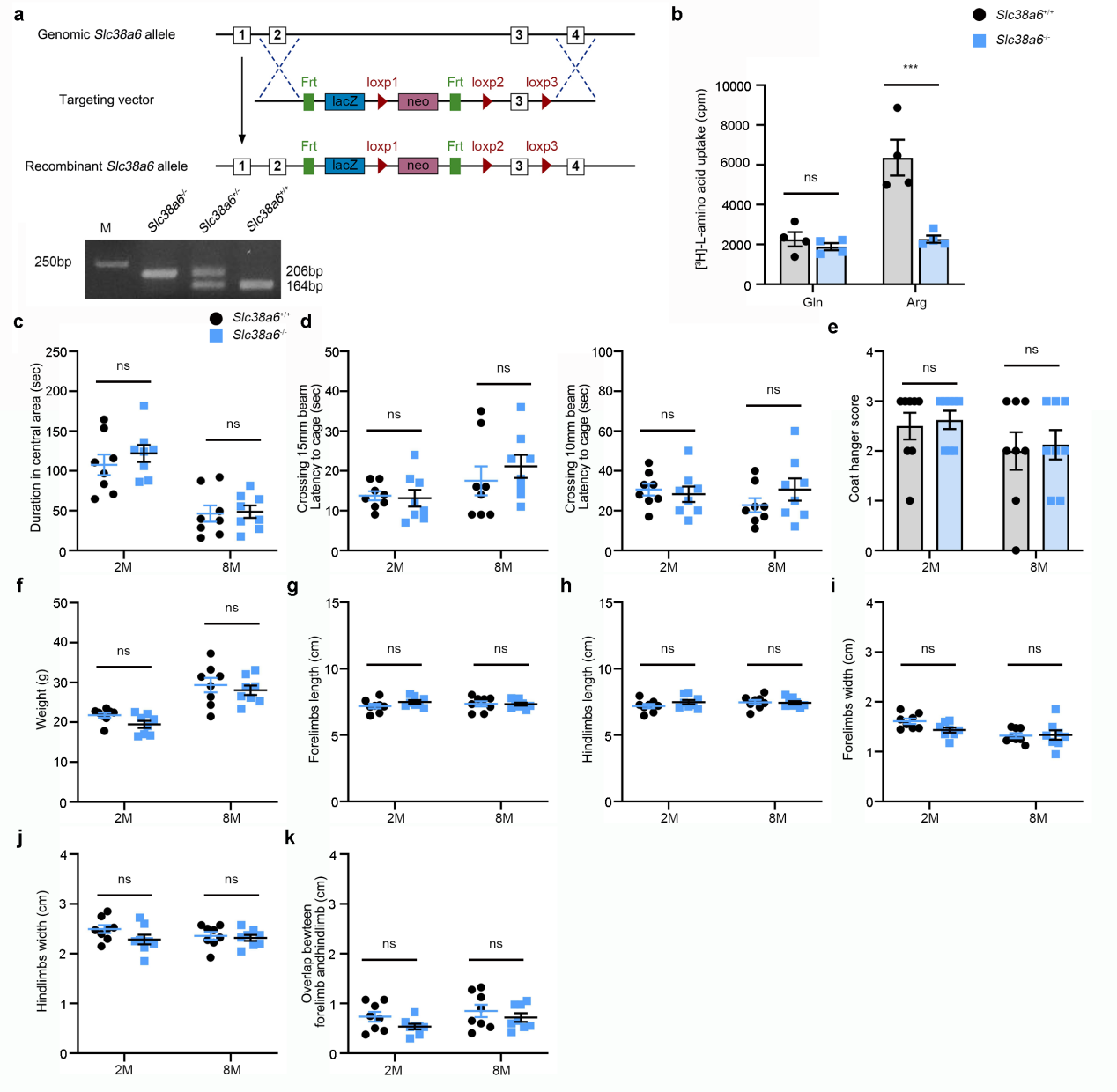


**Supplementary Fig. 4. *Slc38a6* deletion in mice impaired uptake of L-Arg but not cognitive and motor function.** (**a**) Schematic of the *Slc38a6* deletion mice with ‘knockout-first’ strategy. The 'knockout-first' allele disrupts the function of the *Slc38a6* by inserting a lacZ and neo reporter cassette flanked by Frt sites into the intron. PCR genotyping results of *Slc38a6*^-/-^ (206 bp) alleles (left), *Slc38a6*^+/-^ (206 bp and 164 bp) alleles (middle) and *Slc38a6*^+/+^ (164 bp) alleles (right). (**b**) ^3^H-labeled glutamine and arginine uptake in *Slc38a6*^+/+^ and *Slc38a6^-^*^/-^ primary cerebellar neuron was measured in counts per minute (cpm) using scintillation counting. (**c**) Duration of *Slc38a6*^+/+^ and *Slc38a6*^-/-^ mice at 2 and 8 months of age in central area of the open field test (n = 8 mice in each group). (**d**) Latency of *Slc38a6*^+/+^ and *Slc38a6*^-/-^ mice at 2 and 8 months of age spent to cross the 15mm and 10mm balance beam (n = 8 mice in each group). (**e**) Coat hanger test scores for *Slc38a6*^+/+^ and *Slc38a6*^-/-^ mice at 2 and 8 months of age to evaluate coordination (n = 8 mice in each group). (**f**) Weight of *Slc38a6*^+/+^ and *Slc38a6*^-/-^ mice at 2 and 8 months of age (n = 8 mice in each group). (**g‒k**) Gait analysis for *Slc38a6*^+/+^ and *Slc38a6*^-/-^ mice at 2 and 8 months of age, including forelimbs stride length (g), hindlimbs stride length (h), front base width (i), hind base width (j) and overlap between fore and hindlimbs (k). The data are presented as the means ± SEMs. Statistical tests: two-tailed unpaired Student’s *t* test (b), two-way ANOVA followed by Sidak's multiple comparisons tests (c**‒**k).


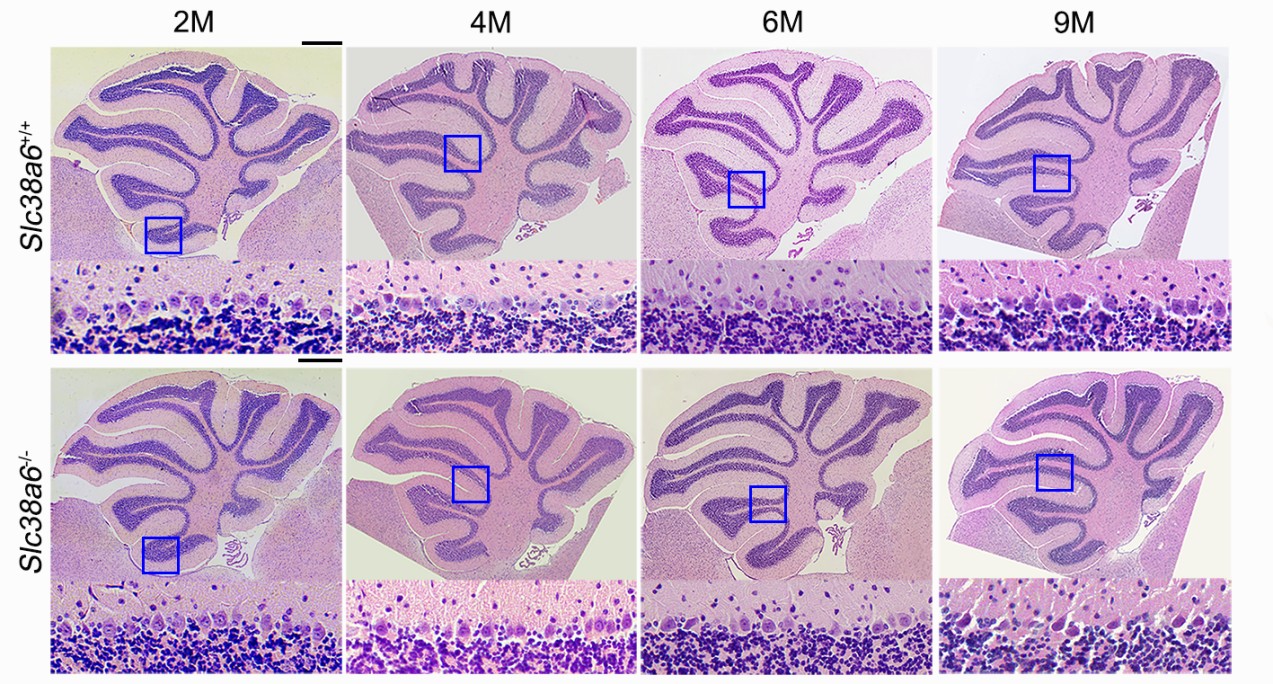


**Supplementary Fig. 5. The alterations of PC linear density in the cerebellum of *Slc38a6^+/+^* and *Slc38a6^-/-^* mice.** Representative cerebellar sagittal sections of *Slc38a6*^+/+^ and *Slc38a6*^-/-^ mice at 2, 4, 6 and 9 months of age stained with H&E. Scale bars: 500 μm (upper), 50 μm (bottom).

**
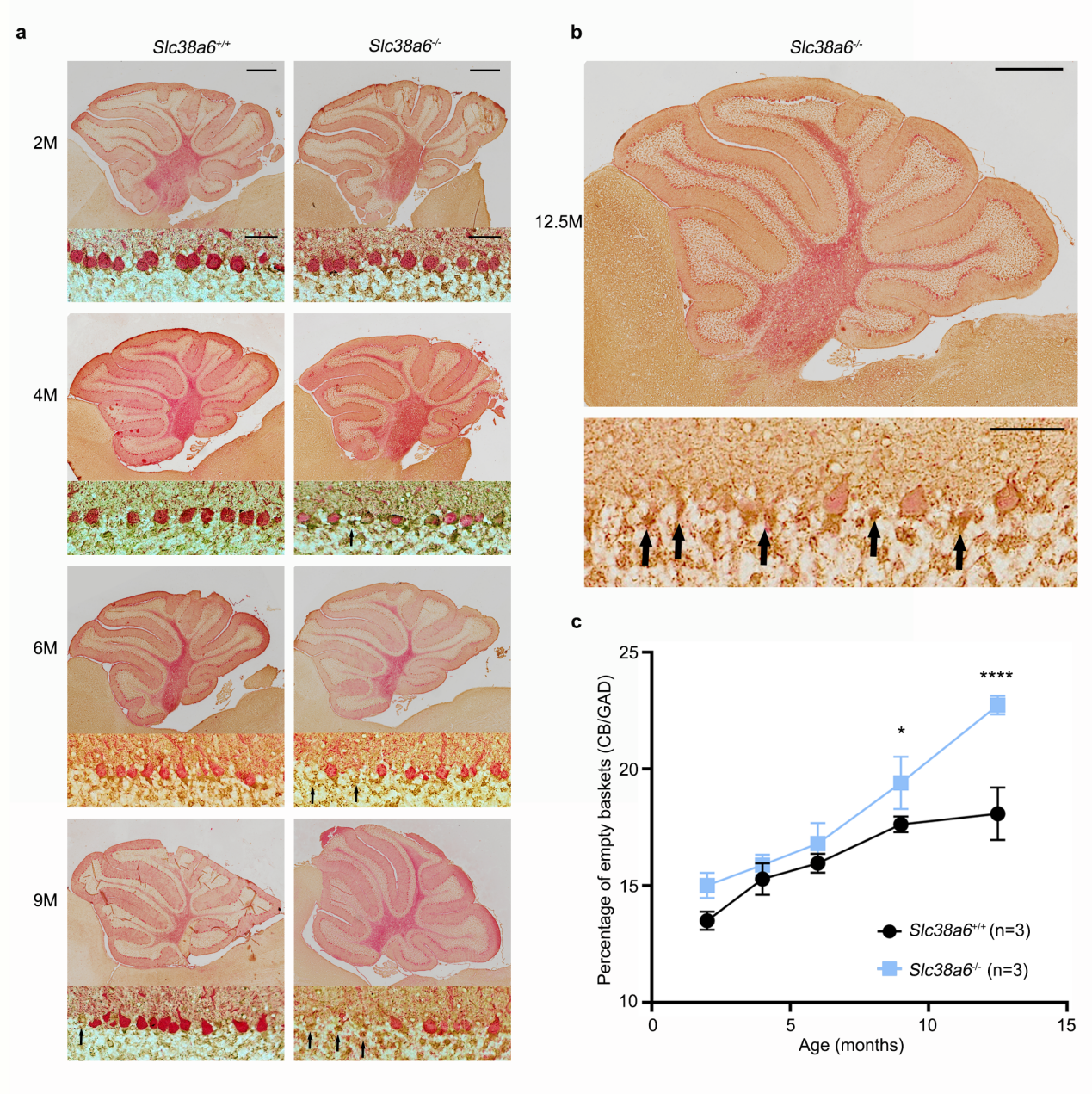
**

**Supplementary Fig. 6. The quantification of “empty basket” from *Slc38a6^+^*^/+^ and *Slc38a6*^-/-^ mice.** (**a‒b**) Top: representative 7 μm paraffin sagittal cerebellar sections of *Slc38a6^+^*^/+^ and *Slc38a6*^-/-^ mice at 2, 4, 6, 9, and 12.5 months of age, immunostained with calbindin-D28K and glutamic acid decarboxylase (GAD). Bottom: images demonstrate higher magnification of the sections shown in the top panel. GAD-labeled pinceau (brown) exhibit a triangular shape at the base of the PC soma (red). Black arrows indicate “empty baskets”, with a visible portion of the pinceau and remaining basket processes at the junction of the molecular layer and granule layer without any detectable PC soma. (**c**) The percentage of the empty basket in *Slc38a6^+^*^/+^ and *Slc38a6*^-/-^ mice from 2 to 12.5 month-old (*Slc38a6*^+/+^ vs. *Slc38a6*^-/-^: 9 month-old: *p* = 0.0256; 12.5 month-old: *p*< 0.0001, n = 3 mice in each group). The data are presented as the means ± SEMs. Statistical tests: two-way ANOVA followed by Sidak's multiple comparisons tests (c). Scale bars: 500 μm (upper of a, b); 50 μm (bottom of a, b).

**
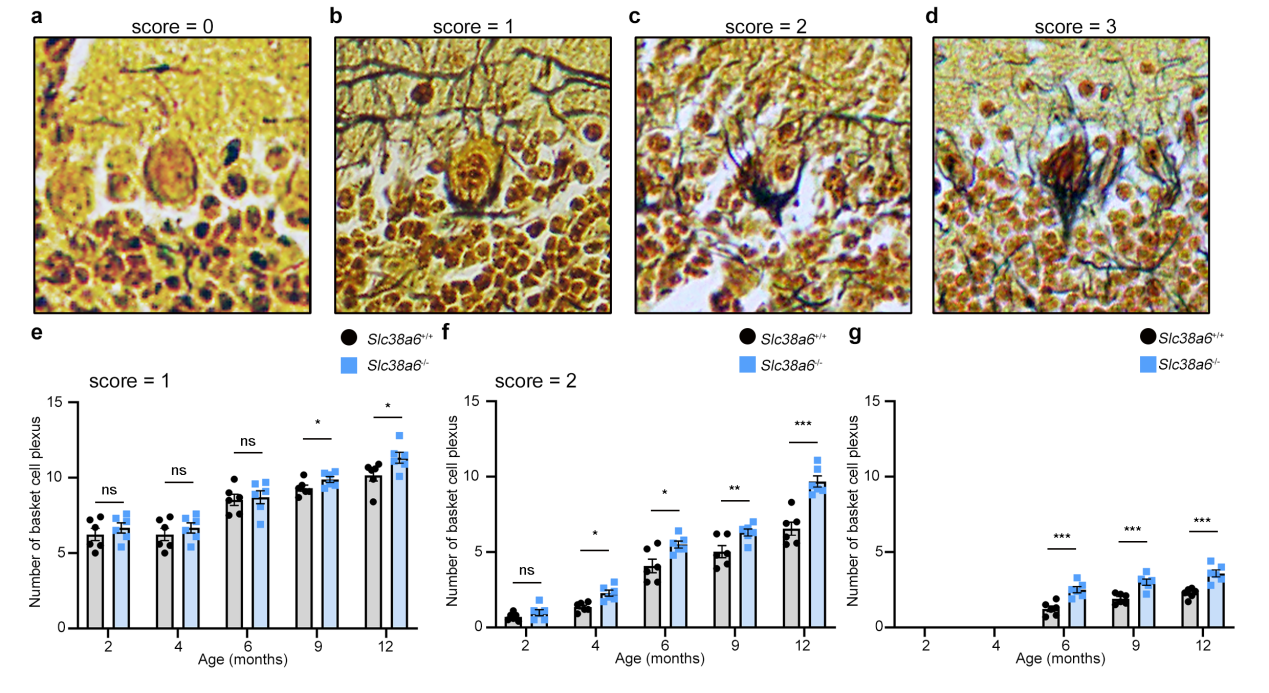
**

**Supplementary Fig. 7. Quantitative analysis of hyperplastic basket cell axonal collaterals (“hairy basket”).** (**a‒d**) “Hairy baskets” defined as a dense and twining axonal plexus generally surrounding detectable PCs with age. Hairy basket rating of the appearance of the basket cell plexus surrounding extant PC soma was performed in Bielschowsky silver-stained mouse cerebellar sagittal sections (from 0 to 3). (a) Score = 0 (no visible processes); (b) Score = 1 (a few processes, slight plexus alteration); (c) Score = 2 (sparse number of processes, mild plexus alteration); (c) Score = 3 (dense twining of processes, severe plexus alteration). (**e‒g**) The number of basket cell plexus was quantified in *Slc38a6^+^*^/+^ and *Slc38a6*^-/-^ mouse sections at 2, 4, 6, 9, and 12.5 months of age (n = 6 mice each group). The number of basket cell axonal collaterals across 10 randomly selected microscopic fields at 200× magnification was counted and averaged. The data are presented as the means ± SEMs. Statistical tests: Mann**‒**Whitney tests (e**‒**g).

**
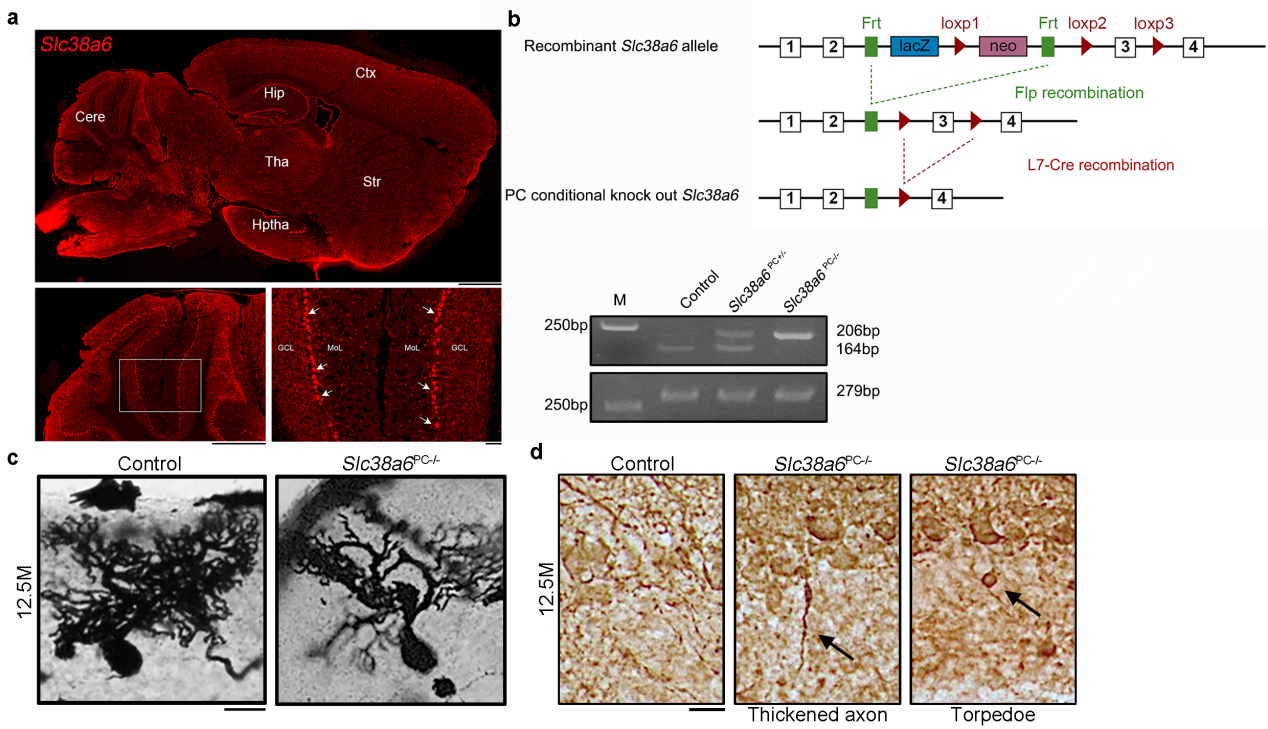
Supplementary Fig. 8. The generation of *Slc38a6*^PC-/-^ mice and pathological changes in control and *Slc38a6*^PC-/-^ mice.** (**a**) Representative sagittal section of IHC staining of *Slc38a6* in wild-type mice. The bottom images are higher magnifications of the cerebellum section in the white rectangle. (**b**) Design of the targeting vector and strategy for generation of *Slc38a6*^PC^*^-/-^* mice using flp transgenic mice and L7-Cre transgenic mice. PCR genotyping results of *Slc38a6* and Cre (279bp). (**c**) Representative PC dendrites of Golgi stained sections from 12.5 month-old control and *Slc38a6*^PC^*^-/-^*, respectively. (**d**) Representative abnormal PC axons using calbindin-D28K IHC staining in *Slc38a6*^PC-/-^ mice at 12.5 months of age. Thickened axon and torpedo were shown (arrow heads). Scale bars: 1 mm (upper of a); 500 μm (bottom left of a); 50 μm (bottom right of a); 20 μm (c, d).

**
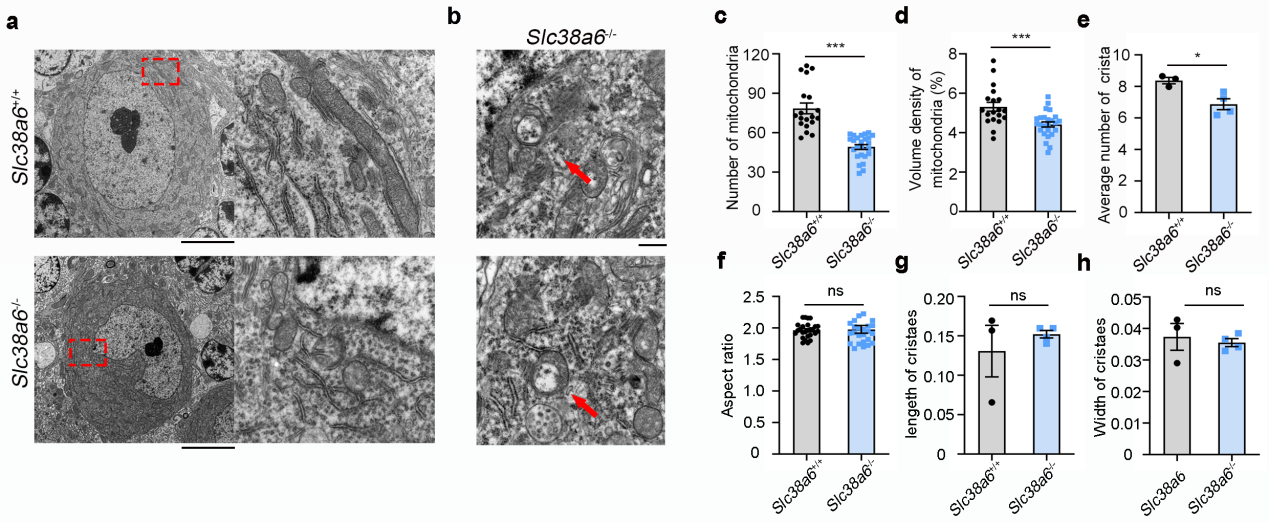
**

**Supplementary Fig. 9. TEM analysis in 2 month-old *Slc38a6^+/+^* and *Slc38a6^-/-^* mice.** (**a**) Representative Perls’ Prussian blue staining of *Slc38a6*^+/+^ and *Slc38a6*^-/-^ cerebellum. (**b**) Representative TEM images of mitochondrial elongation resulting in end-to-end connection in *Slc38a6*^-/-^ PCs. (**c**‒**e**) Number (c), volume density (d) and number of cristae (e) of mitochondria are decreased in *Slc38a6*^-/-^ mice. (**f**‒**h**) Aspect ratio of mitochondria (f), as well as length (g) and width (h) of mitochondrial cristae do not show any alteration. The data are presented as the means ± SEMs. Statistical tests: two-tailed unpaired Student’s *t*-test (c‒h). Scale bars: 10μm (a), 0.5 μm (b).


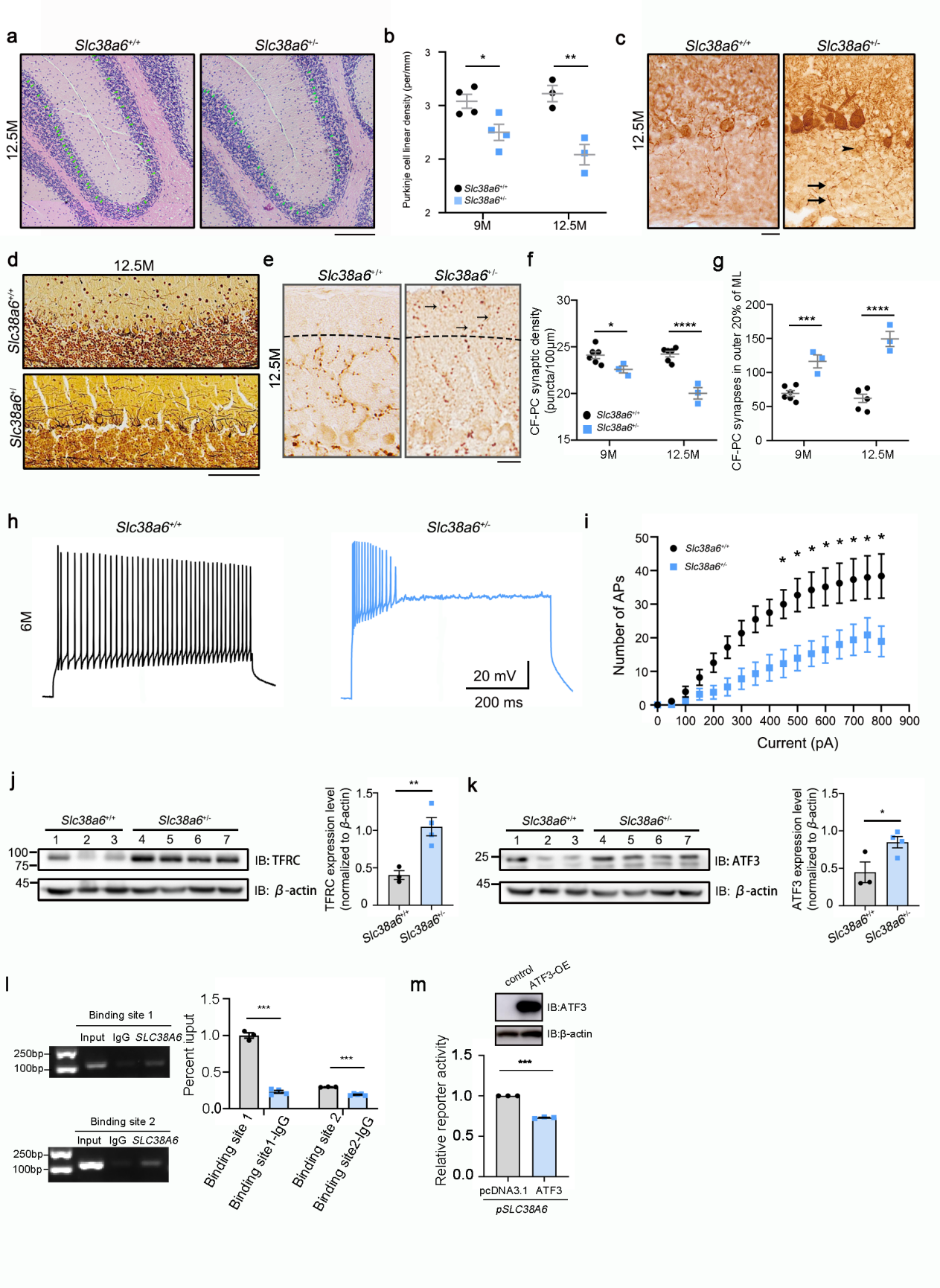


**Supplementary Fig. 10. *Slc38a6*^+/-^ mice show morphological, electrophysiological and molecular defects**

1. Representative H&E-stained cerebellar sagittal sections of *Slc38a6*^+/+^ and *Slc38a6*^+/-^ mice at 12.5 months of age. **(b)** Quantification of PC linear density of *Slc38a6*^+/+^ and *Slc38a6*^+/-^ mice at 9 and 12.5 months of age. **(c)** Representative PC axons stained with calbindin-D28K. Abnormal alterations of PC axons in *Slc38a6*^+/-^ mice at 12.5 months of age. One torpedo (arrowheads) and branched axons (arrows). **(d**) Representative “hairy baskets” of Bielschowsky silver-stained sections in *Slc38a6*^+/+^ and *Slc38a6*^+/-^ mice at 12.5 months of age. **(e)** Representative CF‒PC synapses in *Slc38a6*^+/+^ and *Slc38a6*^+/-^ mice at 12.5 months of age. Dotted line indicates the boundary of the outer 20% and inner 80% of molecular layer. Arrows dictate the CF‒PC synapses extending into the PF territory. **(f)** Quantitative statistics of CF synaptic density in *Slc38a6*^+/+^ and *Slc38a6^+^*^/-^ mice at 9 and 12.5 months of age. **(g)** Quantitative statistics of CF‒PC synapses in the outer 20% of the molecular layer in *Slc38a6*^+/+^ and *Slc38a6*^+/-^ mice at 9 and 12.5 months of age. **(h)** Representative APs of *Slc38a6^+/+^* and *Slc38a6^+/-^* PC evoked by current steps of 600 pA. **(i)** Number of APs induced by the current steps in *Slc38a6^+/+^* (n = 22, black) and *Slc38a6^+/-^* (n = 20, blue) PCs. **(j**) The expression levels of TFRC in *Slc38a6*^+/+^ and *Slc38a6^+/-^* cerebellum. (**k**) The expression levels of ATF3 in *Slc38a6*^+/+^ and *Slc38a6^+/-^* cerebellum. **(l)** ChIP assays confirm that ATF3 binds strongly to the human *SLC38A6* promoter. **(m)** Dual luciferase assays suggest that *SLC38A6* expression was repressed by ATF3 in M17 cells. Data are represented as mean ± SEM; Statistical tests: two-way ANOVA followed by Sidak's multiple comparisons tests (b) (f) (g); two-tailed unpaired Student’s *t*-test (j‒m). Scale bars: 200μm (a); 20μm (c)(e); 100μm(d).

**Supplementary Table 1. Clinical feature of family 1**

|  | | **V:6** | **III:5** | **III:13** | **IV:4** | **IV:6** | **IV:8** | **IV:10** | **IV:17** | **V:4** | **V:5** | **V:9** | **V:10** | **V:11** |
| --- | --- | --- | --- | --- | --- | --- | --- | --- | --- | --- | --- | --- | --- | --- |
| Variants | | c.842T>C/p.M281T; c.952G>A/p.G318S | | | | | | | | | | | | |
| Mutation type | | het | het | het | het | het | het | hom | het | het | het | het | het | het |
| Age (years) | | 37 | 81 | 78 | 60 | 65 | 57 | 55 | 50 | 40 | 41 | 38 | 33 | 28 |
| Sex – female | | F | F | M | M | F | M | F | M | M | F | M | M | M |
| Age of onset (years) | | 20 | 30 | 35 | 15 | 30 | 10 | 35 | 40 | 18 | 33 | 11 | 16 | 9 |
| Duration (years) | | 17 | 51 | 43 | 45 | 35 | 47 | 20 | 10 | 22 | 8 | 27 | 17 | 19 |
| Tremor | Upper limbs | **+** | **+** | **+** | **+** | **+** | **+** | **+** | **+** | **+** | **+** | **+** | **+** | **+** |
|  | Head | **-** | **+** | **+** | **+** | **-** | **-** | **-** | **-** | **-** | **-** | **-** | **-** | **-** |
|  | Voice | **-** | **+** | **+** | **+** | **-** | **-** | **-** | **-** | **-** | **-** | **-** | **-** | **-** |
|  | Lower limbs | **-** | **+** | **-** | **-** | **-** | **-** | **-** | **-** | **-** | **-** | **-** | **-** | **-** |
|  | Trunk | **-** | **+** | **-** | **-** | **-** | **-** | **-** | **-** | **-** | **-** | **-** | **-** | **-** |
| Hypermyotonia | | **-** | **-** | **-** | **-** | **-** | **-** | **-** | **-** | **-** | **-** | **-** | - | - |
| Pyramidal signs | | **-** | **-** | **-** | **-** | **-** | **-** | **-** | **-** | **-** | **-** | **-** | - |  |
| Ataxia signs | | **-** | **-** | **-** | **-** | **-** | **-** | **-** | **-** | **-** | **-** | **-** | **-** |  |
| TETRAS-Ⅰ | | 11 | 16 | 7 | 17 | 14 | 3 | 13 | 8 | 12 | 2 | 5 | 2 | 11 |
| TETRAS-Ⅱ | | 15.5 | 27 | 14 | 22 | 17.5 | 13 | 16 | 13 | 11.5 | 8 | 10 | 9 | 15.5 |
| MMSE | | 30 | 26 | 26 | 28 | 28 | 29 | 29 | 28 | 30 | 30 | 30 | 30 | 30 |
| Brain MRI/CT | | Normal | NA | NA | NA | Normal | Normal | Normal | NA | Normal | Normal | Normal | Normal | Normal |

TETRAS: The Tremor Research Group Essential Tremor Rating Assessment Scale; MMSE: Mini-Mental State Examination; MRI: magnetic resonance imaging; CT: Computed Tomography; WT=wild type; het=heterozygous; hom=homozygous.

Annotation: IV2 and IV14 carry heterozygous variants (c.842T>C/p.M281T; c.952G>A/p.G318S) in the *SLC38A6* gene but exhibit no clinical manifestations, suggesting the possibility of incomplete penetrance. IV12 does not carry the heterozygous variants in the *SLC38A6* gene and presents only with postural tremor in both upper limbs. The TETRAS I score is 0, and the TETRAS II score is 4, suggesting a possible phenocopy.

**Supplementary Table 2. Whole exome sequencing in family 1.**

|  | V:6 | III:13 | IV:17 |
| --- | --- | --- | --- |
| Number of raw sequenced base (Mb) | 10,842 | 10,153 | 10,741 |
| Number of raw reads | 10,8427,734 | 10,1537,026 | 10,7413,178 |
| Raw depth of target region | 114.29 | 109.61 | 116.32 |
| Coverage of target region (%) | 97.86 | 97.84 | 97.85 |
| Total variants | 112,274 | 109,512 | 110,123 |
| Variants with less than 5% frequency in gnomAD, 1000 Genomes and ExAC database | 7,282 | 6,930 | 7,253 |
| Variants causing amino acid changes or potential damaging | 451 | 438 | 432 |
| Heterozygous variants shared by three patients | *DPP6*:NM_130797:exon1:c.191G>A:p.G64D  *KMT5A*:NM_020382:exon4:c.364G>C:p.G122R  *KMT5A*:NM_020382:exon8:c.995T>C:p.L332P  *SLC38A6*:NM_001172702:exon12:c.842T>C:p.M281T  *SLC38A6*:NM_001172702:exon13: c.952G>A/p.G318S  *GGT1*:NM_001288833:exon7:c.365C>T:p.S122L | | |

**Supplementary Table 3. *SLC38A6* gene variants (NM_153811) identified in this study**

| Patients | Chromosome Position ^a^ | rs ID ^b^ | cDNA Alteration | Amino Acid Alteration | gnomAD_European ^c^ | gnomAD_East Asian ^d^ | Polyphen | SIFT | CADD |
| --- | --- | --- | --- | --- | --- | --- | --- | --- | --- |
| Family 02 | 14:61050538 | rs74825213 | c.952G>A | p.G318S | 0.0003 | 0.0278 | benign | tolerated | 20.5 |
| Family 03 | 14:60981293 | rs184560042 | c.16G>C | p.G6R | 0.00002 | 0.0098 | benign | deleterious | 15.1 |
| Family 04 | 14:61052415 | rs1447682241 | c.1357T>C | p.W453R | 0 | 0.0004 | probably_damaging | deleterious | 21.1 |
| Family 05 | 14:61043478 | rs145440362 | c.719C>A | p.P240Q | 0 | 0.0077 | probably_damaging | deleterious | 24.9 |
| Family 06 | 14:61050595 | rs187227863 | c.1009C>G | p.L337V | 0 | 0.0002 | probably_damaging | deleterious | 22.6 |
| Family 07 | 14:61046084 | rs117560154 | c.842T>C | p.M281T | 0.0003 | 0.0227 | probably_damaging | tolerated | 25.9 |
| Family 07 | 14:61050538 | rs74825213 | c.952G>A | p.G318S | 0.0003 | 0.0278 | benign | tolerated | 20.5 |
| Family 08 | 14:61046084 | rs117560154 | c.842T>C | p.M281T | 0.0003 | 0.0227 | probably_damaging | tolerated | 25.9 |
| Family 08 | 14:61050538-61050539 | - | c.952_953GG>AA | p.G318N | - | - | probably_damaging | tolerated | 17.22 |
| Family 09 | 14:61046084 | rs117560154 | c.842T>C | p.M281T | 0.0003 | 0.0227 | probably_damaging | tolerated | 25.9 |
| Family 09 | 14:61050538 | rs74825213 | c.952G>A | p.G318S | 0.0003 | 0.0278 | benign | tolerated | 20.5 |
| Family 10 | 14:61043478 | rs145440362 | c.719C>A | p.P240Q | 0 | 0.0077 | probably_damaging | deleterious | 24.9 |
| Family 10 | 14:61046084 | rs117560154 | c.842T>C | p.M281T | 0.0003 | 0.0227 | probably_damaging | tolerated | 25.9 |
| Family 10 | 14:61050538 | rs74825213 | c.952G>A | p.G318S | 0.0003 | 0.0278 | benign | tolerated | 20.5 |
| Family 11 | 14:61046084 | rs117560154 | c.842T>C | p.M281T | 0.0003 | 0.0227 | probably_damaging | tolerated | 25.9 |
| Family 11 | 14:61050538 | rs74825213 | c.952G>A | p.G318S | 0.0003 | 0.0278 | benign | tolerated | 20.5 |
| Family 12 | 14:61050538 | rs74825213 | c.952G>A | p.G318S | 0.0003 | 0.0278 | benign | tolerated | 20.5 |
| Family 13 | 14:61015916 | rs181093232 | c.323A>T | p.Y108F | 0.00002 | 0.0088 | probably_damaging | deleterious | 26.4 |
| Family 14 | 14:61046084 | rs117560154 | c.842T>C | p.M281T | 0.0003 | 0.0227 | probably_damaging | tolerated | 25.9 |
| Family 14 | 14:61050538 | rs74825213 | c.952G>A | p.G318S | 0.0003 | 0.0278 | benign | tolerated | 20.5 |
| Family 15 | 14:61050538 | rs74825213 | c.952G>A | p.G318S | 0.0003 | 0.0278 | benign | tolerated | 20.5 |
| Family 15 | 14:61051841 | rs201457210 | c.1105C>T | p.R369C | 0 | 0.0012 | benign | deleterious | 17.85 |
| Family 16 | 14:61046084 | rs117560154 | c.842T>C | p.M281T | 0.0003 | 0.0227 | probably_damaging | tolerated | 25.9 |
| Family 16 | 14:61050538 | rs74825213 | c.952G>A | p.G318S | 0.0003 | 0.0278 | benign | tolerated | 20.5 |
| Family 17 | 14:60981293 | rs184560042 | c.16G>C | p.G6R | 0.00002 | 0.0098 | benign | deleterious | 15.1 |
| Family 18 | 14:61043478 | rs145440362 | c.719C>A | p.P240Q | 0 | 0.0077 | probably_damaging | deleterious | 24.9 |
| Family 19 | 14:61043478 | rs145440362 | c.719C>A | p.P240Q | 0 | 0.0077 | probably_damaging | deleterious | 24.9 |
| Family 20 | 14:61052415 | rs1447682241 | c.1357T>C | p.W453R | 0 | 0.0004 | probably_damaging | deleterious | 21.1 |
| Family 21 | 14:61015916 | rs181093232 | c.323A>T | p.Y108F | 0.00002 | 0.0088 | probably_damaging | deleterious | 26.4 |
| Family 22 | 14:60981293 | rs184560042 | c.16G>C | p.G6R | 0.00002 | 0.0098 | benign | deleterious | 15.1 |
| Family 23 | 14:61043478 | rs145440362 | c.719C>A | p.P240Q | 0 | 0.0077 | probably_damaging | deleterious | 24.9 |
| Family 24 | 14:61015916 | rs181093232 | c.323A>T | p.Y108F | 0.00002 | 0.0088 | probably_damaging | deleterious | 26.4 |
| Family 25 | 14:61046084 | rs117560154 | c.842T>C | p.M281T | 0.0003 | 0.0227 | probably_damaging | tolerated | 25.9 |
| Family 25 | 14:61050538 | rs74825213 | c.952G>A | p.G318S | 0.0003 | 0.0278 | benign | tolerated | 20.5 |
| Family 26 | 14:60981293 | rs184560042 | c.16G>C | p.G6R | 0.00002 | 0.0098 | benign | deleterious | 15.1 |
| Family 27 | 14:61015916 | rs181093232 | c.323A>T | p.Y108F | 0.00002 | 0.0088 | probably_damaging | deleterious | 26.4 |
| Family 28 | 14:61046084 | rs117560154 | c.842T>C | p.M281T | 0.0003 | 0.0227 |  |  |  |
| Family 28 | 14:61050538 | rs74825213 | c.952G>A | p.G318S | 0.0003 | 0.0278 | benign | deleterious | 15.1 |
| Family 29 | 14:61043478 | rs145440362 | c.719C>A | p.P240Q | 0 | 0.0077 | probably_damaging | deleterious | 24.9 |
| Family 30 | 14:61043478 | rs145440362 | c.719C>A | p.P240Q | 0 | 0.0077 | probably_damaging | deleterious | 24.9 |
| Family 31 | 14:61046084 | rs117560154 | c.842T>C | p.M281T | 0.0003 | 0.0227 | probably_damaging | tolerated | 25.9 |
| Family 31 | 14:61050538 | rs74825213 | c.952G>A | p.G318S | 0.0003 | 0.0278 | benign | tolerated | 20.5 |
| Family 32 | 14:61015916 | rs181093232 | c.323A>T | p.Y108F | 0.00002 | 0.0088 | probably_damaging | deleterious | 26.4 |
| Family 33 | 14:61015916 | rs181093232 | c.323A>T | p.Y108F | 0.00002 | 0.0088 | probably_damaging | deleterious | 26.4 |
| Family 34 | 14:61046084 | rs117560154 | c.842T>C | p.M281T | 0.0003 | 0.0227 | probably_damaging | tolerated | 25.9 |
| Family 34 | 14:61050538 | rs74825213 | c.952G>A | p.G318S | 0.0003 | 0.0278 | benign | tolerated | 20.5 |
| Family 35 | 14:61046084 | rs117560154 | c.842T>C | p.M281T | 0.0003 | 0.0227 | probably_damaging | tolerated | 25.9 |
| Family 35 | 14:61050538 | rs74825213 | c.952G>A | p.G318S | 0.0003 | 0.0278 | benign | tolerated | 20.5 |
| Family 36 | 14:61037678 | - | c.619C>T | p.L207F | - | - | possibly_damaging | tolerated | 22.5 |
| Family 37 | 14:61015916 | rs181093232 | c.323A>T | p.Y108F | 0.00002 | 0.0088 | probably_damaging | deleterious | 26.4 |
| Family 38 | 14:61043478 | rs145440362 | c.719C>A | p.P240Q | 0 | 0.0077 | probably_damaging | deleterious | 24.9 |
| Family 39 | 14:61046084 | rs117560154 | c.842T>C | p.M281T | 0.0003 | 0.0227 | probably_damaging | tolerated | 25.9 |
| Family 39 | 14:61050538 | rs74825213 | c.952G>A | p.G318S | 0.0003 | 0.0278 | benign | tolerated | 20.5 |
| Family 40 | 14:61015916 | rs181093232 | c.323A>T | p.Y108F | 0.00002 | 0.0088 | probably_damaging | deleterious | 26.4 |
| Family 41 | 14:60981293 | rs184560042 | c.16G>C | p.G6R | 0.00002 | 0.0098 | benign | deleterious | 15.1 |
| Family 42 | 14:61050538 | rs74825213 | c.952G>A | p.G318S | 0.0003 | 0.0278 | benign | tolerated | 20.5 |
| Family 43 | 14:61043478 | rs145440362 | c.719C>A | p.P240Q | 0 | 0.0077 | probably_damaging | deleterious | 24.9 |
| Family 44 | 14:61050538 | rs74825213 | c.952G>A | p.G318S | 0.0003 | 0.0278 | benign | tolerated | 20.5 |
| Family 45 | 14:61015916 | rs181093232 | c.323A>T | p.Y108F | 0.00002 | 0.0088 | probably_damaging | deleterious | 26.4 |
| Family 46 | 14:61046084 | rs117560154 | c.842T>C | p.M281T | 0.0003 | 0.0227 | probably_damaging | tolerated | 25.9 |
| Family 46 | 14:61050538 | rs74825213 | c.952G>A | p.G318S | 0.0003 | 0.0278 | benign | tolerated | 20.5 |
| Family 47 | 14:61043478 | rs145440362 | c.719C>A | p.P240Q | 0 | 0.0077 | probably_damaging | deleterious | 24.9 |
| Family 48 | 14:61015916 | rs181093232 | c.323A>T | p.Y108F | 0.00002 | 0.0088 | probably_damaging | deleterious | 26.4 |
| Family 49 | 14:61046084 | rs117560154 | c.842T>C | p.M281T | 0.0003 | 0.0227 | probably_damaging | tolerated | 25.9 |
| Family 49 | 14:61050538 | rs74825213 | c.952G>A | p.G318S | 0.0003 | 0.0278 | benign | tolerated | 20.5 |
| Family 50 | 14:61046084 | rs117560154 | c.842T>C | p.M281T | 0.0003 | 0.0227 | probably_damaging | tolerated | 25.9 |
| Family 50 | 14:61050538 | rs74825213 | c.952G>A | p.G318S | 0.0003 | 0.0278 | benign | tolerated | 20.5 |
| Family 51 | 14:61046084 | rs117560154 | c.842T>C | p.M281T | 0.0003 | 0.0227 | probably_damaging | tolerated | 25.9 |
| Family 51 | 14:61050538 | rs74825213 | c.952G>A | p.G318S | 0.0003 | 0.0278 | benign | tolerated | 20.5 |
| Family 52 | 14:61046084 | rs117560154 | c.842T>C | p.M281T | 0.0003 | 0.0227 | probably_damaging | tolerated | 25.9 |
| Family 52 | 14:61050538 | rs74825213 | c.952G>A | p.G318S | 0.0003 | 0.0278 | benign | tolerated | 20.5 |
| Family 53 | 14:61015916 | rs181093232 | c.323A>T | p.Y108F | 0.00002 | 0.0088 | probably_damaging | deleterious | 26.4 |
| Family 54 | 14:61050538 | rs74825213 | c.952G>A | p.G318S | 0.0003 | 0.0278 | benign | tolerated | 20.5 |
| Family 54 | 14:61051841 | rs201457210 | c.1105C>T | p.R369C | 0 | 0.0012 | benign | deleterious | 17.85 |
| Family 55 | 14:61046084 | rs117560154 | c.842T>C | p.M281T | 0.0003 | 0.0227 | probably_damaging | tolerated | 25.9 |
| Family 55 | 14:61050538 | rs74825213 | c.952G>A | p.G318S | 0.0003 | 0.0278 | benign | tolerated | 20.5 |
| Family 56 | 14:61046084 | rs117560154 | c.842T>C | p.M281T | 0.0003 | 0.0227 | probably_damaging | tolerated | 25.9 |
| Family 56 | 14:61050538-61050539 | - | c.952_953GG>AA | p.G318N | - | - | probably_damaging | tolerated | 17.22 |
| Family 57 | 14:61015916 | rs181093232 | c.323A>T | p.Y108F | 0.00002 | 0.0088 | probably_damaging | deleterious | 26.4 |
| Family 58 | 14:61046084 | rs117560154 | c.842T>C | p.M281T | 0.0003 | 0.0227 | probably_damaging | tolerated | 25.9 |
| Family 58 | 14:61050538 | rs74825213 | c.952G>A | p.G318S | 0.0003 | 0.0278 | benign | tolerated | 20.5 |
| Family 59 | 14:61046084 | rs117560154 | c.842T>C | p.M281T | 0.0003 | 0.0227 | probably_damaging | tolerated | 25.9 |
| Family 59 | 14:61050538 | rs74825213 | c.952G>A | p.G318S | 0.0003 | 0.0278 | benign | tolerated | 20.5 |
| Family 60 | 14:61046084 | rs117560154 | c.842T>C | p.M281T | 0.0003 | 0.0227 | probably_damaging | tolerated | 25.9 |
| Family 60 | 14:61050538 | rs74825213 | c.952G>A | p.G318S | 0.0003 | 0.0278 | benign | tolerated | 20.5 |
| Family 61 | 14:61046084 | rs117560154 | c.842T>C | p.M281T | 0.0003 | 0.0227 | probably_damaging | tolerated | 25.9 |
| Family 61 | 14:61050538 | rs74825213 | c.952G>A | p.G318S | 0.0003 | 0.0278 | benign | tolerated | 20.5 |
| Family 62 | 14:61015916 | rs181093232 | c.323A>T | p.Y108F | 0.00002 | 0.0088 | probably_damaging | deleterious | 26.4 |
| Family 63 | 14:61046084 | rs117560154 | c.842T>C | p.M281T | 0.0003 | 0.0227 | probably_damaging | tolerated | 25.9 |
| Family 63 | 14:61050538 | rs74825213 | c.952G>A | p.G318S | 0.0003 | 0.0278 | benign | tolerated | 20.5 |
| Family 64 | 14:61046084 | rs117560154 | c.842T>C | p.M281T | 0.0003 | 0.0227 | probably_damaging | tolerated | 25.9 |
| Family 64 | 14:61050538 | rs74825213 | c.952G>A | p.G318S | 0.0003 | 0.0278 | benign | tolerated | 20.5 |
| Family 65 | 14:61046084 | rs117560154 | c.842T>C | p.M281T | 0.0003 | 0.0227 | probably_damaging | tolerated | 25.9 |
| Family 65 | 14:61050538 | rs74825213 | c.952G>A | p.G318S | 0.0003 | 0.0278 | benign | tolerated | 20.5 |
| Family 66 | 14:61046084 | rs117560154 | c.842T>C | p.M281T | 0.0003 | 0.0227 | probably_damaging | tolerated | 25.9 |
| Family 66 | 14:61050538 | rs74825213 | c.952G>A | p.G318S | 0.0003 | 0.0278 | benign | tolerated | 20.5 |
| Family 67 | 14:61046084 | rs117560154 | c.842T>C | p.M281T | 0.0003 | 0.0227 | probably_damaging | tolerated | 25.9 |
| Family 67 | 14:61050538 | rs74825213 | c.952G>A | p.G318S | 0.0003 | 0.0278 | benign | tolerated | 20.5 |
| Family 68 | 14:61046084 | rs117560154 | c.842T>C | p.M281T | 0.0003 | 0.0227 | probably_damaging | tolerated | 25.9 |
| Family 68 | 14:61050538 | rs74825213 | c.952G>A | p.G318S | 0.0003 | 0.0278 | benign | tolerated | 20.5 |
| Family 69 | 14:61046084 | rs117560154 | c.842T>C | p.M281T | 0.0003 | 0.0227 | probably_damaging | tolerated | 25.9 |
| Family 69 | 14:61050538 | rs74825213 | c.952G>A | p.G318S | 0.0003 | 0.0278 | benign | tolerated | 20.5 |
| Family 70 | 14:61046084 | rs117560154 | c.842T>C | p.M281T | 0.0003 | 0.0227 | probably_damaging | tolerated | 25.9 |
| Family 70 | 14:61050538 | rs74825213 | c.952G>A | p.G318S | 0.0003 | 0.0278 | benign | tolerated | 20.5 |
| Family 71 | 14:61046084 | rs117560154 | c.842T>C | p.M281T | 0.0003 | 0.0227 | probably_damaging | tolerated | 25.9 |
| Family 71 | 14:61050538-61050539 | - | c.952_953GG>AA | p.G318N | - | - | probably_damaging | tolerated | 17.22 |
| Sporadic patient 01 | 14:60981293 | rs184560042 | c.16G>C | p.G6R | 0.00002 | 0.0098 | benign | deleterious | 15.1 |
| Sporadic patient 02 | 14:61046084 | rs117560154 | c.842T>C | p.M281T | 0.0003 | 0.0227 | probably_damaging | tolerated | 25.9 |
| Sporadic patient 02 | 14:61050538 | rs74825213 | c.952G>A | p.G318S | 0.0003 | 0.0278 | benign | tolerated | 20.5 |
| Sporadic patient 03 | 14:61015943 | rs749369988 | c.350G>A | p.G117E | 0 | 0 | probably_damaging | deleterious | 29.1 |
| Sporadic patient 04 | 14:61046084 | rs117560154 | c.842T>C | p.M281T | 0.0003 | 0.0227 | probably_damaging | tolerated | 25.9 |
| Sporadic patient 04 | 14:61050538 | rs74825213 | c.952G>A | p.G318S | 0.0003 | 0.0278 | benign | tolerated | 20.5 |
| Sporadic patient 05 | 14:60981293 | rs184560042 | c.16G>C | p.G6R | 0.00002 | 0.0098 | benign | deleterious | 15.1 |
| Sporadic patient 06 | 14:61046084 | rs117560154 | c.842T>C | p.M281T | 0.0003 | 0.0227 | probably_damaging | tolerated | 25.9 |
| Sporadic patient 06 | 14:61050538 | rs74825213 | c.952G>A | p.G318S | 0.0003 | 0.0278 | benign | tolerated | 20.5 |
| Sporadic patient 07 | 14:60981293 | rs184560042 | c.16G>C | p.G6R | 0.00002 | 0.0098 | benign | deleterious | 15.1 |
| Sporadic patient 08 | 14:61046084 | rs117560154 | c.842T>C | p.M281T | 0.0003 | 0.0227 | probably_damaging | tolerated | 25.9 |
| Sporadic patient 08 | 14:61050538 | rs74825213 | c.952G>A | p.G318S | 0.0003 | 0.0278 | benign | tolerated | 20.5 |
| Sporadic patient 09 | 14:61043478 | rs145440362 | c.719C>A | p.P240Q | 0 | 0.0077 | probably_damaging | deleterious | 24.9 |
| Sporadic patient 10 | 14:61046084 | rs117560154 | c.842T>C | p.M281T | 0.0003 | 0.0227 | probably_damaging | tolerated | 25.9 |
| Sporadic patient 10 | 14:61050538 | rs74825213 | c.952G>A | p.G318S | 0.0003 | 0.0278 | benign | tolerated | 20.5 |
| Sporadic patient 11 | 14:61015916 | rs181093232 | c.323A>T | p.Y108F | 0.00002 | 0.0088 | probably_damaging | deleterious | 26.4 |
| Sporadic patient 12 | 14:61043478 | rs145440362 | c.719C>A | p.P240Q | 0 | 0.0077 | probably_damaging | deleterious | 24.9 |
| Sporadic patient 13 | 14:61015916 | rs181093232 | c.323A>T | p.Y108F | 0.00002 | 0.0088 | probably_damaging | deleterious | 26.4 |
| Sporadic patient 13 | 14:61046084 | rs117560154 | c.842T>C | p.M281T | 0.0003 | 0.0227 | probably_damaging | tolerated | 25.9 |
| Sporadic patient 13 | 14:61050538 | rs74825213 | c.952G>A | p.G318S | 0.0003 | 0.0278 | benign | tolerated | 20.5 |
| Sporadic patient 14 | 14:61050595 | rs187227863 | c.1009C>G | p.L337V | 0 | 0.0002 | probably_damaging | deleterious | 22.6 |
| Sporadic patient 15 | 14:61015916 | rs181093232 | c.323A>T | p.Y108F | 0.00002 | 0.0088 | probably_damaging | deleterious | 26.4 |
| Sporadic patient 16 | 14:61030469 | rs781249288 | c.428T>C | p.I143T | - | - | probably_damaging | deleterious | 25.4 |
| Sporadic patient 17 | 14:61046084 | rs117560154 | c.842T>C | p.M281T | 0.0003 | 0.0227 | probably_damaging | tolerated | 25.9 |
| Sporadic patient 17 | 14:61050538 | rs74825213 | c.952G>A | p.G318S | 0.0003 | 0.0278 | benign | tolerated | 20.5 |
| Sporadic patient 18 | 14:61015916 | rs181093232 | c.323A>T | p.Y108F | 0.00002 | 0.0088 | probably_damaging | deleterious | 26.4 |
| Sporadic patient 19 | 14:61015916 | rs181093232 | c.323A>T | p.Y108F | 0.00002 | 0.0088 | probably_damaging | deleterious | 26.4 |
| Sporadic patient 20 | 14:61015916 | rs181093232 | c.323A>T | p.Y108F | 0.00002 | 0.0088 | probably_damaging | deleterious | 26.4 |
| Sporadic patient 21 | 14:60981342 | rs1305067041 | c.65A>G | p.E22G | 0.00003 | 0 | benign | deleterious | 23 |
| Sporadic patient 22 | 14:61046084 | rs117560154 | c.842T>C | p.M281T | 0.0003 | 0.0227 | probably_damaging | tolerated | 25.9 |
| Sporadic patient 22 | 14:61050538 | rs74825213 | c.952G>A | p.G318S | 0.0003 | 0.0278 | benign | tolerated | 20.5 |
| Sporadic patient 23 | 14:61046084 | rs117560154 | c.842T>C | p.M281T | 0.0003 | 0.0227 | probably_damaging | tolerated | 25.9 |
| Sporadic patient 23 | 14:61050538 | rs74825213 | c.952G>A | p.G318S | 0.0003 | 0.0278 | benign | tolerated | 20.5 |
| Sporadic patient 24 | 14:61043478 | rs145440362 | c.719C>A | p.P240Q | 0 | 0.0077 | probably_damaging | deleterious | 24.9 |
| Sporadic patient 25 | 14:61046084 | rs117560154 | c.842T>C | p.M281T | 0.0003 | 0.0227 | probably_damaging | tolerated | 25.9 |
| Sporadic patient 25 | 14:61050538 | rs74825213 | c.952G>A | p.G318S | 0.0003 | 0.0278 | benign | tolerated | 20.5 |
| Sporadic patient 26 | 14:61046084 | rs117560154 | c.842T>C | p.M281T | 0.0003 | 0.0227 | probably_damaging | tolerated | 25.9 |
| Sporadic patient 26 | 14:61050538 | rs74825213 | c.952G>A | p.G318S | 0.0003 | 0.0278 | benign | tolerated | 20.5 |
| Sporadic patient 27 | 14:61015916 | rs181093232 | c.323A>T | p.Y108F | 0.00002 | 0.0088 | probably_damaging | deleterious | 26.4 |
| Sporadic patient 28 | 14:61050538 | rs74825213 | c.952G>A | p.G318S | 0.0003 | 0.0278 | benign | tolerated | 20.5 |
| Sporadic patient 28 | 14:61051841 | rs201457210 | c.1105C>T | p.R369C | 0 | 0.0012 | benign | deleterious | 17.85 |
| Sporadic patient 29 | 14:61015916 | rs181093232 | c.323A>T | p.Y108F | 0.00002 | 0.0088 | probably_damaging | deleterious | 26.4 |
| Sporadic patient 30 | 14:61050538 | rs74825213 | c.952G>A | p.G318S | 0.0003 | 0.0278 | benign | tolerated | 20.5 |
| Sporadic patient 30 | 14:61051841 | rs201457210 | c.1105C>T | p.R369C | 0 | 0.0012 | benign | deleterious | 17.85 |
| Sporadic patient 31 | 14:61015916 | rs181093232 | c.323A>T | p.Y108F | 0.00002 | 0.0088 | probably_damaging | deleterious | 26.4 |
| Sporadic patient 32 | 14:61015916 | rs181093232 | c.323A>T | p.Y108F | 0.00002 | 0.0088 | probably_damaging | deleterious | 26.4 |
| Sporadic patient 32 | 14:61046084 | rs117560154 | c.842T>C | p.M281T | 0.0003 | 0.0227 | probably_damaging | tolerated | 25.9 |
| Sporadic patient 32 | 14:61050538 | rs74825213 | c.952G>A | p.G318S | 0.0003 | 0.0278 | benign | tolerated | 20.5 |
| Sporadic patient 33 | 14:61046084 | rs117560154 | c.842T>C | p.M281T | 0.0003 | 0.0227 | probably_damaging | tolerated | 25.9 |
| Sporadic patient 33 | 14:61050538 | rs74825213 | c.952G>A | p.G318S | 0.0003 | 0.0278 | benign | tolerated | 20.5 |
| Sporadic patient 34 | 14:61015916 | rs181093232 | c.323A>T | p.Y108F | 0.00002 | 0.0088 | probably_damaging | deleterious | 26.4 |
| Sporadic patient 35 | 14:61046084 | rs117560154 | c.842T>C | p.M281T | 0.0003 | 0.0227 | probably_damaging | tolerated | 25.9 |
| Sporadic patient 35 | 14:61050538 | rs74825213 | c.952G>A | p.G318S | 0.0003 | 0.0278 | benign | tolerated | 20.5 |
| Sporadic patient 36 | 14:61046084 | rs117560154 | c.842T>C | p.M281T | 0.0003 | 0.0227 | probably_damaging | tolerated | 25.9 |
| Sporadic patient 36 | 14:61050538 | rs74825213 | c.952G>A | p.G318S | 0.0003 | 0.0278 | benign | tolerated | 20.5 |
| Sporadic patient 37 | 14:60981293 | rs184560042 | c.16G>C | p.G6R | 0.00002 | 0.0098 | benign | deleterious | 15.1 |
| Sporadic patient 38 | 14:61046084 | rs117560154 | c.842T>C | p.M281T | 0.0003 | 0.0227 | probably_damaging | tolerated | 25.9 |
| Sporadic patient 38 | 14:61050538 | rs74825213 | c.952G>A | p.G318S | 0.0003 | 0.0278 | benign | tolerated | 20.5 |
| Sporadic patient 39 | 14:61046084 | rs117560154 | c.842T>C | p.M281T | 0.0003 | 0.0227 | probably_damaging | tolerated | 25.9 |
| Sporadic patient 39 | 14:61050538 | rs74825213 | c.952G>A | p.G318S | 0.0003 | 0.0278 | benign | tolerated | 20.5 |
| Sporadic patient 40 | 14:61046084 | rs117560154 | c.842T>C | p.M281T | 0.0003 | 0.0227 | probably_damaging | tolerated | 25.9 |
| Sporadic patient 40 | 14:61050538 | rs74825213 | c.952G>A | p.G318S | 0.0003 | 0.0278 | benign | tolerated | 20.5 |
| Sporadic patient 41 | 14:61015916 | rs181093232 | c.323A>T | p.Y108F | 0.00002 | 0.0088 | probably_damaging | deleterious | 26.4 |
| Sporadic patient 42 | 14:61015916 | rs181093232 | c.323A>T | p.Y108F | 0.00002 | 0.0088 | probably_damaging | deleterious | 26.4 |
| Sporadic patient 43 | 14:61046084 | rs117560154 | c.842T>C | p.M281T | 0.0003 | 0.0227 | probably_damaging | tolerated | 25.9 |
| Sporadic patient 43 | 14:61050538 | rs74825213 | c.952G>A | p.G318S | 0.0003 | 0.0278 | benign | tolerated | 20.5 |
| Sporadic patient 44 | 14:61046084 | rs117560154 | c.842T>C | p.M281T | 0.0003 | 0.0227 | probably_damaging | tolerated | 25.9 |
| Sporadic patient 44 | 14:61050538 | rs74825213 | c.952G>A | p.G318S | 0.0003 | 0.0278 | benign | tolerated | 20.5 |
| Sporadic patient 45 | 14:61046084 | rs117560154 | c.842T>C | p.M281T | 0.0003 | 0.0227 | probably_damaging | tolerated | 25.9 |
| Sporadic patient 45 | 14:61050538 | rs74825213 | c.952G>A | p.G318S | 0.0003 | 0.0278 | benign | tolerated | 20.5 |
| Sporadic patient 46 | 14:61037142 | rs759968619 | c.565+1G>A |  | 0 | 0.0002 | NA | NA | 33 |
| Sporadic patient 47 | 14:61043504 | rs200309894 | c.744+1G>A |  | 0 | 0.0004 | NA | NA | 34 |

^a^ Position on Genome Reference Consortium human genome build 38 (GRCh38).

^b^ ID in dbSNP.

^c^ The frequency of the variant in total population of the Genome Aggregation Database.

^d^ The frequency of the variant in East Asian population of the Genome Aggregation Database.

Polyphen 2 (version 2.2.3r408): Polymorphism Phenotyping 2; SIFT (SIFT 4G): Sorting Intolerant From Tolerant; CADD (GRCh38-v1.6): combined annotation-dependent depletion score. NA: not available.

**Supplementary Table 4. Distribution and association analysis of *SLC38A6* variants in case-control samples**

| **rsID** | **VarID (hg38)** | **variant** | **Case_geno** | **Control_geno** | **Case_MAF** | **Control_MAF** | **gnomAD_EAS_v3.1** | **gnomAD_EUR_v3.1** |
| --- | --- | --- | --- | --- | --- | --- | --- | --- |
| rs184560042 | 14:60981293 | exon1:c.16G>C:p.G6R | 0/9/1404 | 0/10/1064 | 0.0032 | 0.0047 | 0.0098 | 0 |
| rs1305067041 | 14:60981342 | exon1:c.65A>G:p.E22G | 0/1/1412 | 0/0/1073 | 0.0004 | 0 | 0 | 0.00003 |
| rs749369988 | 14:61015943 | exon4:c.350G>A:p.G117E | 0/1/1412 | 0/0/1073 | 0.0004 | 0 | 0 | 0.00003 |
| rs181093232 | 14:61015916 | exon4:c.323A>T:p.Y108F | 0/26/1387 | 0/22/1052 | 0.0092 | 0.0102 | 0.0088 | 0.00003 |
| rs781249288 | 14:61030469 | exon6:c.428T>C:p.I143T | 0/1/1412 | 0/1/1073 | 0.0004 | 0.0005 | - | - |
| rs759968619 | 14:61037142 | exon7:c.565+1G>A | 0/1/1412 | 0/0/1074 | 0.0004 | 0 | 0.0002 | 0 |
| - | 14:61037678 | exon8:c.619C>T:p.L207F | 0/1/1412 | 0/0/1073 | 0.0004 | 0 | - | - |
| rs145440362 | 14:61043478 | exon10:c.719C>A:p.P240Q | 0/13/1400 | 0/12/1060 | 0.0046 | 0.0056 | 0.0077 | 0 |
| rs200309894 | 14:61043504 | exon10:c.744+1G>A | 0/1/1412 | 0/0/1070 | 0.0004 | 0 | 0.0004 | 0 |
| rs117560154 | 14:61046084 | exon12:c.842T>C:p.M281T | 0/55/1359 | 0/29/1045 | 0.0195 | 0.0135 | 0.0227 | 0.0003 |
| rs74825213 | 14:61050538 | exon13:c.952G>A:p.G318S | 0/63/1351 | 0/36/1038 | 0.0223 | 0.0168 | 0.0278 | 0.0003 |
| - | 14:61050538-61050539 | exon13:c.952-953GG>AA:p.G318N | 0/3/1410 | 0/0/1074 | 0.0011 | 0 | - | - |
| rs187227863 | 14:61050595 | exon13:c.1009C>G:p.L337V | 0/2/1411 | 0/1/1073 | 0.0007 | 0.0005 | 0.0002 | 0 |
| rs201457210 | 14:61051841 | exon14:c.1105C>T:p.R369C | 0/4/1409 | 0/4/1070 | 0.0014 | 0.0019 | 0.0012 | 0 |
| rs1447682241 | 14:61052415 | exon16:c.1357T>C:p.W453R | 0/2/1411 | 0/1/1073 | 0.0007 | 0.0005 | 0.0004 | 0 |

**Supplementary Table 5. Clinical characteristics of patients with/without variation of *SLC38A6***

|  | All patients  (n=1413) | Carrier  (n=118) | Non-carrier  (n=1295) | P value |
| --- | --- | --- | --- | --- |
| Age (years) | 53.69±16.49 | 54.99±15.63 | 53.53±16.59 | 0.502 |
| Sex-female (%) | 53.27 | 58.96 | 52.58 | 0.163 |
| Age at onset (years) | 42.75±17.32 | 44.55±16.28 | 42.53±17.44 | 0.316 |
| Duration (years) | 10.93±9.19 | 10.44±8.44 | 10.99±9.28 | 0.866 |
| Tremor distribution |  |  |  |  |
| Head (%) | 29.79 | 29.58 | 29.82 | 0.967 |
| Face (%) | 24.49 | 22.54 | 24.75 | 0.686 |
| Voice (%) | 23.94 | 19.72 | 24.54 | 0.373 |
| Upper limbs (%) | 100 | 100 | 100 | - |
| Lower limbs (%) | 14.31 | 13.64 | 14.40 | 0.635 |
| Tremor types |  |  |  |  |
| Posture tremor (%) | 100 | 100 | 100 | - |
| Kinetic tremor (%) | 98.58 | 97.52 | 98.76 | 0.863 |
| Intention tremor (%) | 38.28 | 45.10 | 37.44 | 0.289 |
| Tremor severity |  |  |  |  |
| TETRAS-I | 13.75±9.67 | 13.89±9.21 | 13.74±9.74 | 0.730 |
| TETRAS-II | 18.77±7.95 | 19.11±7.22 | 18.72±8.05 | 0.569 |

TETRAS: The Tremor Research Group Essential Tremor Rating Assessment Scale

**Supplementary Table 6. Primer sequences for mutation analysis of *SLC38A6***

| Mutation | Forward primer sequence（5´-3´） | Reverse primer sequence（5´-3´） | Amplicon Size (bp) |
| --- | --- | --- | --- |
| exon1:c.16G>C:p.G6R | GCGGGTCTTCTATGACATCATCACT | AGCTTCCACTCAACGCTGCAT | 575 |
| exon1:c.65A>G:p.E22G | GCGGGTCTTCTATGACATCATCACT | AGCTTCCACTCAACGCTGCAT | 575 |
| exon4:c.350G>A:p.G117E | AACAGCTGATTAAAGCCAACCA | GGTACTGGCTTTATATAATAGTTGACA | 796 |
| exon4:c.323A>T:p.Y108F | AACAGCTGATTAAAGCCAACCA | GGTACTGGCTTTATATAATAGTTGACA | 796 |
| exon6:c.428T>C:p.I143T | ACAGATAATCCAGTTACTAGCCAT | CATATCCCCAGAAATTTACCCTT | 839 |
| exon7:c.565+1G>A | TCCTAATAGCTGGTTGTAATGGTT | AAATGCTGTTCTAGCTAAGCCTC | 951 |
| exon8:c.619C>T:p.L207F | TCCTAATAGCTGGTTGTAATGGTT | AAATGCTGTTCTAGCTAAGCCTC | 951 |
| exon10:c.719C>A:p.P240Q | TAAATGTTGACCCCATTCTAACCG | TCCATTCAAAGGCATACCAG | 747 |
| exon10:c.744+1G>A | TAAATGTTGACCCCATTCTAACCG | TCCATTCAAAGGCATACCAG | 747 |
| exon12:c.842T>C:p.M281T | AATGAGGAATTCTCTCGACCA | AAGAATCATGTCCCTAAGCCT | 460 |
| exon13:c.952_953GG>AA:p.G318N | AGCACAAATGTTCAAACCC | AGCACAAATTAGCTTAGTGG | 628 |
| exon13:c.952G>A:p.G318S | AGCACAAATGTTCAAACCC | AGCACAAATTAGCTTAGTGG | 628 |
| exon13:c.1009C>G:p.L337V | AGCACAAATGTTCAAACCC | AGCACAAATTAGCTTAGTGG | 628 |
| exon14:c.1105C>T:p.R369C | TATGAAGCTTACACAGAAAGGGTT | AAGATGAGCAAAACGAATGCCT | 781 |
| exon16:c.1357T>C:p.W453R | TTCCACAGGCATTCGTTTTG | TTCATCCAGGGTGTCTTCCG | 171 |

**Supplementary Table 7. Clinical characterization of *SLC38A6* co-segregated in ET families**

| Patient ID | Sex | Age | AAO | TETRAS Ⅰ | TETRAS Ⅱ |
| --- | --- | --- | --- | --- | --- |
| Family 02-II3 | M | 58 | 12 | 9 | 18 |
| Family 02-II4 | M | 54 | 44 | 8 | 16 |
| Family 07-II3 | F | 68 | 48 | 32 | 37 |
| Family 07-II4 | F | 61 | 51 | 6 | 17 |
| Family 26-II2 | F | 80 | 60 | 6 | 12 |
| Family 26-II5 | F | 70 | 62 | 20 | 20 |
| Family 27-I2 | F | 60 | 45 | 1 | 10 |
| Family 27-II1 | M | 38 | 12 | 10 | 20 |
| Family 27-II3 | M | 36 | 36 | 2 | 10 |
| Family 27-III1 | M | 16 | 13 | 8 | 16 |
| Family 27-III3 | M | 11 | 10 | 7 | 18 |
| Family 33-I1 | M | 57 | 52 | 15 | 20 |
| Family 33-II1 | F | 35 | 35 | 2 | 5 |
| Family 33-II2 | F | 33 | 31 | 3 | 8 |
| Family 33-II3 | M | 26 | 26 | 2 | 8 |
| Family 36-I2 | F | 73 | 68 | 8 | 16 |
| Family 36-II1 | F | 47 | 45 | 10 | 18 |
| Family 36-II2 | F | 45 | 40 | 6 | 12 |
| Family 38-I2 | F | 50 | 40 | 9 | 14 |
| Family 38-II1 | F | 35 | 25 | 12 | 16 |
| Family 41-II2 | M | 70 | 50 | 28 | 36 |
| Family 41-II3 | M | 66 | 61 | 18 | 24 |
| Family 42-I2 | F | 63 | 30 | 25 | 36 |
| Family 42-II2 | F | 37 | 36 | 4 | 11 |
| Family 47-I2 | F | 46 | 39 | 3 | 10 |
| Family 47-II1 | M | 20 | 16 | 3 | 6 |

**Supplementary Table 8. The semiquantitative scores of the basket cell plexus morphologies in *Slc38a6*^+/+^ and *Slc38a6*^-/-^ mice.**

|  | *Slc38a6*^+/+^ | | | | *Slc38a6*^-/-^ | | | |  |
| --- | --- | --- | --- | --- | --- | --- | --- | --- | --- |
| Age | 1(low) | 2(intermediate) | 3(high) | Average | 1(low) | 2(intermediate) | 3(high) | Average | P-value^*^ |
| 2M | 6 | 0 | 0 | 1.00 ± 0.00 | 5 | 1 | 0 | 1.17 ± 0.17 | 0.9851 |
| 4M | 6 | 0 | 0 | 1.00 ± 0.00 | 4 | 2 | 0 | 1.33 ± 0.21 | 0.7733 |
| 6M | 5 | 1 | 0 | 1.17 ± 0.17 | 4 | 2 | 0 | 1.33 ± 0.21 | 0.9851 |
| 9M | 5 | 1 | 0 | 1.17 ± 0.17 | 3 | 2 | 1 | 1.67 ± 0.33 | 0.3811 |
| 12.5M | 4 | 2 | 0 | 1.33 ± 0.21 | 1 | 3 | 2 | 2.17 ± 0.31 | 0.0298 |

* Compared to *Slc38a6*^+/+^, significant difference was determined by two-way ANOVA followed by Sidak's multiple comparisons test.

**Supplementary Table 9. The semiquantitative scores of the basket cell plexus morphologies in control and *Slc38a6*^PC^*^-/-^* mice.**

|  | Control | | | | *Slc38a6*^PC^*^-/-^* | | | |  |
| --- | --- | --- | --- | --- | --- | --- | --- | --- | --- |
| Age | 1(low) | 2(intermediate) | 3(high) | Average | 1(low) | 2(intermediate) | 3(high) | Average | P-value^*^ |
| 2M | 4 | 0 | 0 | 1.00 ± 0.00 | 4 | 0 | 0 | 1.00 ± 0.00 | >0.9999 |
| 4M | 4 | 0 | 0 | 1.00 ± 0.00 | 2 | 2 | 0 | 1.50 ± 0.29 | 0.388 |
| 6M | 3 | 1 | 0 | 1.25 ± 0.25 | 1 | 3 | 0 | 1.75 ± 0.25 | 0.388 |
| 9M | 2 | 2 | 0 | 1.50 ± 0.29 | 0 | 4 | 0 | 1.00 ± 0.00 | 0.388 |
| 12.5M | 1 | 3 | 0 | 1.75 ± 0.25 | 0 | 1 | 3 | 2.75 ± 0.25 | 0.0081 |

* Compared to control, significant difference was determined by two-way ANOVA followed by Sidak's multiple comparisons test.

**Supplementary Table 10.** **128 DEPs in the cerebellum of 2 month-old *Slc38a6*^+/+^ and *Slc38a6*^-/-^ mice.**

| Protein Name | Protein level in *Slc38a6*^-/-^ mice | | | Protein level in *Slc38a6*^+/+^ mice | | | log_2_  FoldChange | log_10_Padj |
| --- | --- | --- | --- | --- | --- | --- | --- | --- |
|  | Sample1 | Sample2 | Sample3 | Sample1 | Sample2 | Sample3 |  |  |
| TONSL | 27523.8 | 20573.2 | 27715.5 | 76729.3 | 55128.7 | 99674.8 | -1.61 | -9.96 |
| ANAPC11 | 6455.9 | 6395.8 | 6066.5 | 12442.8 | 17305.9 | 16329.3 | -1.28 | -5.14 |
| ADI1 | 217382.6 | 248924.7 | 220321.1 | 547374.9 | 527744.4 | 512132.1 | -1.21 | -64.44 |
| ACTR5 | 5980.8 | 4421.5 | 5688.7 | 11881.4 | 8424.7 | 16155.7 | -1.18 | -1.66 |
| NADK | 7655.4 | 14317.8 | 7084.7 | 17394.9 | 18941.5 | 28002.9 | -1.15 | -1.43 |
| NNT | 71831.1 | 71584.3 | 90126.6 | 188430.3 | 144648.3 | 183717.9 | -1.15 | -13.88 |
| MPST | 309870.6 | 307442.4 | 307564.0 | 614881.2 | 698443.7 | 728453.5 | -1.14 | -57.31 |
| SLC45A1 | 6358.3 | 6756.8 | 7257.0 | 13477.1 | 17312.8 | 12609.3 | -1.09 | -3.14 |
| TMTC4 | 31664.5 | 17237.8 | 15162.6 | 55873.6 | 35519.8 | 43362.2 | -1.07 | -1.77 |
| MRPL48 | 10157.0 | 8255.4 | 10521.4 | 17778.4 | 17161.9 | 23251.6 | -1.01 | -2.86 |
| ACY1 | 13397.7 | 16169.5 | 9272.4 | 26666.1 | 17770.2 | 33652.6 | -1.01 | -1.36 |
| GANC | 19617.1 | 21548.5 | 21098.1 | 40525.6 | 35794.8 | 43732.4 | -0.95 | -7.94 |
| SNX1 | 19778.7 | 19322.9 | 13840.1 | 34533.3 | 32300.7 | 30711.3 | -0.88 | -3.11 |
| DDX3Y | 24473.3 | 25454.6 | 30888.2 | 51765.0 | 45607.7 | 47038.6 | -0.84 | -5.53 |
| NFYA | 12441.7 | 15592.8 | 16218.2 | 24373.4 | 27276.5 | 24214.3 | -0.78 | -2.59 |
| SERPINA1E | 40713.6 | 33783.2 | 41489.2 | 76375.3 | 56516.9 | 59980.6 | -0.73 | -3.11 |
| TRAF7 | 11632.5 | 10580.9 | 11343.2 | 17332.0 | 17932.9 | 18545.9 | -0.68 | -2.24 |
| HMGCS2 | 17275.2 | 14877.7 | 20164.4 | 27217.6 | 30199.7 | 23915.9 | -0.64 | -1.31 |
| PANX2 | 26801.4 | 26095.6 | 24261.8 | 38250.6 | 39695.1 | 41679.0 | -0.63 | -4.31 |
| MANBAL | 34468.6 | 29628.8 | 30259.7 | 47605.3 | 55614.8 | 39875.1 | -0.60 | -1.74 |
| ADAL | 45606.3 | 47259.3 | 42864.0 | 57623.6 | 74155.0 | 70323.4 | -0.57 | -2.73 |
| SNX18 | 40703.7 | 43469.9 | 39305.5 | 61593.6 | 59750.7 | 58702.8 | -0.54 | -4.84 |
| PPP1R37 | 71035.6 | 52224.8 | 68559.1 | 79793.8 | 90887.1 | 107727.2 | -0.54 | -1.39 |
| GNPAT | 17068.2 | 16616.6 | 17823.7 | 24751.9 | 25474.2 | 24355.7 | -0.53 | -1.95 |
| COL12A1 | 25513.1 | 30403.3 | 29083.3 | 40880.5 | 42337.4 | 35537.5 | -0.48 | -1.32 |
| ACADL | 1198935.4 | 1259287.7 | 1191796.5 | 1674694.7 | 1693889.9 | 1728386.0 | -0.48 | -29.50 |
| FTL1 | 139742.2 | 169566.8 | 208245.6 | 259283.3 | 234973.4 | 220248.3 | -0.47 | -1.46 |
| HPX | 180467.7 | 181171.4 | 189062.2 | 259263.6 | 246695.0 | 241912.3 | -0.44 | -10.02 |
| D6WSU163E | 61366.6 | 52274.2 | 65660.3 | 74647.0 | 85602.5 | 82494.0 | -0.44 | -1.58 |
| VDAC3 | 477808.9 | 380008.9 | 462342.6 | 535426.7 | 606509.6 | 637120.2 | -0.43 | -2.85 |
| PPWD1 | 32605.8 | 35627.9 | 32406.1 | 43398.4 | 48028.1 | 42478.5 | -0.41 | -1.41 |
| ACADS | 56025.4 | 56823.3 | 51576.4 | 74987.1 | 68483.0 | 74722.3 | -0.41 | -2.37 |
| FRRS1L | 330878.4 | 340423.4 | 312049.1 | 430884.0 | 426522.9 | 433155.4 | -0.39 | -10.02 |
| NBDY | 77166.3 | 72516.0 | 73721.0 | 92321.1 | 99653.9 | 100865.2 | -0.39 | -3.14 |
| NOS1 | 119212.5 | 112315.2 | 117845.1 | 152688.7 | 149431.0 | 153998.4 | -0.38 | -5.98 |
| ITIH3 | 86465.8 | 93260.3 | 98804.7 | 125322.6 | 119584.2 | 115756.7 | -0.37 | -2.60 |
| UBE2A | 44994.2 | 47230.6 | 48066.3 | 56028.0 | 64642.8 | 59899.5 | -0.36 | -1.40 |
| RIDA | 972348.9 | 952150.9 | 979349.8 | 1181957.6 | 1186131.1 | 1301407.3 | -0.34 | -7.83 |
| ASNS | 114913.1 | 113550.6 | 108108.3 | 138255.0 | 147134.8 | 132077.7 | -0.31 | -2.19 |
| GLOD4 | 280360.7 | 271510.8 | 276570.0 | 347638.3 | 333305.1 | 341040.2 | -0.30 | -6.88 |
| ARSA | 124751.7 | 124234.4 | 125111.1 | 159217.9 | 145241.8 | 155994.6 | -0.30 | -2.68 |
| CWF19L1 | 65780.2 | 68639.6 | 69121.1 | 84844.2 | 80144.5 | 84806.2 | -0.30 | -1.63 |
| ITPKA | 416019.5 | 416636.9 | 414508.0 | 496429.8 | 510974.9 | 509005.8 | -0.28 | -8.79 |
| GUK1 | 214019.6 | 216315.7 | 206008.6 | 255253.3 | 238521.7 | 279822.4 | -0.28 | -1.77 |
| PPIH | 86235.1 | 93743.0 | 86640.6 | 106236.7 | 106610.5 | 110741.2 | -0.28 | -1.58 |
| CDIP1 | 198081.9 | 207113.7 | 221641.6 | 262258.3 | 238361.0 | 259392.5 | -0.28 | -1.90 |
| SERPINA1D | 452551.0 | 446163.8 | 464189.9 | 560645.5 | 508937.0 | 581725.6 | -0.28 | -2.83 |
| AQR | 72951.7 | 70172.8 | 74945.8 | 87341.4 | 87431.4 | 88778.9 | -0.27 | -1.52 |
| KCNIP3 | 153871.3 | 168816.0 | 163506.1 | 194971.2 | 204603.1 | 186226.6 | -0.27 | -1.77 |
| DNPEP | 284566.4 | 294542.0 | 307552.9 | 364284.1 | 353237.5 | 344557.4 | -0.26 | -3.13 |
| FABP7 | 679741.7 | 731179.4 | 741880.9 | 808387.6 | 805673.4 | 956501.6 | -0.26 | -1.36 |
| ENPP6 | 339213.9 | 367572.4 | 358149.3 | 446106.1 | 425238.3 | 396810.0 | -0.25 | -2.02 |
| APEX1 | 173039.9 | 179840.0 | 169285.5 | 199925.3 | 198750.7 | 220570.6 | -0.25 | -1.45 |
| KCND2 | 486271.5 | 488635.6 | 496060.1 | 559485.4 | 549138.9 | 608273.0 | -0.22 | -2.24 |
| RBM3 | 554334.5 | 573187.5 | 620724.1 | 680907.9 | 650373.2 | 708875.2 | -0.22 | -1.69 |
| DPP6 | 612423.8 | 562863.8 | 544084.1 | 645735.5 | 658202.4 | 700661.7 | -0.22 | -1.58 |
| TUBA8 | 420684.5 | 434723.4 | 398449.0 | 479143.1 | 474127.1 | 507326.7 | -0.22 | -1.98 |
| CPNE9 | 264424.0 | 254403.6 | 269534.6 | 290250.7 | 315727.2 | 303378.6 | -0.21 | -1.47 |
| TNC | 522095.4 | 490892.8 | 532052.9 | 610389.2 | 576160.0 | 584433.0 | -0.20 | -1.77 |
| SLC1A6 | 953408.9 | 937619.4 | 945872.0 | 1010436.5 | 1097487.0 | 1127415.7 | -0.19 | -1.70 |
| SLC8A1 | 206806.8 | 201350.9 | 202359.4 | 233148.1 | 236760.9 | 225865.7 | -0.19 | -1.52 |
| GLUD1 | 3200581.1 | 3213448.3 | 3304156.7 | 3607595.1 | 3556746.9 | 3885694.8 | -0.19 | -2.26 |
| CHN2 | 441348.3 | 434178.3 | 445224.0 | 505197.6 | 520075.9 | 475658.9 | -0.18 | -1.54 |
| VCAN | 500887.5 | 507414.5 | 510666.8 | 588163.0 | 555325.5 | 571500.3 | -0.18 | -2.12 |
| SNX2 | 197132.8 | 203012.7 | 200133.5 | 228115.3 | 224681.4 | 224537.0 | -0.17 | -1.41 |
| CKMT1 | 2378779.0 | 2481640.8 | 2543528.6 | 2746991.3 | 2666608.8 | 2934578.8 | -0.17 | -1.47 |
| RAB6B | 258591.0 | 258032.8 | 259407.1 | 291372.4 | 285989.8 | 290221.3 | -0.16 | -1.54 |
| CHN2 | 365027.7 | 356770.0 | 370604.5 | 407506.5 | 403702.8 | 399342.7 | -0.15 | -1.33 |
| FABP5 | 1748771.4 | 1707033.1 | 1740929.8 | 1899866.5 | 1911945.9 | 1934046.4 | -0.14 | -2.85 |
| HNRNPD | 1444772.3 | 1488709.5 | 1487494.5 | 1649054.0 | 1580528.8 | 1645335.8 | -0.14 | -1.77 |
| PRXL2A | 751621.0 | 740407.1 | 737473.3 | 674697.3 | 653209.7 | 686635.2 | 0.15 | -1.59 |
| NDUFS1 | 1309563.0 | 1333493.2 | 1361079.0 | 1169215.9 | 1198050.9 | 1237154.7 | 0.15 | -1.77 |
| RAP1GAP2 | 464284.0 | 469359.2 | 473910.5 | 428769.2 | 419140.4 | 415996.4 | 0.16 | -1.77 |
| SLC38A3 | 448585.8 | 443564.3 | 458294.6 | 401761.9 | 409564.1 | 396967.0 | 0.16 | -1.74 |
| LDHA | 1513766.7 | 1562173.1 | 1570710.5 | 1346842.3 | 1375979.9 | 1433509.9 | 0.16 | -1.98 |
| GSTM5 | 369847.4 | 378422.3 | 383779.9 | 330472.5 | 338661.6 | 339207.2 | 0.17 | -1.70 |
| SEMA7A | 202907.6 | 205867.3 | 207958.5 | 183806.5 | 178581.7 | 182716.7 | 0.18 | -1.33 |
| SNAP25 | 3642510.0 | 3371543.0 | 3816013.0 | 3152965.1 | 3130218.1 | 3266134.9 | 0.18 | -1.47 |
| NDUFB2 | 1295961.2 | 1349873.4 | 1306326.8 | 1186499.7 | 1128721.0 | 1168400.2 | 0.18 | -2.87 |
| ALDH1A1 | 1410531.3 | 1392545.0 | 1392846.6 | 1202790.6 | 1197469.2 | 1284470.8 | 0.19 | -2.69 |
| ACSL6 | 529374.3 | 543955.4 | 536779.8 | 462272.1 | 482726.3 | 459648.7 | 0.20 | -2.78 |
| ANXA7 | 281728.8 | 286633.8 | 287546.6 | 236517.4 | 247136.7 | 261078.9 | 0.20 | -1.34 |
| SCCPDH | 874643.0 | 933471.9 | 945249.5 | 782060.7 | 757426.4 | 827552.3 | 0.22 | -2.19 |
| EZR | 402162.4 | 370116.5 | 383084.9 | 329241.6 | 323738.8 | 331563.4 | 0.23 | -2.68 |
| TOM1L2 | 334202.0 | 354386.2 | 338794.7 | 301768.3 | 294457.9 | 274279.8 | 0.24 | -2.05 |
| PDE1C | 2141448.1 | 2187103.7 | 2090634.6 | 1826257.7 | 1752266.3 | 1846983.0 | 0.24 | -6.37 |
| BPNT1 | 401854.9 | 401975.8 | 415302.7 | 362734.7 | 323963.5 | 335730.5 | 0.25 | -2.48 |
| CLIC1 | 225334.7 | 226970.7 | 234470.4 | 198434.9 | 190397.0 | 184387.4 | 0.26 | -2.72 |
| EIF1 | 221857.4 | 236755.3 | 223641.9 | 193318.6 | 185043.4 | 189323.9 | 0.27 | -2.85 |
| IGSF11 | 1025710.2 | 977405.7 | 1055762.1 | 877108.1 | 911320.6 | 756020.8 | 0.27 | -1.68 |
| PRXL2B | 239494.3 | 227431.6 | 228690.0 | 183575.5 | 203436.8 | 191231.7 | 0.27 | -2.19 |
| CAMK2A | 537420.0 | 526636.5 | 532163.7 | 476666.5 | 425460.1 | 423650.6 | 0.27 | -2.74 |
| TFRC | 142233.9 | 142554.3 | 133831.6 | 116938.3 | 114299.7 | 112228.0 | 0.29 | -2.41 |
| SCG2 | 174049.2 | 174467.0 | 194383.3 | 145973.8 | 153690.2 | 138933.4 | 0.31 | -1.95 |
| CMPK2 | 280463.2 | 289340.1 | 314329.5 | 251332.5 | 239684.1 | 222462.9 | 0.31 | -2.37 |
| CHPT1 | 110534.0 | 111084.7 | 98351.5 | 90325.3 | 86431.7 | 80914.7 | 0.31 | -1.32 |
| CD99L2 | 163296.9 | 150292.3 | 151489.1 | 131067.2 | 117578.9 | 125607.7 | 0.31 | -2.14 |
| RPA2 | 97125.4 | 104212.1 | 105708.2 | 84061.6 | 80571.4 | 81646.6 | 0.32 | -2.19 |
| LGALS1 | 587660.8 | 575697.0 | 594936.4 | 485073.4 | 469325.7 | 455465.0 | 0.32 | -8.59 |
| IL1R1 | 220293.2 | 221958.7 | 186287.3 | 171405.0 | 163134.3 | 169305.7 | 0.32 | -1.74 |
| IPCEF1 | 82569.5 | 82900.1 | 84684.6 | 65199.8 | 69958.9 | 65181.1 | 0.32 | -2.00 |
| 1810009N02RIK | 135917.0 | 134312.7 | 124254.0 | 105114.9 | 107308.6 | 96485.0 | 0.35 | -2.51 |
| ENPP2 | 258463.9 | 232651.4 | 261214.0 | 213665.9 | 207041.3 | 166756.4 | 0.36 | -1.41 |
| SLC13A5 | 73038.5 | 68086.7 | 74477.5 | 59306.8 | 54708.7 | 50512.2 | 0.39 | -1.69 |
| PAK1 | 182206.3 | 150114.3 | 149146.3 | 120988.9 | 126470.9 | 116216.2 | 0.40 | -2.19 |
| CARS1 | 85835.9 | 90895.3 | 91231.5 | 68623.2 | 70867.0 | 62099.5 | 0.41 | -2.72 |
| EHD3 | 208687.4 | 220624.4 | 210090.8 | 165980.0 | 150270.0 | 162452.1 | 0.42 | -6.36 |
| THRSP | 43229.9 | 41126.6 | 43598.3 | 32906.2 | 32364.8 | 28974.0 | 0.44 | -1.74 |
| DFFA | 112013.4 | 112239.0 | 124929.8 | 86285.5 | 95267.5 | 74995.7 | 0.44 | -2.05 |
| KCNN2 | 61983.6 | 57191.3 | 57220.9 | 46009.6 | 43403.2 | 40006.1 | 0.45 | -2.22 |
| NCALD | 603186.5 | 621378.6 | 635142.3 | 481558.7 | 458706.3 | 423076.7 | 0.45 | -9.96 |
| NUDCD3 | 125308.6 | 138548.3 | 118524.0 | 91157.1 | 97000.8 | 88408.7 | 0.47 | -3.99 |
| ESD | 418371.2 | 380031.7 | 413742.5 | 268611.4 | 279560.7 | 283175.4 | 0.54 | -15.79 |
| FAH | 240703.7 | 233333.9 | 251782.4 | 169579.3 | 158064.7 | 159403.8 | 0.58 | -15.22 |
| ADORA1 | 84877.9 | 75259.9 | 62027.3 | 59591.9 | 43731.5 | 45342.8 | 0.58 | -1.43 |
| SMARCC1 | 43244.7 | 37166.1 | 44275.1 | 28735.6 | 29021.5 | 22517.5 | 0.64 | -2.04 |
| RIT1 | 17880.4 | 17869.9 | 22270.5 | 11210.9 | 12856.4 | 12926.4 | 0.65 | -1.33 |
| EIF4G3 | 24206.2 | 20142.9 | 19643.7 | 12001.4 | 15655.4 | 11531.2 | 0.71 | -1.33 |
| MEA1 | 36447.8 | 35160.1 | 45604.7 | 28190.0 | 18285.9 | 24490.9 | 0.72 | -1.54 |
| MAL2 | 43409.3 | 47741.0 | 53553.6 | 31644.8 | 32905.1 | 20096.1 | 0.77 | -1.77 |
| NPTXR | 19649.6 | 28507.1 | 27067.9 | 14926.2 | 17020.9 | 11598.7 | 0.79 | -1.34 |
| ME3 | 238970.0 | 252748.8 | 253359.7 | 144890.4 | 143017.6 | 132293.9 | 0.83 | -30.72 |
| NF2 | 58273.7 | 50301.9 | 50985.2 | 34096.8 | 30143.5 | 23612.3 | 0.86 | -4.09 |
| MANEA | 12412.1 | 10279.2 | 9208.0 | 4398.6 | 4033.4 | 6670.6 | 1.08 | -1.41 |
| FMO5 | 25829.5 | 20367.4 | 23965.7 | 10806.8 | 11971.0 | 10402.1 | 1.08 | -5.87 |
| RPGR | 34633.2 | 28763.3 | 27952.2 | 19377.0 | 14768.1 | 8683.5 | 1.09 | -1.88 |
| TRMT10C | 20396.7 | 23823.5 | 18893.3 | 9825.6 | 7524.5 | 12080.1 | 1.10 | -2.92 |
| DAP | 83478.3 | 79562.7 | 61948.7 | 33003.5 | 30962.8 | 13946.4 | 1.53 | -4.97 |

**Supplementary Table 11. Primer sequences for plasmids construction**

| Primer | Sequence（5´-3´） |
| --- | --- |
| SLC38A6-WT-F | ATGGAGGCGTCCTGGGGGA |
| SLC38A6-WT-R | TTATTTATTAATCCAATCAAAAATGATGAGTGCT |
| p.Tyr108Phe-F | ACAGCTGTAACATCTTTTGAAGATCTTGGACTCT |
| p.Tyr108Phe-R | GTCCAAGATCTTCAAAAGATGTTACAGCTGTCTG |
| p.Met281Thr-F | CTTCAAAGAAAAGAACGCAGAATGTTACCAATACAGC |
| p.Met281Thr-F | TGGTAACATTCTGCGTTCTTTTCTTTGAAGGACTT |
| p.Gly318Ser-F | TCAGAATTACTAAAAAGTTATAGTAAATACTTATCAC |
| p.Gly318Ser-R | GTATTTACTATAACTTTTTAGTAATTCTGACTC |
